# Structural Basis of Type 2 Secretion System Engagement between the Inner and Outer Bacterial Membranes

**DOI:** 10.1128/mBio.01344-17

**Published:** 2017-10-17

**Authors:** Iain D. Hay, Matthew J. Belousoff, Trevor Lithgow

**Affiliations:** Infection and Immunity Program, Biomedicine Discovery Institute and Department of Microbiology, Monash University, Clayton, Australia; Max Planck Institute for Terrestrial Microbiology

**Keywords:** *Pseudomonas aeruginosa*, T2SS, protein secretion, protein secretion system, secretin

## Abstract

Sophisticated nanomachines are used by bacteria for protein secretion. In Gram-negative bacteria, the type 2 secretion system (T2SS) is composed of a pseudopilus assembly platform in the inner membrane and a secretin complex in the outer membrane. The engagement of these two megadalton-sized complexes is required in order to secrete toxins, effectors, and hydrolytic enzymes. *Pseudomonas aeruginosa* has at least two T2SSs, with the ancestral nanomachine having a secretin complex composed of XcpQ. Until now, no high-resolution structural information was available to distinguish the features of this *Pseudomonas*-type secretin, which varies greatly in sequence from the well-characterized *Klebsiella*-type and *Vibrio*-type secretins. We have purified the ~1-MDa secretin complex and analyzed it by cryo-electron microscopy. Structural comparisons with the *Klebsiella*-type secretin complex revealed a striking structural homology despite the differences in their sequence characteristics. At 3.6-Å resolution, the secretin complex was found to have 15-fold symmetry throughout the membrane-embedded region and through most of the domains in the periplasm. However, the N1 domain and N0 domain were not well ordered into this 15-fold symmetry. We suggest a model wherein this disordering of the subunit symmetry for the periplasmic N domains provides a means to engage with the 6-fold symmetry in the inner membrane platform, with a metastable engagement that can be disrupted by substrate proteins binding to the region between XcpP, in the assembly platform, and the XcpQ secretin.

## IMPORTANCE

How the outer membrane and inner membrane components of the T2SS engage each other and yet can allow for substrate uptake into the secretin chamber has challenged the protein transport field for some time. This vexing question is of significance because the T2SS collects folded protein substrates in the periplasm for transport out of the bacterium and yet must discriminate these few substrate proteins from all the other hundred or so folded proteins in the periplasm. The structural analysis here supports a model wherein substrates must compete against a metastable interaction between XcpP in the assembly platform and the XcpQ secretin, wherein only structurally encoded features in the T2SS substrates compete well enough to disrupt XcpQ-XcpP for entry into the XcpQ chamber, for secretion across the outer membrane.

## OBSERVATION

Bacterial pathogens use a selection of sophisticated protein secretion nanomachines to deliver toxins, effectors, and hydrolytic enzymes into their environments (1, 2). One of these, the type 2 secretion system (T2SS), evolved from an ancestral nanomachine that also gave rise to the type 4 fimbrial system for locomotion through the secretion—and retraction—of long, fibrous pili (3). These pili are projected from an inner membrane protein complex that functions as an assembly platform and ATP-dependent motor, and from this platform, the pili are driven into a cavernous secretin complex embedded in the outer membrane that spans the periplasm to engage the inner membrane. The T2SS itself encompasses a pilus-like structure, referred to as a pseudopilus since it is not driven outside the cell for locomotion but rather driven in a short motor stroke. The pseudopilus is driven into the T2SS secretin complex by either a piston or a screw-like mechanism, thereby promoting secretion of substrate proteins (toxins, effectors, and hydrolytic enzymes) across the outer membrane. Recent high-resolution structures of the *Vibrio*-type and *Klebsiella*-type secretins showed them to be of 15-fold symmetry (4). These structural breakthroughs highlighted a perplexing problem, given that the secretins interact intimately with 6-fold symmetric structures in the T2SS inner membrane platform (5–7).

*Pseudomonas aeruginosa* is a bacterial pathogen of particular concern to patients with burns, wounds, or cystic fibrosis (8). Species of *Pseudomonas* have two characteristic T2SSs, defined by distinct secretins, XcpQ and HxcQ (9–11). Phylogenetic analysis has suggested that while the T2SS containing HxcQ was acquired more recently by lateral gene transfer from betaproteobacteria, the XcpQ secretin defines the ancestral *Pseudomonas*-type T2SS (10). The T2SS containing XcpQ is responsible for the secretion of at least 19 substrates, most of them hydrolases ranging from proteases to hemolytic lipases that are critical for pathogenesis (11–13). After crossing the inner membrane via the SecYEG or TAT translocases, substrates of the T2SS enter the secretin chamber via an unknown mechanism (5, 6, 9, 14). In order to address how the outer membrane and inner membrane components engage together and yet can allow for substrate uptake, high-resolution structural data are required.

**FIG 1:**
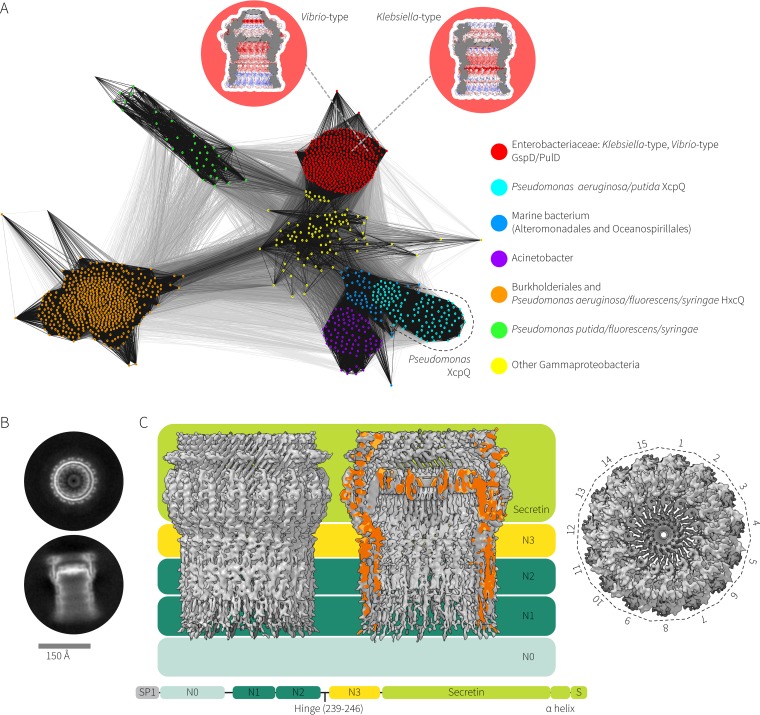
Sequence and structural relationships of XcpQ and other secretins. (A) CLANS analysis (16) graphically depicts homology in large data sets of proteins, utilizing all-against-all pairwise BLAST to cluster representations (colored dots) of individual protein sequences in three-dimensional space. Darker lines between sequences represent similar sequences, and lighter lines represent less similar sequences with an E value cutoff of 1e−10^−10^. The analysis of T2SS secretin sequences (see Table S1 in the supplemental material) shows that proteins from diverse species cluster into six types. The *Klebsiella* type and *Vibrio* type (red) are barely resolved at this scale, and a diverse group of gammaproteobacterial sequences (yellow) are clustered between the *Klebsiella*-type/*Vibrio*-type group and the other groups. The *Pseudomonas*-type HxcQ secretin clusters among a group of betaproteobacterial T2SS sequences from the *Burkholderiales* (orange). *Pseudomonas aeruginosa* XcpQ clusters among three well-resolved groups: one comprised of various XcpQs from various *Pseudomonas* species, one comprised of marine bacteria, and one comprised of the recently described T2SS secretins from species of *Acinetobacter*. Insets show the distinct structures of the *Klebsiella*-type (PDB accession no. 5WQ7) and *Vibrio*-type (PDB accession no. 5WQ8) secretins. (B) XcpQ was purified by size exclusion chromatography, the fraction containing the ~1-MDa complex was frozen, and individual XcpQ particles were visualized by electron microscopy. 2D class averages of top and side views are shown. (C) The 3.6-Å model of XcpQ is shown intact (left) and sliced open sagittally (right) with color coding to highlight the domain architecture. The top-down view illustrates the 15-fold symmetry of XcpQ.

Previous detailed sequence analysis has demonstrated the sequence-based features that distinguish the *Klebsiella*-type secretins from the *Vibrio*-type secretins (15). Cluster Analysis of Sequence data (CLANS) is a sensitive tool to depict sequence relationships (16): in a CLANS plot, each protein is represented as a dot clustering with other proteins of similar sequence. A core set of T2SS secretin sequences was extracted from the InterPro “GspD/PilQ family” (IPR001775) (see Text S1 and Table S1 in the supplemental material), and a CLANS plot of these sequences from diverse bacterial lineages showed the evolutionary distances separating the XcpQ and HxcQ secretins of *Pseudomonas* from other functionally characterized secretins (Fig. 1A). Great sequence drift separates the XcpQ and HxcQ secretins from each other and from the previously characterized *Klebsiella*-type and *Vibrio*-type secretins, while the secretin from the recently discovered T2SS of *Acinetobacter baumannii* (17) clusters with the XcpQ secretin from species of *Pseudomonas* and a grouping of marine bacteria (Fig. 1A). Despite their very close sequence-based clustering, the *Klebsiella*-type and *Vibrio*-type secretins are structurally distinct. Thus, with the XcpQ secretin being even more diverse in sequence and representing an archetype for the *Pseudomonas*-type secretins in general, the secretin complex of *P. aeruginosa* PAO1 was purified for structural analysis by single-particle cryo-electron microscopy.

10.1128/mBio.01344-17.1 TEXT S1 Supplemental materials and methods. Download TEXT S1, DOCX file, 0.02 MB. Copyright © 2017 Hay et al. 2017 Hay et al. This content is distributed under the terms of the Creative Commons Attribution 4.0 International license.

10.1128/mBio.01344-17.5 TABLE S1  Accession details of sequences used for CLANS analysis. Download TABLE S1, PDF file, 0.3 MB. Copyright © 2017 Hay et al. 2017 Hay et al. This content is distributed under the terms of the Creative Commons Attribution 4.0 International license.

XcpQ oligomers were detergent solubilized and purified by size exclusion chromatography, and the ~1-MDa species was shown to be a pure XcpQ multimer when characterized by SDS-PAGE (Fig. S1). XcpQ samples were flash frozen on vitreous ice and were imaged on a Titan Krios transmission electron microscope equipped with a Gatan K2 Summit direct electron detector (Fig. 1B). After analysis of 18,000+ particles, class averages revealed that XcpQ is a pentadecameric secretin complex (Fig. 1B and S2) with no other symmetries observed in the two-dimensional (2D) class averages.

10.1128/mBio.01344-17.2 FIG S1  Expression and purification of the XcpQ multimer. (A) Size exclusion chromatography of the amphipol-stabilized XcpQ multimer after extraction from the total cellular envelope fraction with detergent (SB3-14) and purified with nickel affinity chromatography. (B) The fractions marked by asterisks were assessed by SDS-PAGE and prepared for assessment by electron microscopy as described in Text S1. Download FIG S1, PDF file, 0.1 MB. Copyright © 2017 Hay et al. 2017 Hay et al. This content is distributed under the terms of the Creative Commons Attribution 4.0 International license.

**FIG 2:**
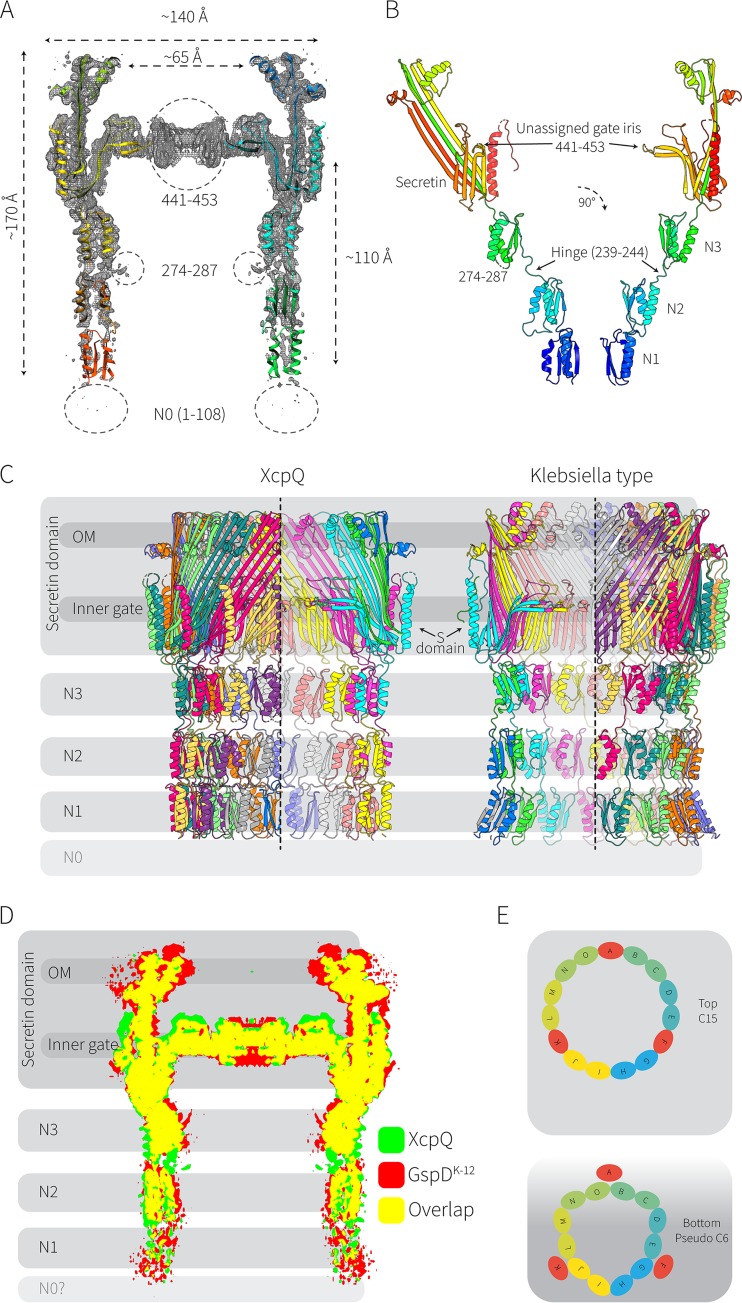
High-resolution structural analysis of XcpQ. (A) XcpQ molecular model (drawn as a cartoon) built into the electron density. Positions where residues could not be assigned are indicated with dotted circles. (B) XcpQ protomer: domain structure showing the secretin domain, the hinge region (residues 239 to 246), and the periplasmic N domains. (C) XcpQ secretin complex and, by way of comparison, the *Klebsiella*-type secretin (PDB accession no. 5WQ7) shown to scale. The similar positioning of the S domains is indicated. OM, outer membrane. (D) The electron densities of XcpQ (green) and the *Klebsiella*-type secretin (red) are overlaid, showing the relatively conserved structure and the progressive deterioration in resolution through the N2, N1, and N0 domains of the structures. (E) Model representing the C15 symmetry observed in the secretin and N3 domain and a model of the pseudo-6-fold symmetry of a hexamer of dimers for the N0-N2 domain.

In the T2SS secretins, each subunit consists of four N domains followed by the highly conserved secretin domain (Pfam PF00263), followed by an S domain. These features, as defined in the secretin structures of the *Klebsiella*-type secretins, are present in the structure of XcpQ (Fig. 2A and B). In the *Klebsiella*-type secretins and *Vibrio*-type secretins, the S domain is necessary for interactions with the pilotin that mediates secretin trafficking to the outer membrane and assembly (15, 18–20). The disposition of the S domain in the XcpQ complex structure is equivalent to that of the other pilotin-dependent secretins (Fig. 2C). Features previously named the “upper chamber,” “inner gate,” and “cap” were resolved (Fig. 2A and B). Comparison of the XcpQ structure to that of the *Klebsiella*-type secretin complex from *Escherichia coli* K-12 strain DH5α (Protein Data Bank [PDB] accession no. 5WQ7) revealed a striking structural homology (Fig. 2C) despite the differences in their sequence characteristics (Fig. 1A).

The N domains of XcpQ contribute to the chamber that needs to dock onto the XcpP subunits of the inner membrane platform (5, 14). At 3.6-Å resolution, individual α-helices and β-strands were resolved throughout the upper part of the secretin structure and within the N3 domain, and the majority of the protein could be visualized (Fig. 2C). There is a relatively unstructured linker or hinge between the N3 and N2 domains (corresponding to residues 239 to 246 in the mature form of XcpQ), where the resolution of the structure starts to decline (Fig. 2D, S2D, and S3). Less well resolved density was present for the N1 domains, and no density for the N0 domains could be observed (Fig. 2D, S2D, and S3). The failure to identify these N domains raises the prospect that this region of the secretin complex, proximal to XcpP in the inner membrane, is not arranged in a uniform 15-fold symmetry.

10.1128/mBio.01344-17.3 FIG S2  Map generation. (A) Representative 2D class averages of XcpQ particles. (B) Fourier shell correlation plot of the 3D map showing a gold-standard resolution of 3.57 Å (FSC = 0.143 criteria). (C) Plot showing the distribution of particle orientations over the azimuth and elevation angles of the final map. (D) Local resolution of the XcpQ model showing deterioration of the resolution in the N-terminal domains (calculated with Relion 2.1 local resolution). Download FIG S2, PDF file, 1.2 MB. Copyright © 2017 Hay et al. 2017 Hay et al. This content is distributed under the terms of the Creative Commons Attribution 4.0 International license.

10.1128/mBio.01344-17.4 FIG S3  Real-space cross-correlation of the fit of the atomic model into the electron density map. Download FIG S3, PDF file, 0.1 MB. Copyright © 2017 Hay et al. 2017 Hay et al. This content is distributed under the terms of the Creative Commons Attribution 4.0 International license.

The T2SS has been an enigma to structural and molecular biologists. In order to drive protein secretion, a secretin like XcpQ must dock to an integral inner membrane complex with 6-fold symmetry (XcpP) (5–7). These intimate interactions raise a further structural dilemma, in that substrates access the T2SS in the periplasm and must therefore interpose between the seals formed by the N domains of the secretin (5, 7). Given the recent findings of Douzi et al. (21), in combination with our data, we suggest a plausible scenario that would resolve this conundrum (Fig. 2E).

The three-dimensional (3D) reconstruction of the XcpQ oligomer was performed under a strict cyclic-15-fold (C15) symmetry. This symmetry restriction was judged to be appropriate by unambiguous 2D class averages of “top-down” projections, clearly and only showing a C15 symmetry (Fig. 1B and C). As can be seen from the 2D class averages (Fig. S2), the best-resolved density is that of the secretin ring and N3 domain, leaving the proximal N domains poorly resolved. This holds true for our 3D model, which during the Fourier reconstruction favored the high-contrast features of the secretin ring and N3 domains. Our data suggest that the N0-N2 domains may not fully conform to the C15 symmetry, and attempts to resolve this part of the structure were not successful. We propose that a pseudo-6-fold symmetry exists in the proximal region of the N domains (Fig. 2E). This might be acquired by the XcpQ dimer-based structures defined by Douzi et al. (21) via a radially symmetric array wherein three N domains are displaced away from the XcpP structure to create a metastable arrangement. The flexibility with which the features pack in order to transition from 15-fold to pseudo-6-fold symmetry provides insight into the mechanism for substrate entry into the T2SS, with the substrate-XcpP interactions competing for the XcpQ-XcpP interactions in order to gain entry into the XcpQ chamber; success in this competition would see the substrate be secreted via pseudopilus movements.

Detailed descriptions of methods are available in Text S1 in the supplemental material, with the map and model parameters for the structures presented as Fig. S2 and Table S2, respectively. T2SS secretin sequence accession numbers and annotations used for the CLANS analysis are presented in Table S1. Electron microscopy data were collected at the Ramaciotti Centre for Cryo-Electron Microscopy, Monash University. All figures were generated with either PyMOL (22) or UCSF Chimera (23).

10.1128/mBio.01344-17.6 TABLE S2  Model parameters. Download TABLE S2, PDF file, 0.05 MB. Copyright © 2017 Hay et al. 2017 Hay et al. This content is distributed under the terms of the Creative Commons Attribution 4.0 International license.

### Accession number(s).

The electron density map has been deposited in the EMDB (EMD-8860). The model of XcpQ has been deposited in the PDB (5WLN).

## References

[1] Costa TR, Felisberto-Rodrigues C, Meir A, Prevost MS, Redzej A, Trokter M, Waksman G 2015 Secretion systems in Gram-negative bacteria: structural and mechanistic insights. Nat Rev Microbiol 13:343–359. doi:10.1038/nrmicro3456. 25978706

[2] Gunasinghe SD, Webb CT, Elgass KD, Hay ID, Lithgow T 2017 Super-resolution imaging of protein secretion systems and the cell surface of gram-negative bacteria. Front Cell Infect Microbiol 7:220. doi:10.3389/fcimb.2017.00220. 28611954 PMC5447050

[3] Hospenthal MK, Costa TRD, Waksman G 2017 A comprehensive guide to pilus biogenesis in Gram-negative bacteria. Nat Rev Microbiol 15:365–379. doi:10.1038/nrmicro.2017.40. 28496159

[4] Yan Z, Yin M, Xu D, Zhu Y, Li X 2017 Structural insights into the secretin translocation channel in the type II secretion system. Nat Struct Mol Biol 24:177–183. doi:10.1038/nsmb.3350. 28067918

[5] Douzi B, Ball G, Cambillau C, Tegoni M, Voulhoux R 2011 Deciphering the Xcp *Pseudomonas aeruginosa* type II secretion machinery through multiple interactions with substrates. J Biol Chem 286:40792–40801. doi:10.1074/jbc.M111.294843. 21949187 PMC3220450

[6] Korotkov KV, Sandkvist M, Hol WG 2012 The type II secretion system: biogenesis, molecular architecture and mechanism. Nat Rev Microbiol 10:336–351. doi:10.1038/nrmicro2762. 22466878 PMC3705712

[7] Wang X, Pineau C, Gu S, Guschinskaya N, Pickersgill RW, Shevchik VE 2012 Cysteine scanning mutagenesis and disulfide mapping analysis of arrangement of GspC and GspD protomers within the type 2 secretion system. J Biol Chem 287:19082–19093. doi:10.1074/jbc.M112.346338. 22523076 PMC3365941

[8] Breidenstein EB, de la Fuente-Núñez C, Hancock RE 2011 *Pseudomonas aeruginosa*: all roads lead to resistance. Trends Microbiol 19:419–426. doi:10.1016/j.tim.2011.04.005. 21664819

[9] Douzi B, Filloux A, Voulhoux R 2012 On the path to uncover the bacterial type II secretion system. Philos Trans R Soc Lond B Biol Sci 367:1059–1072. doi:10.1098/rstb.2011.0204. 22411978 PMC3297435

[10] Durand E, Alphonse S, Brochier-Armanet C, Ball G, Douzi B, Filloux A, Bernard C, Voulhoux R 2011 The assembly mode of the pseudopilus: a hallmark to distinguish a novel secretion system subtype. J Biol Chem 286:24407–24416. doi:10.1074/jbc.M111.234278. 21586577 PMC3129219

[11] Ball G, Antelmann H, Imbert PR, Gimenez MR, Voulhoux R, Ize B 2016 Contribution of the twin arginine translocation system to the exoproteome of *Pseudomonas aeruginosa*. Sci Rep 6:27675. doi:10.1038/srep27675. 27279369 PMC4899797

[12] Filloux A 2011 Protein secretion systems in *Pseudomonas aeruginosa*: an essay on diversity, evolution, and function. Front Microbiol 2:155. doi:10.3389/fmicb.2011.00155. 21811488 PMC3140646

[13] Passmore IJ, Nishikawa K, Lilley KS, Bowden SD, Chung JC, Welch M 2015 Mep72, a metzincin protease that is preferentially secreted by biofilms of *Pseudomonas aeruginosa*. J Bacteriol 197:762–773. doi:10.1128/JB.02404-14. 25488299 PMC4334185

[14] Thomassin JL, Santos Moreno J, Guilvout I, Tran Van Nhieu G, Francetic O 2017 The trans-envelope architecture and function of the type 2 secretion system: new insights raising new questions. Mol Microbiol 105:211–226. doi:10.1111/mmi.13704. 28486768

[15] Dunstan RA, Heinz E, Wijeyewickrema LC, Pike RN, Purcell AW, Evans TJ, Praszkier J, Robins-Browne RM, Strugnell RA, Korotkov KV, Lithgow T 2013 Assembly of the type II secretion system such as found in *Vibrio cholerae* depends on the novel pilotin AspS. PLoS Pathog 9:e1003117. doi:10.1371/journal.ppat.1003117. 23326233 PMC3542185

[16] Frickey T, Lupas A 2004 CLANS: a Java application for visualizing protein families based on pairwise similarity. Bioinformatics 20:3702–3704. doi:10.1093/bioinformatics/bth444. 15284097

[17] Weber BS, Kinsella RL, Harding CM, Feldman MF 2017 The secrets of *Acinetobacter* secretion. Trends Microbiol 25:532–545. doi:10.1016/j.tim.2017.01.005. 28216293 PMC5474180

[18] Dunstan RA, Hay ID, Wilksch JJ, Schittenhelm RB, Purcell AW, Clark J, Costin A, Ramm G, Strugnell RA, Lithgow T 2015 Assembly of the secretion pores GspD, Wza and CsgG into bacterial outer membranes does not require the Omp85 proteins BamA or TamA. Mol Microbiol 97:616–629. doi:10.1111/mmi.13055. 25976323

[19] Nickerson NN, Tosi T, Dessen A, Baron B, Raynal B, England P, Pugsley AP 2011 Outer membrane targeting of secretin PulD protein relies on disordered domain recognition by a dedicated chaperone. J Biol Chem 286:38833–38843. doi:10.1074/jbc.M111.279851. 21878629 PMC3234708

[20] Tosi T, Nickerson NN, Mollica L, Jensen MR, Blackledge M, Baron B, England P, Pugsley AP, Dessen A 2011 Pilotin-secretin recognition in the type II secretion system of *Klebsiella oxytoca*. Mol Microbiol 82:1422–1432. doi:10.1111/j.1365-2958.2011.07896.x. 22098633

[21] Douzi B, Trinh NTT, Michel-Souzy S, Desmyter A, Ball G, Barbier P, Kosta A, Durand E, Forest KT, Cambillau C, Roussel A, Voulhoux R 2017 Unraveling the self-assembly of the *Pseudomonas aeruginosa* XcpQ secretin periplasmic domain provides new molecular insights into type II secretion system secreton architecture and dynamics. mBio 8:e01185-17. doi:10.1128/mBio.01185-17. PMC5646246 29042493

[22] Schrödinger LLC The PyMOL molecular graphics system, version 1.8 Schrödinger LLC, Cambridge, MA.

[23] Pettersen EF, Goddard TD, Huang CC, Couch GS, Greenblatt DM, Meng EC, Ferrin TE 2004 UCSF Chimera—a visualization system for exploratory research and analysis. J Comput Chem 25:1605–1612. doi:10.1002/jcc.20084. 15264254

