# Supplementary material for: Structural Basis of Type 2 Secretion System Engagement between the Inner and Outer Bacterial Membranes
Source: mBio. 2017 Oct 17;8(5):e01344-17. doi: 10.1128/mBio.01344-17 (PMC5646249; doi:10.1128/mBio.01344-17)
Supplement: TEXT S1 [file mbo005173525s1.docx]

**MATERIALS AND METHODS**

***Protein Expression and Purification.***

The sequence of XcpQ was amplified from *P. aeruginosa* PAO1 genomic DNA with the addition of a C-terminal hexahistidine tag. The gene was cloned into the plasmid pET-20b(+). This plasmid was transformed into *E. coli* C43 cells (1). Transformants were grown overnight in LB (Lennox) supplemented with 150 µg mL^-1^ ampicillin and 0.5 % (w/v) glucose. Cells were diluted 1:50 in Terrific broth (100 mM potassium phosphate buffer pH 7.5, 12 g L^-1^ tryptone, 24 g L^-1^ Yeast extract, 5 % w/v glycerol) and incubated at 37 ºC with shaking until an optical density at 600 nm of approximately 0.8, at which point 0.2 mM IPTG was added and incubated at 25 ºC for a further 16 hours.

Cells were harvested by centrifugation at 7,000 ×g and washed once in buffer A (20 mM HEPES, 300 mM NaCl) and resuspended approximately 1:5 in buffer A (v/v). DNase I (10 µg mL^-1^) and then egg white lysozyme (100 µg mL^-1^) was added and cells were incubated for 30 min before they were disrupted with an Avestin Emusliflex C3. Cell lysates were clarified by centrifugation at 7,000 ×g for 15 min. Cell envelopes were isolated from the clarified lysates by centrifugation at 100,000 ×g for 1 hour. Cell envelopes were washed once with a glass homogenizer in buffer A and again centrifuged at 100,000 ×g for 1 hour. The washed envelope fraction was resuspended 1:10 in buffer A (v/v) with a glass homogenizer.

The isolated envelopes were mixed 1:1 with buffer A containing 5 % Sb3-14 and stirred for 30 min. This solution was loaded onto a HisTrap Ni-NTA column (GE Lifesciences). The column was washed with 4 column volumes of buffer A containing 50 mM imidazole then 4 column volumes of buffer A containing 100 mM imidazole. The protein was eluted from the column with buffer A containing 400 mM imidazole. Amphipol A8-35 (Anatrace) was added to the elution fraction (4:1 protein mass ratio) and incubated for 30 min. The XcpQ multimer was purified on a Superose 6 increase 10/300 GL size exclusion column running in buffer A. Fractions eluting from 12.0 - 12.5 ml were concentrated to approximately 0.5 mg mL^-1^ with Amicon Ultra 0.5 mL 100K centrifuge filtration units and flash frozen and stored at -80 ºC until further use.

***Electron Microscopy***

Quantifoil R1.2/1.3 holey carbon grids were washed and coated in a layer of LDAO to prevent the protein complex preferentially adsorbing to the carbon support as previously described (2). Sample (4 µL) was applied to detergent treated grids and frozen hydrated samples were prepared with a Vitrobot Mark IV (FEI, Oregon USA) in a 100 % humidity atmosphere, 2 s blot time and no drain time before plunge freezing into liquid ethane. Grids were imaged on a Titan Krios transmission electron microscope (FEI) at 300 kV at the Ramaciotti Centre for Cryo-Electron Microscopy, Monash University. Data was collected on a Gatan K2 direct electron detector cd as follows: Images were collected at 130,000 × magnification (0.53 Å/pixel in super resolution mode) with 18 frames at 0.4 s per frame with a total dose of 40 e/Å^2^. A random defocus between 0.6-2.5 µm was applied for each micrograph.

***Data Processing***

RELION 2.1 (3) was used as a wrapper to motion correct the movies with dose weighting using MotionCorr 2.1 (4), and estimate CTF with CTFFIND4 (5). Micrographs with acceptable CTF estimations were used to manually pick ~ 1,000 particles which were used to generate 2D class averages used for RELION auto picking. Particles were extracted with a 324 × 324 pixel box size and subjected to multiple rounds of 2D classification in RELION – only C15 symmetry could be observed in 2D class averages. Selected particles were imported in to CryoSPARC (6) for *ab initio* 3D classification and final refinement. A total of 18,009 particles were used to generate a 3.57 Å resolution map. Resolution of the final reconstructions was determined using the gold-standard FSC=0.143 criterion (Supplementary Fig 1). Electron microscopy maps derived in this study are available on the EMDB (EMD-8860).

***Atomic model refinement***

A model of the XcpQ monomer was created using Rosetta (7) and the *E. coli* K-12 GspD model as a template (PDB: 5WQ7). The monomer was symmetrized around the XcpQ EM electron density map using SITUS pdbsymm (8). The resulting model was then subjected to energy minimisation to remove any steric clashes. Fitting the model to the EM electron density map was achieved using the MDFF routine in namd (9). The fitted model was further refined by rounds of manual model building in coot (10) and real space refinement as implemented in the Phenix software package (11). The 3.57 Å model of the *P. aeruginosa* XcpQ secretin derived from this study is available at the PDB (5WLN)..

***Bioinformatic analysis.***

Proteobacteria protein sequences from the InterPro “GspD/PilQ family” (IPR001775) were downloaded and redundancy was minimized with CD-HIT with a 0.98 cut off (12). The sequences were classified by an all-against-all BLAST and clustered based on pairwise similarities and visualized with CLANS with a E value cut off of 1×10^-15^ (13). Those sequences representing the core T2SS secretins presented in Figure 1 were extracted for further analysis and are shown in Supplemental table 1.

***Image Processing***

All images were generated with Pymol or UCSF Chimera (14).

**References**

1. Miroux B, Walker JE. 1996. Over-production of proteins in *Escherichia coli*: mutant hosts that allow synthesis of some membrane proteins and globular proteins at high levels. J Mol Biol 260:289-98.

2. Cheung M, Kajimura N, Makino F, Ashihara M, Miyata T, Kato T, Namba K, Blocker AJ. 2013. A method to achieve homogeneous dispersion of large transmembrane complexes within the holes of carbon films for electron cryomicroscopy. J Struct Biol 182:51-6.

3. Kimanius D, Forsberg BO, Scheres SH, Lindahl E. 2016. Accelerated cryo-EM structure determination with parallelisation using GPUs in RELION-2. Elife 5.

4. Li X, Mooney P, Zheng S, Booth CR, Braunfeld MB, Gubbens S, Agard DA, Cheng Y. 2013. Electron counting and beam-induced motion correction enable near-atomic-resolution single-particle cryo-EM. Nat Methods 10:584-90.

5. Rohou A, Grigorieff N. 2015. CTFFIND4: Fast and accurate defocus estimation from electron micrographs. J Struct Biol 192:216-21.

6. Punjani A, Rubinstein JL, Fleet DJ, Brubaker MA. 2017. cryoSPARC: algorithms for rapid unsupervised cryo-EM structure determination. Nat Methods 14:290-296.

7. Leaver-Fay A, Tyka M, Lewis SM, Lange OF, Thompson J, Jacak R, Kaufman K, Renfrew PD, Smith CA, Sheffler W, Davis IW, Cooper S, Treuille A, Mandell DJ, Richter F, Ban YE, Fleishman SJ, Corn JE, Kim DE, Lyskov S, Berrondo M, Mentzer S, Popovic Z, Havranek JJ, Karanicolas J, Das R, Meiler J, Kortemme T, Gray JJ, Kuhlman B, Baker D, Bradley P. 2011. ROSETTA3: an object-oriented software suite for the simulation and design of macromolecules. Methods Enzymol 487:545-74.

8. Wriggers W. 2012. Conventions and workflows for using Situs. Acta Crystallogr D Biol Crystallogr 68:344-51.

9. Chan KY, Trabuco LG, Schreiner E, Schulten K. 2012. Cryo-electron microscopy modeling by the molecular dynamics flexible fitting method. Biopolymers 97:678-86.

10. Emsley P, Lohkamp B, Scott WG, Cowtan K. 2010. Features and development of Coot. Acta Crystallogr D Biol Crystallogr 66:486-501.

11. Adams PD, Afonine PV, Bunkoczi G, Chen VB, Davis IW, Echols N, Headd JJ, Hung LW, Kapral GJ, Grosse-Kunstleve RW, McCoy AJ, Moriarty NW, Oeffner R, Read RJ, Richardson DC, Richardson JS, Terwilliger TC, Zwart PH. 2010. PHENIX: a comprehensive Python-based system for macromolecular structure solution. Acta Crystallogr D Biol Crystallogr 66:213-21.

12. Li W, Godzik A. 2006. Cd-hit: a fast program for clustering and comparing large sets of protein or nucleotide sequences. Bioinformatics 22:1658-9.

13. Frickey T, Lupas A. 2004. CLANS: a Java application for visualizing protein families based on pairwise similarity. Bioinformatics 20:3702-4.

14. Pettersen EF, Goddard TD, Huang CC, Couch GS, Greenblatt DM, Meng EC, Ferrin TE. 2004. UCSF Chimera--a visualization system for exploratory research and analysis. J Comput Chem 25:1605-12.
