# Supplementary material for: Structural Basis of Type 2 Secretion System Engagement between the Inner and Outer Bacterial Membranes
Source: mBio. 2017 Oct 17;8(5):e01344-17. doi: 10.1128/mBio.01344-17 (PMC5646249; doi:10.1128/mBio.01344-17)
Supplement: TABLE S1 [file mbo005173525st1.pdf]

Pseudomonas aeruginosa/putida XcpQ

Entry	Protein names	Gene names	Organism	Length
P35818	Type II secretion system protein D	xcpQ	Pseudomonas aeruginosa (strain ATCC 15692 / DSM 22644 / CIP 104116 / JCM 14847 / LMG 12228 / 1C / PRS 101 / PAO1)	658
A0A023WT13	General secretion pathway protein GspD	UIB01_13090	Pseudomonas stutzeri (Pseudomonas perfectomarina)	644
A0A031IS26	Protein XcpQ	xcpQ BW43_04544	Pseudomonas sp. RIT357	633
A0A061JVJ3	General secretion pathway protein D	B597_004215	Pseudomonas stutzeri KOS6	644
A0A078LRA9	General secretion pathway protein D	xcpQ BN1079_01133	Pseudomonas saudiphocaensis	751
A0A083UI50	General secretion pathway protein GspD	HA62_24155	Pseudomonas putida (Arthrobacter siderocapsulatus)	642
A0A086BZS6	General secretion pathway protein GspD	G039_0308875	Pseudomonas aeruginosa VRFPA01	658
A0A098FPU9	Type II secretion system protein D	xcpQ PXNS11_230067	Pseudomonas xanthomarina	645
A0A099RQK4	General secretion pathway protein GspD	DP64_14310	Pseudomonas sp. HMP271	749
A0A0A1GCB6	General secretion pathway protein D	MT1_1942	Pseudomonas sp. MT-1	758
A0A0A1HW65	General secretion pathway protein D	BN844_4825	Pseudomonas sp. SHC52	641
A0A0A1YK28	General secretion pathway protein GspD	TMS3_0112355	Pseudomonas taeanensis MS-3	650
A0A0A4GLY0	General secretion pathway protein GspD	N005_06695	Pseudomonas mediterranea CFBP 5447	638
A0A0A8RG33	Type II secretion system protein D	xcpQ1 PAMH19_1963	Pseudomonas aeruginosa	702
A0A0A8RQQ6	Type II secretion system protein D	xcpQ3 PAMH19_5274	Pseudomonas aeruginosa	776
A0A0C2F3Y4	General secretion pathway protein D	UCMB321_0557	Pseudomonas batumici	622
A0A0C2SCW4	General secretion pathway protein GspD	QX25_03625	Pseudomonas stutzeri (Pseudomonas perfectomarina)	746
A0A0C5EF45	Uncharacterized protein	TO66_26230	Pseudomonas sp. MRSN12121	632
A0A0D0MPK9	General secretion pathway protein GspD	RL74_21425	Pseudomonas fluorescens	638
A0A0D6BKU1	General secretion pathway protein D	gspD4 POS17_3902	Pseudomonas sp. Os17	639
A0A0D6S4G0	General secretion pathway protein D	SZ55_4490	Pseudomonas sp. FeS53a	632
A0A0D6SHG7	General secretion pathway protein D	SZ55_3386	Pseudomonas sp. FeS53a	644
A0A0D9AKT1	General secretion pathway protein GspD	UF78_12255	Pseudomonas stutzeri (Pseudomonas perfectomarina)	757
A0A0E1AVX8	General secretion pathway protein D	U769_09375	Pseudomonas aeruginosa MTB-1	657
A0A0E1EAU8	General secretion pathway protein GspD	EY04_26175	Pseudomonas chlororaphis	633
A0A0F4TSD0	General secretion pathway protein GspD	VC35_10020	Pseudomonas fluorescens	639
A0A0F4XGY0	General secretion pathway protein GspD	VP02_23980	Pseudomonas kilonensis	649

A0A0H3YW05	General secretion pathway protein D (Type II secretion system protein GspD)	AB691_2648<br>AYK87_07600<br>BFM99_20650	Pseudomonas stutzeri (Pseudomonas perfectomarina)	644
A0A0J6GCJ4	General secretion pathway protein D	SAMN04489800_0495	Pseudomonas deceptionensis	641
A0A0J6GPS7	General secretion pathway protein GspD	TU78_17680	Pseudomonas taetrolens	633
A0A0J6IH51	General secretion pathway protein GspD	TU84_01370	Pseudomonas helleri	638
A0A0M2UNX6	General secretion pathway protein GspD	V520_02135	Pseudomonas putida KG-4	613
A0A0M3CLH3	General secretion pathway protein GspD	PU99_26825	Pseudomonas putida (Arthrobacter siderocapsulatus)	642
A0A0M4QBX2	Type II secretion system protein GspD	AOC04_18600<br>BOH73_11500	Pseudomonas versuta	640
A0A0N0E5F6	Type II secretion system protein D	PF66_01114	Pseudomonas fuscovaginae	632
A0A0N9VP76	Type II secretion system protein GspD	AO353_12545	Pseudomonas fluorescens	632
A0A0N9X7D9	Type II secretion system protein GspD	AO356_09345	Pseudomonas fluorescens	641
A0A0P7CVI4	Type II secretion system protein GspD	HB4184_20700	Pseudomonas putida (Arthrobacter siderocapsulatus)	613
A0A0P8WQ89	Type II secretion system protein D	gspD AN403_2294	Pseudomonas fluorescens	639
A0A0Q0SXX2	Type II secretion system protein GspD	AQS70_04715	Pseudomonas endophytica	638
A0A0Q4NJA7	Type II secretion system protein GspD	ASF02_02265	Pseudomonas sp. Leaf58	613
A0A0Q4S6T2	Type II secretion system protein GspD	ASF15_18080	Pseudomonas sp. Leaf83	650
A0A0Q8BB55	Type II secretion system protein GspD	ASD60_01415	Pseudomonas sp. Root562	642
A0A0Q8I0W2	Type II secretion system protein GspD	ASD91_08965	Pseudomonas sp. Root68	639
A0A0R0CYU5	General secretion pathway protein GspD	ABB27_03470	Stenotrophomonas terrae	639
A0A0S8YUJ4	Type II secretion system protein GspD	ASE98_07450	Pseudomonas sp. Leaf48	639
A0A0T6UMT5	Type II secretion system protein GspD	AO726_11305	Pseudomonas sp. TTU2014-080ASC	638
A0A0T6UP61	Type II secretion system protein GspD	AO726_12045	Pseudomonas sp. TTU2014-080ASC	650
A0A0T6V454	Type II secretion system protein GspD	AO741_16310	Pseudomonas sp. TTU2014-105ASC	644
A0A0T6VHL7	Type II secretion system protein GspD	AO735_18130	Pseudomonas sp. TTU2014-096BSC	748
A0A0T6VRR0	Type II secretion system protein GspD	AO729_13020	Pseudomonas sp. TTU2014-066ASC	746
A0A0V2R4Y0	Type II secretion system protein GspD	AO896_11040	Pseudomonas aeruginosa	634
A0A0V2RM22	Type II secretion system protein GspD	AO896_00185	Pseudomonas aeruginosa	769
A0A0X1T5Y3	Type II secretion system protein GspD	AWM79_20160	Pseudomonas agarici	636
A0A126NHM6	Type II secretion system protein GspD	AXG53_06935	Stenotrophomonas sp. KCTC 12332	639
A0A142BAN4	General secretion pathway protein D	gspD2 EZMO1_1659	Endozoicomonas montiporae CL-33	708
A0A142IQK5	Type II secretion system protein GspD	A0T30_09500	Pseudomonas alcaligenes	652
A0A165PV67	Type II secretion system protein GspD	A3710_08510	Pseudomonas stutzeri (Pseudomonas perfectomarina)	646
A0A165ZWV4	Type II secretion system protein GspD	TK06_18730	Pseudomonas fluorescens	640

A0A176NN03	Type II secretion system protein GspD	APS14_01435	Pseudomonas thivervalensis	644
A0A177YAX0	Type II secretion system protein GspD	A3K88_08850	Pseudomonas putida (Arthrobacter siderocapsulatus)	613
A0A1B3CJZ2	Type II secretion system protein GspD	AXG94_17335	Pseudomonas corrugata	638
A0A1B3CKV1	Type II secretion system protein GspD	A7317_01340	Pseudomonas fluorescens	633
A0A1B3E4W5	General secretion pathway protein GspD	THL1_2032	Pseudomonas sp. TCU-HL1	655
A0A1B3EDE9	General secretion pathway protein GspD	THL1_5039	Pseudomonas sp. TCU-HL1	631
A0A1C2DBX4	Type II secretion system protein GspD	BBI09_20520	Pseudomonas xanthomarina	747
A0A1D9IMH7	Type II secretion system protein GspD	AA042_12040	Pseudomonas sp. BS-2016	628
A0A1E4UU08	Type II secretion system protein GspD	A7D25_20305	Pseudomonas sp. 21C1	652
A0A1E4V6S2	Type II secretion system protein GspD	A7D27_24935	Pseudomonas sp. 1D4	632
A0A1E4VDD8	Type II secretion system protein GspD	A7D27_16970	Pseudomonas sp. 1D4	644
A0A1E4VYV2	Type II secretion system protein GspD	A7D27_01330	Pseudomonas sp. 1D4	776
A0A1G3DZJ5	Type II secretion system protein GspD	A3J71_12570	Pseudomonadales bacterium RIFCSPHIGHO2_02_FULL_60_43	763
A0A1G3E9I4	Type II secretion system protein GspD	A2Y50_01105	Pseudomonadales bacterium RIFCSPLOWO2_12_59_9	646
A0A1G3ED51	Type II secretion system protein GspD	A3J71_18675	Pseudomonadales bacterium RIFCSPHIGHO2_02_FULL_60_43	649
A0A1G3FQK4	Type II secretion system protein GspD (Fragment)	A3J25_17450	Pseudomonadales bacterium RIFCSPLOWO2_02_FULL_63_210	533
A0A1G6U0Y4	General secretion pathway protein D	SAMN05216575_101364	Pseudomonas alcaliphila	647
A0A1G7HUH1	General secretion pathway protein D	SAMN05216575_105187	Pseudomonas alcaliphila	744
A0A1G7WX13	General secretion pathway protein D	SAMN05216605_10388	Pseudomonas abietaniphila	633
A0A1H1QT23	General secretion pathway protein D	SAMN05216598_1040	Pseudomonas asplenii	635
A0A1H1RCS0	General secretion pathway protein D	SAMN05216585_1966	Pseudomonas chlororaphis	633
A0A1H2PBG3	General secretion pathway protein D	SAMN05216558_4746	Pseudomonas vancouverensis	639
A0A1H4WEB1	General secretion pathway protein D	SAMN04490188_0326	Pseudomonas kilonensis	642
A0A1H7RLY4	General secretion pathway protein D	SAMN05216214_11532	Pseudomonas hussainii	777
A0A1I0UI39	General secretion pathway protein D	SAMN05216263_11343	Pseudomonas otitidis	774
A0A1I5RAR9	General secretion pathway protein D	SAMN05216601_11512	Pseudomonas composti	645
A0A1K1PZ44	General secretion pathway protein D	SAMN03159439_02495	Pseudomonas sp. NFACC04-2	641
A0A1K1RH77	General secretion pathway protein D	SAMN05660640_02933	Pseudomonas sp. NFACC16-2	640
A0A1M5RTG2	General secretion pathway protein D	SAMN02744645_3240	Pseudomonas xanthomarina DSM 18231	756
A0A1N6ZH02	General secretion pathway protein D	SAMN05421672_11829	Pseudomonas flexibilis	640
A0A1Q8EJE0	Type II secretion system protein GspD	BTN82_24540	Pseudomonas chlororaphis	633
A0A1R0GC92	Type II secretion system protein GspD	AU074_25590	Pseudomonas sp. ATCC PTA-122608	631

A0A1T1HIA7	Type II secretion system protein GspD	MF4836_33190	Pseudomonas sp. MF4836	635
A4XWG6	General secretion pathway protein D	Pmen_2927	Pseudomonas mendocina (strain ymp)	645
A4XZZ1	General secretion pathway protein D	Pmen_4160	Pseudomonas mendocina (strain ymp)	748
F4DZZ5	General secretion pathway protein D	MDS_1730	Pseudomonas mendocina (strain NK-01)	647
G8Q2P1	XcpQ	xcpQ PSF113_0438	Pseudomonas fluorescens F113	646
I3UUA8	General secretion pathway protein D	YSA_04271	Pseudomonas putida ND6	613
I4JMM4	General secretion pathway protein D	YO5_15425	Pseudomonas stutzeri TS44	749
J2E945	General secretion pathway protein D	xcpQ Pchl3084_4936	Pseudomonas chlororaphis subsp. aureofaciens 30-84	633
J2F8L3	General secretion pathway protein D	gspD PflQ2_5268	Pseudomonas fluorescens Q2-87	637
J2N6L1	General secretion pathway protein D	PMI20_01446	Pseudomonas sp. GM17	633
J2QYI9	General secretion pathway protein D	PMI26_03562	Pseudomonas sp. GM33	642
J2RHM2	General secretion pathway protein D	PMI28_04085	Pseudomonas sp. GM48	639
J2UI02	General secretion pathway protein D	PMI32_00650	Pseudomonas sp. GM60	642
J3HG02	General secretion pathway protein D	PMI34_01316	Pseudomonas sp. GM74	639
K5Y9A9	General secretion pathway protein D	C211_12597	Pseudomonas sp. Chol1	749
L0GNZ0	General secretion pathway protein D	Psest_2577	Pseudomonas stutzeri RCH2	644
L8MFP5	General secretion pathway protein D	KF707C_50390 ppKF707_4261	Pseudomonas pseudoalcaligenes KF707 = NBRC 110670	632
L8MP58	General secretion pathway protein D	KF707C_35460 ppKF707_1735	Pseudomonas pseudoalcaligenes KF707 = NBRC 110670	646
O52657	XqhA	xqhA	Pseudomonas aeruginosa	713
Q9ZFY0	Outer membrane secretion protein Q	xcpQ	Pseudomonas alcaligenes	649
S6BFC1	Type II secretion system protein XcpQ	xcpQ PCA10_20220	Pseudomonas resinovorans NBRC 106553	650
U2BF23	General secretion pathway protein D	N878_13960	Pseudomonas sp. EGD-AK9	648
U2UKR4	Uncharacterized protein	O999_25585	Pseudomonas putida LF54	612
U3H3Q0	General secretion pathway protein D	L682_26640	Pseudomonas alcaligenes OT 69	632
U3HCK6	General secretion pathway protein D	L682_10325	Pseudomonas alcaligenes OT 69	652
U3HG92	Uncharacterized protein	L682_10935	Pseudomonas alcaligenes OT 69	789
U3HNS7	General secretion pathway protein D	L686_22905	Pseudomonas stutzeri MF28	756
W6VIN1	General secretion pathway protein D	PMI25_001461	Pseudomonas sp. GM30	630
W8Q6K7	General secretion pathway protein GspD	CD58_02160	Pseudomonas brassicacearum	641
W8RVF2	General secretion pathway protein GspD	CH92_13350	Pseudomonas stutzeri (Pseudomonas perfectomarina)	756
W9TIK1	General secretion pathway protein D	xcpQ BAY1663_00422	Pseudomonas sp. BAY1663	644

Marine bacteria (Alteromonadales and Oceanospirillales)

Entry	Protein names	Gene names	Organism	Length
A0A139DF37	General secretion pathway protein D, GspD	AWU57_3477	Marinobacter sp. T13-3	528
A0A1H5W723	General secretion pathway protein D	SAMN04489856_103226	Oleiphilus messinensis	633
A0A0K6INX8	Type II secretion system protein D	Ga0061065_10887	Marinomonas fungiae	629
A0A1A8TG45	Type II secretion system protein D	xcpQ MAQ5080_02254	Marinomonas aquimarina	631
A0A1A8TSZ0	Type II secretion system protein D	xcpQ MSP8886_04318	Marinomonas spartinae	616
A0A1C3JN30	Type II secretion system protein D	xcpQ MGA5115_00632	Marinomonas gallaica	629
A3YCA1	General secretion pathway protein D	MED121_18255	Marinomonas sp. MED121	616
A0A095S6V4	General (Type II) secretion pathway protein D	T9A_02950	Alcanivorax jadensis T9	686
A0A0C5VT74	Type II secretory pathway, component PulD	YC6258_04496	Gynuella sunshinyii YC6258	625
A0A0H4I1Z9	General secretion pathway protein GspD	ABA45_04945	Marinobacter psychrophilus	640
A0A0J7M1M9	Type II secretion system protein D	Msub_11189	Marinobacter subterrani	651
A0A0K1UFK8	General secretion pathway protein GspD	ACP86_01965	Marinobacter sp. CP1	650
A0A0P1IZV8	General secretion pathway protein D / Type II secretion outermembrane pore forming protein (PulD)	BN2364_1649	Alcanivorax dieselolei	701
A0A0P7ZJQ0	Type II secretion system protein D	gspD HLUCCX14_05135	Marinobacter excellens HL-55	652
A0A0P8B2W9	T2SS system secretin GspD	gspD-2 HLUCCO03_15260	Marinobacter sp. HL-58	650
A0A0T6W2J7	Type II secretion system protein GspD	AQ621_12235	Marinobacter sp. P4B1	651
A0A137SDJ8	Type II secretion outermembrane pore forming protein	J122_1598	Marinobacter excellens LAMA 842	651
A0A139DCG6	General secretion pathway protein D, GspD	AWU57_4424	Marinobacter sp. T13-3	629
A0A139DNL3	General secretion pathway protein D	AWU57_547	Marinobacter sp. T13-3	647
A0A165RQY1	Type II secretion system protein GspD	A3737_06805	Oleiphilus sp. HI0065	648
A0A166I3K4	Type II secretion system protein GspD	A3715_07385	Oleiphilus sp. HI0009	656
A0A166XKS3	Type II secretion system protein GspD	A3759_05180	Thalassolituus sp. HI0120	632
A0A176H9J8	Type II secretion system protein GspD	A3741_03060	Oleiphilus sp. HI0069	651
A0A176HKY4	Type II secretion system protein GspD	A3746_12680	Oleibacter sp. HI0075	631
A0A1D9GI41	Type II secretion system protein GspD	BKP64_02630	Marinobacter salinus	651
A0A1E3CCM3	Type II secretion system protein GspD	A6779_07220	Marinobacter adhaerens	650
A0A1E7PTI9	Type II secretion system protein GspD	BG841_00650	Marinobacter sp. X15-166B	651
A0A1H5YCB7	General secretion pathway protein D	SAMN04515663_11064	Alcanivorax sp. DSM 26293	684
A0A1L6KGG5	Type II secretion system protein GspD	CN03_15005	Thalassolituus oleivorans	636

A0A1M6RZC4	General secretion pathway protein D	SAMN05216369_1751	Marinobacter antarcticus	651
A0A1Q8G399	Type II secretion system protein GspD	AWH63_09050	Marinobacter sp. C18	650
A0A1S2CEU2	Type II secretion system protein GspD	BCA33_15855	Marinobacter sp. AC-23	651
A1U289	General secretion pathway protein D	Maqu_2027	Marinobacter hydrocarbonoclasticus (strain ATCC 700491 / DSM 11845 / VT8)	649
A3J9R2	Type II secretory pathway, component PulD	MELB17_18269	Marinobacter sp. ELB17	640
A4BES0	Type II secretory pathway, component PulD	MED297_02717	Reinekea blandensis MED297	653
B4X5A7	General secretion pathway protein D	ADG881_2997	Alcanivorax sp. DG881	694
E4PGK8	Type II and III secretion system protein	HP15_2355	Marinobacter adhaerens (strain HP15)	663
F2K4U3	General secretion pathway protein D	Marme_3370	Marinomonas mediterranea (strain ATCC 700492 / JCM 21426 / NBRC 103028 / MMB-1)	623
L0WCH0	General (Type II) secretion pathway protein D	A11A3_08505	Alcanivorax hongdengensis A-11-3	689
M7D5C8	Type II and III secretion system protein	MSNKSG1_08553	Marinobacter santoriniensis NKSG1	653
N6W366	Type II and III secretion system protein	J057_01084	Marinobacter nanhaiticus D15-8W	659
Q0VSG7	General (Type II) secretion pathway protein D	ABO_0433	Alcanivorax borkumensis (strain ATCC 700651 / DSM 11573 / NCIMB 13689 / SK2)	710
Q1N1M8	Type II secretory pathway, component PulD	RED65_03250	Bermanella marisrubri	640
Q2SDG3	Type II secretory pathway, component PulD	HCH_04610	Hahella chejuensis (strain KCTC 2396)	647
R4YRD3	General secretion pathway protein D	gspD OLEAN_C04930	Oleispira antarctica RB-8	634
R8B675	Type II and III secretion system protein	MARLIPOL_01008	Marinobacter lipolyticus SM19	651
U7FLP8	Secretin	Q670_04785	Alcanivorax sp. P2S70	690
W5YJW5	General secretion pathway protein D	AU14_12900	Marinobacter similis	656
A0A072NF41	General secretion pathway protein D	D777_02049	Marinobacter nitratireducens	652
A0A0B4XG46	General (Type II) secretion pathway protein D	S7S_03010	Alcanivorax pacificus W11-5	685
A0A095TML6	General (Type II) secretion pathway protein D	Y5S_03081	Alcanivorax nanhaiticus	685

<u>Acinetobacter</u>

Entry	Protein names	Gene names	Organism	Length
A0A009F596	Type II secretion system protein D	gspD J504_2311	Acinetobacter baumannii 348935	751
A0A009GBQ0	Type II secretion system protein D	gspD J517_3813	Acinetobacter baumannii 118362	758
A0A009GD25	Type II secretion system protein D	gspD J508_1914	Acinetobacter sp. 1289694	758
A0A009HVA4	Type II secretion system protein D	gspD J512_0964	Acinetobacter baumannii 1295743	756
A0A009JNZ9	Type II secretion system protein D	gspD J537_3195	Acinetobacter baumannii 1437282	756
A0A010KB51	Type II secretion system protein D	gspD J582_0887	Acinetobacter sp. 1566109	758
A0A011C7F5	Type II secretion system protein D	gspD J623_3605	Acinetobacter sp. 1245249	756
A0A013SJK9	Type II secretion system protein D	gspD J663_3480	Acinetobacter sp. 826659	752
A0A031LIM3	General secretion pathway protein GspD	CL42_15495	Acinetobacter sp. Ver3	795
A0A062BR64	Type II secretion system protein D	gspD J577_3095	Acinetobacter sp. 263903-1	751
A0A072CTL1	General secretion pathway protein GspD	DT74_20445	Acinetobacter sp. ETR1	749
A0A0A3W7N7	General secretion pathway protein D	GW12_09140	Acinetobacter sp. HR7	746
A0A0A8TJK7	General secretion pathway protein D		Acinetobacter bereziniae (Acinetobacter genomosp. 10)	749
A0A0A8XK32	Putative general secretion pathway protein	P23_3555	Acinetobacter calcoaceticus	758
A0A0B2UBD4	General secretion pathway protein GspD	DH17_06755	Acinetobacter oleivorans	758
A0A0B2XSR9	General secretion pathway protein GspD	NT90_14420	Acinetobacter baumannii	758
A0A0F3LGI2	General secretion pathway protein GspD	VH96_12500	Acinetobacter indicus	749
A0A0J8SQJ0	General secretion pathway protein GspD	ACS72_11860	Acinetobacter sp. VT 511	750
A0A0L0J0M1	Type II and III secretion system protein	AFK20_10895	Enhydrobacter aerosaccus	813
A0A0M1I7U3	General secretion pathway protein GspD	ABW55_03670	Acinetobacter sp. C15	767
A0A0M4HB54	General secretion pathway protein GspD	AMQ28_09350	Acinetobacter sp. TTH0-4	755
A0A0M4T352	Type II secretion system protein GspD	AOC03_08965	Psychrobacter urativorans	740
A0A0Q9ZGL8	Type II and III secretion system protein	AK822_11260	Psychrobacter sp. P11F6	748
A0A0U3SU07	General secretion pathway protein GspD	RZ95_15645	Acinetobacter johnsonii XBB1	786
A0A0W8H430	General secretion pathway protein GspD	AAU60_01405	Acinetobacter johnsonii	786
A0A109WC14	Type II secretion system protein GspD	AXE82_09055	Moraxella osloensis	808
A0A124EZJ1	Type II secretion system protein GspD	NDM229_18605	Acinetobacter bereziniae (Acinetobacter genomosp. 10)	749
A0A127H310	Type II and III secretion system protein	AK825_11215	Psychrobacter sp. P11G5	754
A0A137Y674	Type II secretion system protein GspD	AYL20_15270	Acinetobacter haemolyticus	754
A0A144PYB8	General secretion pathway protein D	A3K91_2177	Psychrobacter alimentarius	751
A0A145WN30	Type II secretion system protein GspD	AMD27_01160	Acinetobacter sp. TGL-Y2	758

A0A151Y5P3	Type II secretion system protein GspD	AZH43_05890	Acinetobacter sp. ANC 4149	747
A0A172YL17	Type II secretion system protein GspD	A3K93_00755	Acinetobacter sp. NCu2D-2	744
A0A173MTY7	General secretion pathway protein D	MOSL_0425	Moraxella osloensis	807
A0A178G045	Type II secretion system protein GspD	AY606_11760	Acinetobacter sp. SFB	756
A0A178G071	Type II secretion system protein GspD	AY608_06645	Acinetobacter sp. SFC	757
A0A178GQP4	Type II secretion system protein GspD	AY605_03025	Acinetobacter sp. SFD	754
A0A197F9D9	Type II secretion system protein GspD	A7325_10965	Psychrobacter sp. SHUES1	754
A0A1B2M327	Type II secretion system protein GspD	BFG52_15105	Acinetobacter larvae	739
A0A1B8PYH9	Type II secretion system protein GspD	A9299_11860	Moraxella osloensis	818
A0A1B8U870	General secretion pathway protein GspD	NG55_19945	Acinetobacter gyllenbergii	758
A0A1D9CYK6	Bacterial type II and III secretion system family protein	AOT82_1233	Psychrobacter sp. AntiMn-1	752
A0A1E3GII5	Type II secretion system protein GspD	A9Z54_12915	Acinetobacter sp. 51m	754
A0A1E5KLD1	Type II secretion system protein GspD	BAX61_10215	Psychrobacter sp. B29-1	748
A0A1E7RB90	Type II secretion system protein GspD	BJD20_00580	Acinetobacter proteolyticus	758
A0A1E8DZ41	Type II secretion system protein GspD	BJN41_13070	Acinetobacter towneri	752
A0A1F2VAH4	Type II secretion system protein GspD	A2003_00780	Acinetobacter sp. GWC1_38_13	751
A0A1L6KR38	Type II secretion system protein GspD	AHTJS_15140	Acinetobacter haemolyticus	736
A0A1Q3NB59	Type II secretion system protein GspD	BGO19_00465	Acinetobacter sp. 38-8	753
A0A1Q8DWA1	Type II secretion system protein GspD	BTV98_01285	Psychrobacter sp. Cmf 22.2	752
A0A1Q8E487	Type II secretion system protein GspD	BTV99_01165	Psychrobacter sp. Rd 27.2	747
A0A1R4IBD0	General secretion pathway protein D / Type II secretion outermembrane pore forming protein (PulD)	CZ794_01350	Psychrobacter sp. JB385	748
A0A1S8CTY8	Type II secretion system protein GspD	BKE30_12185	Alkanindiges sp. H1	761
A0A1T0CCW3	Type II secretion system protein GspD	B0682_07150	Moraxella lincolnii	746
A0A1T1GVZ5	Type II secretion system protein GspD	B1202_10010	Acinetobacter sp. ANC 4945	743
A0A1U6GPI7	Type II and III secretion system protein	DABAL43B_2250	Psychrobacter sp. DAB_AL43B	751
C0VHI7	General secretion pathway protein D	gspD HMPREF0023_0606	Acinetobacter sp. ATCC 27244	735
K8ZS27	Type II secretion system protein D	gspD ACINWC141_0299	Acinetobacter sp. WC-141	756
K9BX38	Type II secretion system protein D	gspD ACINWC323_0294	Acinetobacter sp. WC-323	754
L2F973	Type II and III secretion system protein	MOMA_02935	Moraxella macacae 0408225	764
N8P3G5	Type II secretion system protein D	F994_00419	Acinetobacter bohemicus ANC 3994	755
N8Q2H0	Type II secretion system protein D	F989_02147	Acinetobacter parvus NIPH 1103	752
N8R937	Type II secretion system protein D	F991_00267	Acinetobacter sp. CIP-A165	737

N8RTW3	Type II secretion system protein D	F988_00345	Acinetobacter parvus DSM 16617 = CIP 108168	752
N8UTJ1	Type II secretion system protein D	F971_03633	Acinetobacter sp. NIPH 758	752
N8VNZ7	Type II secretion system protein D	F968_03542	Acinetobacter sp. NIPH 817	752
N8W0J0	Type II secretion system protein D	F972_00129	Acinetobacter sp. CIP 102529	750
N8Y6H9	Type II secretion system protein D	F960_03766	Acinetobacter gerneri DSM 14967 = CIP 107464	771
N9C4T4	Type II secretion system protein D	F942_00684	Acinetobacter ursingii ANC 3649	758
N9CJK4	Type II secretion system protein D	F947_02367	Acinetobacter towneri DSM 14962 = CIP 107472	751
N9DNT4	Type II secretion system protein D	F941_00259	Acinetobacter bouvetii DSM 14964 = CIP 107468	746
N9E3P1	Type II secretion system protein D	F938_04452	Acinetobacter bereziniae LMG 1003 = CIP 70.12	748
N9E7E6	Type II secretion system protein D	F933_02116	Acinetobacter beijerinckii CIP 110307	749
N9EE27	Type II secretion system protein D	F934_00094	Acinetobacter beijerinckii ANC 3835	754
N9FSH9	Type II secretion system protein D	F925_00960	Acinetobacter lwoffii NCTC 5866 = CIP 64.10 = NIPH 512	750
N9N781	Type II secretion system protein D	F895_01307	Acinetobacter sp. CIP 64.2	753
N9QF07	Type II secretion system protein D	F887_00325	Acinetobacter sp. NIPH 2100	753
N9TF44	Type II secretion system protein D	F902_00391	Acinetobacter sp. CIP 70.18	751
Q1Q9C2	Type II and III secretion system protein	Pcryo_1954	Psychrobacter cryohalolentis (strain K5)	750
Q6FFA1	Putative general secretion pathway protein	ACIAD0294	Acinetobacter baylyi (strain ATCC 33305 / BD413 / ADP1)	772
R8Y3E9	Type II secretion system protein D	F935_02812	Acinetobacter calcoaceticus ANC 3811	758
R9AW94	Type II secretion system protein D	I593_03456	Acinetobacter tandoii DSM 14970 = CIP 107469	751
S3MTL6	Type II secretion system protein D	F945_03610	Acinetobacter rudis CIP 110305	764
U4TE52	General secretion pathway protein D	M917_0363	Psychrobacter aquaticus CMS 56	749
V2V0T8	Type II secretion system protein D	F990_01501	Acinetobacter tjernbergiae DSM 14971 = CIP 107465	751

Enterobacteriaceae (Klebsiella-type, Vibrio-type: GspD/PulD)

Entry	Protein names	Gene names	Organism	Length
P45758	Putative type II secretion system protein D	gspD	Escherichia coli (strain K12)	650
B7UI37	Putative type II secretion protein	gspD	Escherichia coli O127:H6 (strain E2348/69 / EPEC)	686
W1GSM0	General secretion pathway protein D		Klebsiella pneumoniae ISC21	444
P45779	Type II secretion system protein D (T2SS protein D) (Cholera toxin secretion protein EpsD) (General secretion pathway protein D)	epsD VC_2733	Vibrio cholerae serotype O1 (strain ATCC 39315 / El Tor Inaba N16961)	674
A0A090QX80	General secretion pathway protein D	JCM19237_7007	Photobacterium aphoticum	474
P15644	Type II secretion system protein D (T2SS protein D) (General secretion pathway protein D) (Pullulanase secretion envelope PulD)	pulD	Klebsiella pneumoniae	660
P31700	Type II secretion system protein D (T2SS protein D) (General secretion pathway protein D) (Pectic enzymes secretion protein OutD)	outD	Dickeya chrysanthemi (Pectobacterium chrysanthemi) (Erwinia chrysanthemi)	712
P31701	Type II secretion system protein D (T2SS protein D) (General secretion pathway protein D) (Pectic enzymes secretion protein OutD)	outD	Pectobacterium carotovorum subsp. carotovorum (Erwinia carotovora subsp. carotovora)	650
P31780	Type II secretion system protein D (T2SS protein D) (General secretion pathway protein D)	exeD	Aeromonas hydrophila	678
P45778	Type II secretion system protein D (T2SS protein D) (General secretion pathway protein D)	exeD	Aeromonas salmonicida	678
Q01565	Type II secretion system protein D (T2SS protein D) (General secretion pathway protein D) (Pectic enzymes secretion protein OutD)	outD Dda3937_02415	Dickeya dadantii (strain 3937) (Erwinia chrysanthemi (strain 3937))	710
A0A010QAY7	Type II secretion system protein D	H978DRAFT_3598	Alteromonas sp. ALT199	681
A0A017I8C3	Type II secretion system protein D	AC26_3562	Escherichia coli 1-176-05_S3_C2	650
A0A023RES7	General secretion pathway protein D	B224_0272	Aeromonas media WS	682
A0A023V4E9	General secretion pathway protein GspD	CFNIH1_04300	Citrobacter freundii CFNIH1	664
A0A023ZPP9	General secretion pathway protein D	gspD	Lonsdalea quercina subsp. populi	705
A0A026VBM5	General secretion pathway protein GspD	BW70_07780	Escherichia coli O174:H8 str. 04-3038	686
A0A034THX2	General secretion pathway protein D	JCM18904_2927	Vibrio sp. JCM 18904	673
A0A060VGT4	PulD protein	pulD KpST82_0729	Klebsiella pneumoniae	660
A0A061Q0X5	General secretion pathway protein D	JCM19052_3484	Vibrio sp. JCM 19052	681
A0A066PBW0	Type II secretion system protein D	AF34_01374	Lelliottia amnigena CHS 78	640
A0A066RWY6	General secretion pathway protein GspD	EA58_08005	Photobacterium galatheae	671
A0A071LUJ7	General secretion pathway protein GspD	DT73_15915	Mangrovibacter sp. MFB070	656

A0A073KNZ6	General secretion protein D	SXM_0179	Shewanella xiamenensis	704
A0A078LJ98	T2SS protein D	gspD BN1086_02066	Citrobacter koseri (Citrobacter diversus)	640
A0A081LNK5	General secretion pathway protein GspD	AL345_18395	Aeromonas caviae (Aeromonas punctata)	684
A0A084A0U4	Type II secretion system protein D	pulD SRDD_21190	Serratia sp. DD3	660
A0A085A4L6	General secretion pathway protein D	gspD GTGU_03029	Trabulsiella guamensis ATCC 49490	657
A0A085TJH1	Type II secretion system protein D	gspD DN31_2176	Vibrio cholerae	675
A0A085U413	General secretion pathway protein D	nADLYRO1b_2696	Yersinia ruckeri	660
A0A085UB85	General secretion pathway protein D	CSF007_14575 nADLYRO1b_218	Yersinia ruckeri	674
A0A087KL83	General secretion pathway protein GspD	IV04_24035	Serratia sp. Ag1	655
A0A090K3H2	General secretion pathway protein D	epsD MVIS_0264	Moritella viscosa	697
A0A091A379	General secretion pathway protein GspD	JM66_17390	Aeromonas salmonicida	678
A0A093SMQ1	General secretion pathway protein GspD	KP17_09835	Pectobacterium carotovorum subsp. carotovorum (Erwinia carotovora subsp. carotovora)	684
A0A093T2A1	General secretion pathway protein GspD	KP22_12470	Pectobacterium betavasculorum	684
A0A094J9L9	General secretion pathway protein GspD	HR45_15080	Shewanella mangrovi	708
A0A094TTR7	General secretion pathway protein GspD	KU75_21515	Pectobacterium carotovorum subsp. odoriferum	684
A0A099L6T6	Uncharacterized protein	ND16A_0920	Thalassotalea sp. ND16A	708
A0A0A0HC84	General secretion pathway protein D (Putative general secretion pathway protein D)	EL75_0371 EL79_0390 EL80_0382	Escherichia coli	654
A0A0A3FEG1	Type II secretion system protein GspD	BWP24_15985	Vibrio campbellii	675
A0A0A3Z9X2	General secretion pathway protein GspD	NG99_09635	Erwinia typographi	625
A0A0A8FJA4	General secretion pathway protein D	W909_13875	Dickeya zeae EC1	713
A0A0B2TE34	General secretion pathway protein GspD	OI69_03120	Pectobacterium carotovorum (Erwinia carotovora)	651
A0A0B3WXM1	General secretion pathway protein GspD	RC79_07615	Pectobacterium carotovorum subsp. brasiliense	684
A0A0B5J335	General secretion pathway protein GspD	TE10_06540	Raoultella ornithinolytica (Klebsiella ornithinolytica)	646
A0A0B6FA65	Type II secretion system protein D	gspD AW19_2330	Yersinia frederiksenii Y225	657
A0A0B7GFX3	General secretory pathway component, cryptic (Type II secretion system protein GspD)	gspD BBD63_04435 BBD64_04435 BBD65_04430 KVR801_380056	Klebsiella variicola	651
A0A0C3R4L0	General secretion pathway protein GspD	DB48_02875	Shewanella sp. cp20	704
A0A0D0HV79	General secretion pathway protein GspD	SN10_01905	Vibrio harveyi (Beneckea harveyi)	675
A0A0D0QPG7	Contig_52, whole genome shotgun sequence	RW26_15455	Aeromonas sp. L_1B5_3	682
A0A0D1DNM8	Whole genome shotgun sequence	H323_05590	Vibrio parahaemolyticus VP766	674
A0A0D1FP35	Whole genome shotgun sequence	H320_07875	Vibrio parahaemolyticus 49	675

A0A0D8CSU2	General secretion pathway protein GspD	SG35_20260	Thalassomonas actiniarum	694
A0A0D8D699	General secretion pathway protein GspD	SG34_24010	Thalassomonas viridans	694
A0A0E0WW85	General secretion pathway protein D	A225_1532	Klebsiella michiganensis E718	659
A0A0E1NTF5	General secretion pathway protein D (Type II secretion system protein D)	gspD YPA_0453 CH58_3299	Yersinia pestis bv. Antiqua (strain Antiqua)	640
A0A0E2LL48	General secretion pathway protein D	M001_04490	Aeromonas veronii Hm21	679
A0A0E8XH01	General secretion pathway protein D	pulD_2 ERS008478_01498	Yersinia wautersii	640
A0A0F0SVA4	General secretion pathway protein GspD	SS44_22750	Enterobacter cloacae subsp. cloacae	642
A0A0F0YGF6	General secretion pathway protein D (General secretion pathway protein GspD) (Type II secretion system protein GspD)	gspD AN674_0202860 AN693_0201415 SAMEA3181516_01548 SS33_04580	Enterobacter kobei	643
A0A0F1BG95	General secretion pathway protein GspD	SS37_01745	Enterobacter cloacae complex sp. 35699	642
A0A0F1WYQ9	Type II secretion system protein GspD	ASV07_04910	Enterobacter cloacae complex sp. SMART_763	640
A0A0F3ZPH9	General secretion pathway protein GspD	SG66_03415	Enterobacter asburiae	641
A0A0F4BML7	General secretion pathway protein GspD	SG71_13125	Enterobacter cloacae complex sp. CIDEIMsCOL9	643
A0A0F5SJP8	General secretion pathway protein GspD	WG82_02330	Citrobacter amalonaticus	661
A0A0F5V9F4	General secretion pathway protein GspD	KY46_17300	Photobacterium halotolerans	672
A0A0F5ZW34	General secretion pathway protein GspD	RN22_13605	Grimontia sp. AD028	672
A0A0F6TU89	General secretion pathway protein GspD	F384_04065	Citrobacter amalonaticus Y19	640
A0A0G4K093	General secretion pathway protein D	BN1221_03895	Brenneria goodwinii	657
A0A0H0GIB9	General secretion pathway protein GspD	WR15_04770	Escherichia coli	645
A0A0H3F5A6	General secretion pathway protein D	Rahaq_0337	Rahnella sp. (strain Y9602)	659
A0A0H3I117	General secretion pathway protein D	W5S_1295	Pectobacterium sp. (strain SCC3193)	695
A0A0H5GE01	General secretion pathway protein D	outD ERS137941_02317	Yersinia enterocolitica	648
A0A0J1GNR5	General secretion pathway protein GspD	ABT58_07950	Photobacterium aphoticum	671
A0A0J1H2W0	General secretion pathway protein GspD	ABT57_20390	Photobacterium ganghwense	671
A0A0J1H7W1	General secretion pathway protein GspD	ABT56_04125	Photobacterium aquae	672
A0A0J1VEC7	Type II secretion system protein D	SP99_04103	Enterobacter sp. BIDMC92	661
A0A0J6QGX9	General secretion pathway protein GspD	VK95_02170	Leclercia sp. LK8	640
A0A0J8GPG7	General secretion pathway protein GspD	XM47_13265	Catenovulum maritimum	721
A0A0J8V8D4	General secretion pathway protein GspD	AB733_18045	Photobacterium swingsii	671
A0A0J8VHT9	General secretion pathway protein GspD	ACH50_17395	Franconibacter sp. DJ34	659
A0A0K4TJQ6	Type II secretion protein EtpD	pulD_2 pulD ERS085383_04345	Escherichia coli	656

		ERS085404_04863 ERS085416_03928		
A0A0L0ATR0	General secretion pathway protein GspD	AC791_06350	Klebsiella sp. RIT-PI-d	665
A0A0M0IPF9	General secretion pathway protein GspD	AKJ18_04385	Vibrio xuii	675
A0A0M3EZN9	General secretion pathway protein GspD	OA46_19395	Enterobacter cloacae	643
A0A0M9C7P8	General secretion pathway protein GspD	ACX03_14295	Vibrio parahaemolyticus	674
A0A0N0ZIF0	General secretion pathway protein GspD	ADS78_06805	Idiomarina abyssalis	696
A0A0N8UIS7	General secretion pathway protein GspD	XV92_05055	Vibrio metoecus	674
A0A0P8Y4W9	General secretion pathway protein GspD	JI57_03220	Psychromonas sp. PRT-SC03	660
A0A0Q0IXK5	Type II secretion system protein D	pulD AN944_01753	Shewanella sp. P1-14-1	715
A0A0Q4N2L8	Type II secretion system protein GspD	ASF13_04890	Erwinia sp. Leaf53	646
A0A0T7NSN1	General secretion pathway protein D	outD ERS008498_01674	Yersinia enterocolitica	657
A0A0T9LEY7	General secretion pathway protein D	outD ERS008491_02484	Yersinia kristensenii	660
A0A0T9LF55	General secretion pathway protein D	outD ERS137967_02681	Yersinia nurmii	680
A0A0T9M6P4	General secretion pathway protein D	pulD_2 ERS008566_01872	Yersinia pseudotuberculosis	640
A0A0T9M9I9	General secretion pathway protein D	outD ERS008530_02210	Yersinia intermedia	658
A0A0T9P0L6	General secretion pathway protein D	pulD ERS008670_00633	Yersinia massiliensis	654
A0A0T9P9F4	General secretion pathway protein D	outD ERS008529_01454	Yersinia pekkanenii	661
A0A0T9PFC4	General secretion pathway protein D	outD ERS008472_01886	Yersinia kristensenii	657
A0A0T9QVX4	General secretion pathway protein D	outD ERS008531_03149	Yersinia frederiksenii	658
A0A0T9SFD6	General secretion pathway protein D	outD ERS137951_02170	Yersinia enterocolitica	648
A0A0T9SWN1	General secretion pathway protein D	pulD ERS137951_04103	Yersinia enterocolitica	658
A0A0T9SZ71	General secretion pathway protein D	pulD ERS008727_01455	Yersinia enterocolitica	653
A0A0T9TJI1	General secretion pathway protein D	outD ERS008545_03010	Yersinia frederiksenii	651
A0A0T9TT10	General secretion pathway protein D	outD ERS137965_01753	Yersinia aldovae	641
A0A0T9TY23	General secretion pathway protein D	outD ERS008460_01815	Yersinia kristensenii	666
A0A0T9UWL5	General secretion pathway protein D	pulD ERS137965_03928	Yersinia aldovae	657
A0A0T9UYN2	General secretion pathway protein D	outD ERS008521_03909	Yersinia frederiksenii	658
A0A0T9UZU0	General secretion pathway protein D	outD ERS008547_03839	Yersinia intermedia	661
A0A0U1HAI9	General secretion pathway protein D	outD_1 outD_2 ERS008580_01032 ERS008580_01048	Yersinia enterocolitica	648
A0A0U1HWS0	General secretion pathway protein D	outD ERS008555_03458	Yersinia rohdei	654
A0A0U5VA85	Pullulanase secretion envelope pulD	pulD ERS381490_01824	Serratia marcescens	655
A0A0V9E2F2	Type II secretion system protein GspD	APT89_14685	Enterobacter sp. 50588862	644

A0A0V9JQ40	Type II secretion system protein GspD	APU02_09015	Citrobacter sp. 50677481	642
A0A0W0AMI2	General secretion pathway protein GspD	VO68_11640 VO70_10735	Aeromonas salmonicida	678
A0A0W0AW81	General secretion pathway protein GspD	VO69_18440	Aeromonas salmonicida	678
A0A0W7U1E1	Type II secretion system protein GspD	ATO46_17505	Aeromonas schubertii	679
A0A0X4ETS4	Type II secretion system protein GspD	AWI28_12400	Enterobacter cloacae complex sp. GN04363	642
A0A0X6Z4I0	Type II secretion system protein GspD	ABF69_0219500	Enterobacter cloacae complex sp. GN02468	643
A0A126PRE0	Type II secretion system protein GspD	AW879_16350	Enterobacter cloacae	640
A0A135IAT9	Type II secretion system protein GspD	ATN88_21065	Enterovibrio coralii	654
A0A137SBB9	General secretion pathway protein D	AKG98_1032	Moritella sp. JT01	697
A0A145L6I9	General secretion pathway protein D	pulD SAMEA2273332_04757	Enterobacter cloacae	661
A0A152V1A7	Type II secretion system protein GspD	ARC77_28485 AU473_28895	Escherichia coli	655
A0A156SPW1	General secretion pathway protein D	gspD SAMEA2273318_02754	Enterobacter cloacae	641
A0A159YYQ5	General secretion pathway protein GspD	NF29_20295	Enterobacter cloacae	641
A0A162UIG8	Type II secretion system protein GspD	A3466_14900	Enterobacter cloacae complex sp. GN06232	639
A0A170AUN8	General secretion pathway protein D	pulD SAMEA2273575_01987	Klebsiella oxytoca	660
A0A175VJT6	Type II secretion system protein GspD	LCR_11910	Aeromonas enteropelogenes (Aeromonas trota)	677
A0A178KPQ7	Type II secretion system protein GspD	A3K86_00825	Photobacterium jeanii	672
A0A181XRE7	General secretion pathway protein D	pulD_2 SAMEA2273573_03578	Klebsiella oxytoca	660
A0A191W0U5	Type II secretion system protein GspD	PL14_00815	Vibrio anguillarum (Listonella anguillarum)	676
A0A198GMN0	General secretion pathway protein D	M987_02540	Enterobacter soli ATCC BAA-2102	643
A0A1A5WP53	Type II secretion system protein GspD	A9196_04725	Aeromonas dhakensis	677
A0A1A9FAN0	Type II secretion system protein GspD	A8A57_07400	Lelliottia amnigena (Enterobacter amnigenus)	641
A0A1B1KM35	Type II secretion system protein D	pulD Q5A_005120	Serratia plymuthica PRI-2C	655
A0A1B7HT13	General secretion pathway protein D	M979_1546	Buttiauxella noackiae ATCC 51607	653
A0A1B7IQB9	General secretion pathway protein D	M975_1820	Buttiauxella brennerae ATCC 51605	653
A0A1B8YY38	Type II secretion system protein GspD	A9R12_19395	Aeromonas hydrophila	677
A0A1C2C679	Type II secretion system protein GspD	A9165_03025	Alishewanella sp. HH-ZS	697
A0A1C7PT17	Type II secretion system protein GspD	A9R10_06530	Aeromonas piscicola	679
A0A1D3CB07	Type II secretion system protein GspD	AN659_0205835	Enterobacter sp. ST121:950178628	640
A0A1E3E5Q8	Type II secretion system protein GspD	BC455_04075	Vibrio harveyi (Beneckea harveyi)	678
A0A1E3MD81	Type II secretion system protein GspD	BGK46_15510	Salinivibrio sp. DV	659

A0A1E4VT81	Type II secretion system protein GspD	A9G06_00685	Aeromonas sp. DNP9	679
A0A1E5CDI3	Type II secretion system protein GspD	A1OK_06490	Enterovibrio norvegicus FF-454	671
A0A1E5DB26	Type II secretion system protein GspD	A130_10015	Vibrio genomosp. F6 str. FF-238	676
A0A1E5E5Y9	Type II secretion system protein GspD	A1QC_04125	Vibrio rumoiensis 1S-45	674
A0A1E5FLW5	Type II secretion system protein GspD	A1QY_00125	Vibrio ordalii 12B09	677
A0A1H3ZU22	General secretion pathway protein D	SAMN02982996_01316	Lonsdalea quercina	705
A0A1I5RZW2	General secretion pathway protein D	SAMN03084138_02714	Enterovibrio norvegicus DSM 15893	672
A0A1J5N2Y8	Type II secretion system protein GspD	BM565_05300	Gammaproteobacteria bacterium MedPE	700
A0A1N6HWR2	Type II secretion system protein D (GspD)	SAMN05444724_2354	Salinivibrio sp. ES.052	672
A0A1N7BUZ4	Type II secretion system protein D (GspD)	SAMN05892873_15212	Aeromonas veronii	680
A0A1N7DW06	Type II secretion system protein D (GspD)	SAMN05444841_11046	Enterobacter kobei	666
A0A1N7VZ79	Type II secretion system protein D	AF54_01529	Serratia marcescens BIDMC 81	654
A0A1Q5VPN8	Type II secretion system protein GspD	BJP24_14820	Aeromonas allosaccharophila	678
A0A1Q6CMK1	Type II secretion system protein GspD	BI291_08460	Thalassotalea sp. PP2-459	680
A0A1Q8F171	Type II secretion system protein GspD	BTN33_18115	Aeromonas veronii	633
A0A1Q9H0E5	Type II secretion system protein GspD	BIT28_05835	Photobacterium sp. 13-12	669
A0A1Q9HAA4	Type II secretion system protein GspD	BIY22_12515	Vibrio panuliri	672
A0A1R4B794	Type II secretion system protein D	pulD VPAL9027_02790	Vibrio sp. CECT 9027	673
A0A1S2AKA2	Type II secretion system protein GspD	BEH76_14665	Shewanella algae	707
A0A1S2CRF4	Type II secretion system protein GspD	BJD16_04200	Aeromonas sobria	674
A0A1S8CER9	Type II secretion system protein GspD	BMI79_20245	Rahnella sp. J11-6	657
A0A1T4UAM9	General secretion pathway protein D	SAMN02745132_01169	Enterovibrio nigricans DSM 22720	672
A0A1U6Y4M2	General secretion pathway protein GspD	V469_20260	Aeromonas hydrophila J-1	678
A1SR56	General secretion pathway protein D	Ping_0097	Psychromonas ingrahamii (strain 37)	662
A6FGZ5	Putative type II secretory pathway, component EpsD	PE36_23882	Moritella sp. PE36	699
A7MZB6	Uncharacterized protein	VIBHAR_00601	Vibrio campbellii (strain ATCC BAA-1116 / BB120)	658
A8GJQ5	General secretion pathway protein D	Spro_4251	Serratia proteamaculans (strain 568)	660
A8T6E5	General secretion pathway protein D	AND4_17629	Vibrio sp. AND4	675
B2VI08	General secretion pathway protein D	outD ETA_07920	Erwinia tasmaniensis (strain DSM 17950 / CIP 109463 / Et1/99)	646
C4L998	General secretion pathway protein D	Tola_0367	Tolumonas auensis (strain DSM 9187 / TA4)	685
C4S9F5	General secretion pathway protein D	ymoll0001_4770	Yersinia mollaretii ATCC 43969	625
C4SKN3	Type II secretion system protein D	gspD DJ58_4014	Yersinia frederiksenii ATCC 33641	657

C4U164	General secretion pathway protein D	ykris0001_29060	Yersinia kristensenii ATCC 33638	635
C6C305	General secretion pathway protein D	Dd703_1470	Dickeya paradisiaca (strain Ech703) (Dickeya dadantii (strain Ech703))	714
C6CQG9	General secretion pathway protein D	Dd1591_1299	Dickeya chrysanthemi (strain Ech1591) (Dickeya zeae (strain Ech1591))	716
C6DAQ5	General secretion pathway protein D	PC1_2860	Pectobacterium carotovorum subsp. carotovorum (strain PC1)	640
D2T508	Type II secretion system protein outD	outD EPYR_00811	Erwinia pyrifoliae (strain DSM 12163 / CIP 106111 / Ep16/96)	645
D2TMH8	Putative T2SS protein D	gspD ROD_44971	Citrobacter rodentium (strain ICC168) (Citrobacter freundii biotype 4280)	662
D2ZAD4	General secretion pathway protein D	gspD ENTCAN_05409	Enterobacter cancerogenus ATCC 35316	643
D4HZX1	Type II secretion system protein outD	outD EAMY_2866	Erwinia amylovora (strain CFBP1430)	645
E3G250	General secretion pathway protein D	Entcl_2937	Enterobacter lignolyticus (strain SCF1)	647
F5RTL6	General secretion pathway protein D	gspD HMPREF9086_0971	Enterobacter hormaechei ATCC 49162	640
G8LGV2	Putative general secretion pathway protein D	gspD EcWSU1_01436	Enterobacter cloacae EcWSU1	676
G9YZX5	General secretion pathway protein D	HMPREF0880_00771	Yokenella regensburgei ATCC 43003	669
H2IRA1	General secretion pathway protein D	Rahaq2_0355	Rahnella aquatilis (strain ATCC 33071 / DSM 4594 / JCM 1683 / NBRC 105701 / NCIMB 13365 / CIP 78.65)	661
L0MMW6	General secretion pathway protein D	D781_3847	Serratia marcescens FGI94	635
M4U3E0	General secretion pathway protein D	PCNPT3_00895	Psychromonas sp. CNPT3	658
N9VIR3	General secretion pathway protein D	G114_14071	Aeromonas diversa CDC 2478-85	680
Q6D2I6	General secretion pathway protein D	outD ECA3109	Pectobacterium atrosepticum (strain SCRI 1043 / ATCC BAA-672) (Erwinia carotovora subsp. atroseptica)	650
R1GZM8	General secretion pathway protein D	G113_01424	Aeromonas molluscorum 848	677
R8WR60	Type II secretion system protein D	WC7_04167	Citrobacter sp. KTE151	661
R9PF56	General secretion pathway protein D	AALB_0095	Agarivorans albus MKT 106	702
S4YQC0	General secretion pathway protein D	M621_05230	Serratia plymuthica S13	655
T0PNY9	General secretion pathway protein D	K931_09985	Aeromonas salmonicida subsp. pectinolytica 34mel	677
U0ETS3	General secretion pathway protein D	N779_24935	Vibrio coralliilyticus OCN008	678
U2M9C3	General secretion pathway protein D	L581_3189	Serratia fonticola AU-AP2C	648
U6ZCV0	Uncharacterized protein	A544_2877	Dickeya solani D s0432-1	726
V3TNZ0	Uncharacterized protein	Ser39006_03464	Serratia sp. (strain ATCC 39006)	709
V5ZBE3	Type II secretion system protein outD	outD EPIR_2906	Erwinia piriflorinigrans CFBP 5888	645
V9ZZF9	General secretion pathway protein D	AH4AK4_3643	Aeromonas hydrophila 4AK4	685
W0LA40	General secretion pathway protein GspD	Z042_03970	Chania multitudinisentens RB-25	655

A0A034TYM6	General secretion pathway protein D	JCM18905_3059	Vibrio sp. JCM 18905	672
A0A063KMA0	General secretion pathway protein GspD	DC53_15910	Pseudoalteromonas fuliginea	687
A0A066UWK3	General secretion pathway protein GspD	VFDL14_15210	Vibrio fortis	674
A0A075P3V8	General secretion pathway protein GspD	EP13_18580	Alteromonas australica	681
A0A081KGN5	General secretion pathway protein GspD	GV64_23640	Endozoicomonas elysicola	746
A0A084CNJ6	General secretion pathway protein D	epsD CF67_19010	Candidatus Photodesmus blepharus	674
A0A084TH91	General secretion pathway protein GspD	HW45_00495	Vibrio sp. ER1A	674
A0A085PK17	General secretion pathway protein D		Vibrio cholerae	674
A0A087IFK6	General secretion pathway protein GspD	JS86_21435	Vibrio vulnificus	673
A0A090ITI0	General secretion pathway protein D	epsD AWOD_I_2557	Aliivibrio wodanis	672
A0A090S6J7	General secretion pathway protein D	JCM19235_7071	Vibrio maritimus	658
A0A090U2E2	General secretion pathway protein D	JCM19240_4565	Vibrio maritimus	658
A0A094ITI5	General secretion pathway protein GspD	IDAT_05625	Idiomarina atlantica	702
A0A094IUK1	General secretion pathway protein GspD	IDSA_00960	Idiomarina salinarum	702
A0A097QNZ1	Type II secretion system protein GspD	BA953_08550	Vibrio coralliilyticus	676
A0A099KNP1	General secretion pathway protein D	GAB14E_0511	Colwellia psychrerythraea (Vibrio psychroerythus)	695
A0A099KU78	General secretion pathway protein D	ND2E_1518	Colwellia psychrerythraea (Vibrio psychroerythus)	697
A0A099LC46	General secretion pathway protein D	ND6B_0080	Pseudoalteromonas sp. ND6B	688
A0A099LRY2	General secretion pathway protein GspD	EA26_01415	Vibrio navarrensis	680
A0A0A5HVK1	General secretion pathway protein GspD	NM06_17045	Vibrio sinaloensis	678
A0A0A5I4P5	General secretion pathway protein GspD	NM22_18805	Vibrio tubiashii	675
A0A0A6QKM0	General secretion pathway protein GspD	NM09_18205	Vibrio caribbeanicus	677
A0A0A7EEN0	General secretion pathway protein GspD	OM33_04470	Pseudoalteromonas sp. OCN003	688
A0A0B2JQ19	General secretion pathway protein GspD	PL71_03555	Pseudoalteromonas flavipulchra NCIMB 2033 = ATCC BAA-314	687
A0A0B3XRA0	General secretion pathway protein GspD	RJ41_12945	Alteromonas marina	681
A0A0B7JGT0	General secretory pathway component, cryptic	gspD PPBDW_I60022	Photobacterium phosphoreum ANT-2200	672
A0A0B8QSV5	General secretion pathway protein D	JCM19241_1774	Vibrio ishigakensis	677
A0A0B9GYP0	General secretion pathway protein GspD	RJ45_20825	Photobacterium gaetbulicola	673
A0A0C1QIJ8	General secretion pathway protein GspD	JF50_01195	Pseudoalteromonas luteoviolacea	686
A0A0C2NYS8	General secretion pathway protein GspD	OJ16_03070	Vibrio renipiscarius	673
A0A0C3DFT5	General secretion pathway protein GspD	SU60_15285	Vibrio mytili	673
A0A0D8LHW0	General secretion pathway protein GspD	UB36_18030	Photobacterium damselae subsp. damselae (Listonella damsela)	674

A0A0D8MIS0	General secretion pathway protein GspD	UB39_16625	Photobacterium angustum	675
A0A0D8Q070	General secretion pathway protein GspD	UB37_07800	Photobacterium iliopiscarium	672
A0A0E0YJD7	General secretion pathway protein D	AMEC673_18935	Alteromonas macleodii (strain English Channel 673)	683
A0A0E3VZD8	General secretion pathway protein D	gspD HBTMEAM1__210088	Candidatus Hamiltonella defensa (Bemisia tabaci)	686
A0A0F2H8Y3	General secretion pathway protein GspD	UF06_21900	Vibrio sp. S234-5	672
A0A0F4NID0	General secretion pathway protein GspD	TW84_23100	Vibrio neptunius	678
A0A0F4NLW4	General secretion pathway protein GspD	TW81_04640	Vibrio galatheae	675
A0A0F4P8Q5	General secretion pathway protein GspD	TW75_06470	Pseudoalteromonas piscicida	688
A0A0F4PQ47	General secretion pathway protein GspD	TW72_01430	Pseudoalteromonas ruthenica	688
A0A0F4QDE6	General secretion pathway protein GspD	TW77_21685	Pseudoalteromonas rubra	686
A0A0F5AR84	General secretion pathway protein GspD	WN56_07425	Salinivibrio sp. KP-1	665
A0A0H0Y1F2	General secretion pathway protein GspD	VVYB158_11225	Vibrio vulnificus CladeA-yb158	672
A0A0H2MTE5	General secretion pathway protein GspD	ZX61_07875	Vibrio sp. VPAP30	676
A0A0K6H6Q4	Type II secretion system protein D	Ga0061064_1510	Idiomarina woesei	730
A0A0L0EW91	General secretion pathway protein GspD (Type II secretion system protein GspD)	AC626_03250 AT705_13705	Pseudoalteromonas rubra	686
A0A0L1L710	General secretion pathway protein GspD	ACS79_15805	Vibrio lentus	674
A0A0L7YJQ7	General secretion pathway protein GspD	ACS86_19330	Vibrio alginolyticus	677
A0A0M0HL95	General secretion pathway protein GspD	AKJ17_13010	Vibrio nereis	674
A0A0M0HX21	General secretion pathway protein GspD	AKJ31_15165	Vibrio hepatarius	672
A0A0M0M7W7	General secretion pathway protein GspD	WH43_03715	Rheinheimera sp. KL1	693
A0A0M2T603	General secretion pathway protein GspD	SD53_08530	Rheinheimera mesophila	697
A0A0M2V2P2	General secretion pathway protein GspD	WG68_14970	Arsukibacterium ikkense	688
A0A0M2VC79	General secretion pathway protein GspD	VT06_11460	Arsukibacterium sp. MJ3	690
A0A0N0DL14	General secretion pathway protein GspD	VT25_01490	Photobacterium leiognathi subsp. mandapamensis (Photobacterium mandapamensis)	677
A0A0N1EJL4	General secretion pathway protein GspD	ADS77_20355	Pseudoalteromonas porphyrae	686
A0A0N8TW84	Type II secretion system protein D	pulD AN392_03499	Pseudoalteromonas sp. P1-16-1b	687
A0A0P6Z6C1	General secretion pathway protein GspD	AN167_11180	Vibrio splendidus	674
A0A0P7DJK6	Type II secretion system protein GspD	AOG27_20435	Pseudoalteromonas lipolytica	687
A0A0P7XNR1	Type 2a secretion system secretin GspD	gspD HLUCCO02_07120	Idiomarinaceae bacterium HL-53	694
A0A0P9FTN3	Type II secretion system protein D	pulD_2 AN390_03838	Pseudoalteromonas sp. P1-11	689
A0A0P9I655	Type II secretion system protein D	pulD AN214_01337	Pseudoalteromonas sp. P1-9	689
A0A0Q2MF39	Type II secretion system protein GspD	AMR76_08725	Vibrio furnissii	675

A0A0S1YQF5	General secretion pathway protein D	AOR13_3659	Alteromonas stellipolaris LMG 21856	680
A0A0S2JJA4	Type II secretion system protein GspD	CMT41_16135	Colwellia sp. MT41	692
A0A0S2K6C1	General secretion pathway protein D	PP2015_3108	Pseudoalteromonas phenolica	687
A0A0T5PG51	General secretion pathway protein GspD	AAY72_15180	Alishewanella sp. WH16-1	699
A0A0U2JJS9	Type II secretion system protein GspD	AT746_18455	Lacimicrobium alkaliphilum	687
A0A0U2WIC4	General secretion pathway protein D	gspD PTRA_a0248	Pseudoalteromonas translucida KMM 520	688
A0A0W1KS66	Type II secretion system protein GspD	ATS75_19555	Pseudoalteromonas sp. H105	687
A0A0W1L0W2	Type II secretion system protein GspD	ATS76_18555	Pseudoalteromonas sp. 10-33	687
A0A0W1L249	Type II secretion system protein GspD	ATS74_19325	Pseudoalteromonas sp. H103	690
A0A0W1SUL9	Type II secretion system protein GspD	AUR68_10140	Idiomarina sp. H105	687
A0A0W8JAC9	General secretion pathway protein D (Cholera toxin secretion protein epsD)	VRK_39200	Vibrio sp. MEBiC08052	661
A0A109DBI8	Type II secretion system protein GspD	APQ14_00335	Vibrio toranzoniae	674
A0A109X3U7	Type II secretion system protein GspD	AL536_21270	Vibrio fluvialis	678
A0A124GPI8	Type II secretion system protein GspD	AR688_02050	Rheinheimera sp. EpRS3	706
A0A125WTP0	Type II secretion system protein D	AB97_5387	Escherichia coli 1-110-08_S3_C1	691
A0A135ZZ67	Type II secretion system protein GspD	AX660_18025	Paraglaciecola sp. S66	682
A0A136I109	Type II secretion system protein GspD	AXW14_09010	Alteromonas sp. Nap_26	666
A0A139C570	General secretion pathway protein D	AWU56_47	Idiomarina sp. T82-3	695
A0A142BHQ1	General secretion pathway protein D	gspD1 EZMO1_4360	Endozoicomonas montiporae CL-33	719
A0A151JIJ7	Type II secretion system protein GspD	AUQ44_09210	Vibrio cidicii	673
A0A161ZS35	General secretion pathway protein D	N475_24905	Pseudoalteromonas luteoviolacea DSM 6061	686
A0A165KUH7	Type II secretion system protein GspD	A1L58_10990	Shewanella baltica	704
A0A165SBZ7	Type II secretion system protein GspD	A3712_06560	Vibrio sp. HI00D65	678
A0A167F324	General secretion pathway protein D	N476_12160	Pseudoalteromonas luteoviolacea H33	687
A0A178J9B9	Type II secretion system protein GspD	AZ468_12640	Vibrio europaeus	675
A0A193K8F6	Type II secretion system protein GspD	A6E01_00710	Vibrio breoganii	676
A0A1A6KV05	Type II secretion system protein GspD	A9264_04090	Vibrio sp. UCD-FRSSP16_10	675
A0A1A6LA73	Type II secretion system protein GspD	A9262_20120	Vibrio splendidus	674
A0A1A6W3Q9	Type II secretion system protein GspD	AYY27_13850	Photobacterium damselae	668
A0A1A9AU38	Pullulanase secretion envelope pulD	pulD_1 SAMEA2665130_00201	Plesiomonas shigelloides (Aeromonas shigelloides)	684
A0A1A9AVM5	Pullulanase secretion envelope pulD	pulD_2 SAMEA2665130_00785	Plesiomonas shigelloides (Aeromonas shigelloides)	708
A0A1B1BR17	Type II secretion system protein GspD	A134_00955	Vibrio crassostreae 9CS106	674

A0A1B1E8H9	Type II secretion system protein GspD	BA894_00760	Vibrio natriegens	673
A0A1B1NK66	Type II secretion system protein	VSVS12_00289	Vibrio scophthalmi	674
A0A1B7UFI3	Type II secretion system protein GspD	A5320_19650	Rheinheimera sp. SA_1	693
A0A1B8I9K7	Type II secretion system protein GspD	AYY24_18760	Photobacterium phosphoreum	674
A0A1B9NV67	Type II secretion system protein GspD	A6E04_17200	Aliivibrio logei (Vibrio logei)	679
A0A1B9P569	Type II secretion system protein GspD	A6E13_10145	Aliivibrio fischeri (Vibrio fischeri)	678
A0A1B9PM84	Type II secretion system protein GspD	A6E06_16685	Aliivibrio sp. 1S175	677
A0A1B9PVJ7	Type II secretion system protein GspD	A6E08_07170	Vibrio lentus	674
A0A1B9QVD0	Type II secretion system protein GspD	A6E14_00625	Vibrio genomosp. F10	676
A0A1C3EJQ4	Type II secretion system protein GspD	A8L45_10445	Enterovibrio pacificus	665
A0A1C3IRV5	Type II secretion system protein D	pulD_2 VAT7223_02038	Vibrio atlanticus	674
A0A1C3IUT4	Type II secretion system protein D	pulD_1 VHE8714_02590	Vibrio hemicentroti	673
A0A1C7FDK5	Type II secretion system protein	VSVS05_02719	Vibrio scophthalmi	673
A0A1D2X6G0	Type II secretion system protein GspD	ACH42_15185	Endozoicomonas sp. (ex Bugula neritina AB1)	695
A0A1D8S131	Type II secretion system protein GspD	A3Q34_08370	Colwellia sp. PAMC 20917	690
A0A1E3WYI4	Type II secretion system protein GspD	BCD66_07960	Pseudoalteromonas tetraodonis	688
A0A1E5E947	Type II secretion system protein GspD	A163_01015	Vibrio tasmaniensis 1F-267	674
A0A1E5IW96	Type II secretion system protein GspD	BEL05_01955	Shewanella colwelliana (Alteromonas colwelliana)	706
A0A1E7D912	Type II secretion system protein D	gspD BFV94_4083	Alteromonas macleodii (Pseudoalteromonas macleodii)	683
A0A1E7Q245	Type II secretion system protein GspD	BI198_00430	Rheinheimera salexigens	710
A0A1E7ZDR6	Type II secretion system protein GspD	BFC18_07935	Alteromonas confluentis	677
A0A1E8FCD0	Type II secretion system protein GspD	BFC17_02210	Alteromonas lipolytica	681
A0A1H6ASV5	General secretion pathway protein D	SAMN04488244_11696	Vibrio hangzhouensis	657
A0A1I7CRT9	General secretion pathway protein D	SAMN04487854_12924	Pseudoalteromonas lipolytica	687
A0A1J0RZ46	Type II secretion system protein GspD	BM527_18400	Alteromonas sp. Mex14	665
A0A1J0TIV3	Type II secretion system protein GspD	BM528_17395	Alteromonas sp. RW2A1	681
A0A1L8MJP7	Type II secretion system protein GspD	BK026_13990	Alteromonas sp. V450	665
A0A1M5FAI0	General secretion pathway protein D	SAMN05216361_0751	Aestuariibacter aggregatus	686
A0A1M5FR01	General secretion pathway protein D	SAMN02745781_03542	Vibrio gazogenes DSM 21264	661
A0A1M5YUW5	Type II secretion system protein D	pulD_1 VA7868_02003	Vibrio aerogenes CECT 7868	676
A0A1M5Z8V4	General secretion pathway protein D	SAMN02745129_0021	Ferrimonas marina	713
A0A1M7Z098	Type II secretion system protein D	pulD_2 VQ7734_04129	Vibrio quintilis	676
A0A1N6M4Y3	Type II secretion system protein D	pulD VSP9026_02108	Vibrio sp. CECT 9026	663
A0A1N7A2Y5	Type II secretion system protein D (GspD)	SAMN05421840_11632	Shewanella morhuae	702

A0A1Q1PDN2	Type II secretion system protein D	pulD_1 Vca1114GL_01835	Vibrio campbellii	681
A0A1Q2GXQ5	Type II secretion system protein GspD	B0W48_08920	Pseudoalteromonas aliena	687
A0A1Q9HBH3	Type II secretion system protein GspD	BIY21_03770	Vibrio ponticus	671
A0A1R3EXV0	Type II secretion system protein GspD	BH581_02195	Vibrio splendidus	673
A0A1R4K015	General secretion pathway protein D	FM109_15115	Vibrio sp. JB196	671
A0A1R4LPW1	Type II secretion system protein D	pulD VR7878_02915	Vibrio ruber DSM 16370	677
A0A1S1MMU3	Type II secretion system protein GspD	BET10_01320	Pseudoalteromonas sp. JW1	690
A0A1S1NH45	Type II secretion system protein GspD	BIW53_00480	Pseudoalteromonas byunsanensis	688
A0A1S2CN32	Type II secretion system protein GspD	BI375_06715	Vibrio rotiferianus	674
A0A1S2SZ53	Type II secretion system protein GspD	BFR57_02235	Idiomarina sp. MD25a	695
A0A1S6HYF0	Type II secretion system protein D	Sps_05455	Shewanella psychrophila	721
A0A1S8F5Z8	Type II secretion system protein GspD	BZG77_10640	Salinivibrio sp. IB643	659
A0A1T4RTK5	General secretion pathway protein D	SAMN02745782_02813	Vibrio cincinnatiensis DSM 19608	679
A0A1T4U298	Type II secretion system protein D	pulD_2 CZ814_02671	Photobacterium sp. H01100410B	674
A0A1T5I4J2	Type II secretion system protein D	pulD_2 CZ809_03558	Photobacterium piscicola	672
A1S1X4	Type II and III secretion system protein	Sama_0169	Shewanella amazonensis (strain ATCC BAA-1098 / SB2B)	703
A3DAA8	General secretion pathway protein D	Sbal_4206	Shewanella baltica (strain OS155 / ATCC BAA-1091)	705
A3QJ37	General secretion pathway protein D	Shew_3619	Shewanella loihica (strain ATCC BAA-1088 / PV-4)	707
A5KYR3	Type II secretory pathway, component EpsD	VSWAT3_11126	Vibrionales bacterium (strain SWAT-3)	674
A8G1I7	General secretion pathway protein D	Ssed_4358	Shewanella sediminis (strain HAW-EB3)	724
A8GYT6	General secretion pathway protein D	Spea_0144	Shewanella pealeana (strain ATCC 700345 / ANG-SQ1)	710
A9D5J9	General secretion pathway protein D	KT99_02231	Shewanella benthica KT99	711
B0TMX6	General secretion pathway protein D	Shal_4174	Shewanella halifaxensis (strain HAW-EB4)	720
B1KN27	General secretion pathway protein D	Swoo_4734	Shewanella woodyi (strain ATCC 51908 / MS32)	714
B8CH36	Type II and III secretion system protein:NolW-like protein	swp_0186	Shewanella piezotolerans (strain WP3 / JCM 13877)	721
B8E3T2	General secretion pathway protein D	Sbal223_0153	Shewanella baltica (strain OS223)	706
C4K366	Uncharacterized protein	gspD HDEF_0241	Hamiltonella defensa subsp. Acyrthosiphon pisum (strain 5AT)	687
C9P1Q9	General secretion pathway protein D (Cholera toxin secretion protein epsD)	VIB_000407	Vibrio metschnikovii CIP 69.14	663
D0I313	Type II secretory pathway component EpsD	VHA_000144	Grimontia hollisae CIP 101886	671
D0IED4	Type II secretory pathway component PulD	VOA_003575	Vibrio sp. RC586	675
D0Z0Q8	Putative type II secretory pathway component EpsD	VDA_003122	Photobacterium damselae subsp. damselae CIP 102761	579

D4ZEB1	General secretion pathway protein D	gspD SVI_4201	Shewanella violacea (strain JCM 10179 / CIP 106290 / LMG 19151 / DSS12)	712
E1SL34	General secretion pathway protein D	Fbal_0214	Ferrimonas balearica (strain DSM 9799 / CCM 4581 / PAT)	723
E3BIX5	General secretion pathway protein D	VIBC2010_19825	Vibrio caribbeanicus ATCC BAA-2122	674
E6XGB2	General secretion pathway protein D	Sput200_0391	Shewanella putrefaciens (strain 200)	705
E8LY79	General secretion pathway protein D	VIBR0546_13372	Vibrio brasiliensis LMG 20546	674
E8M4Z5	General secretion pathway protein D	VISI1226_16443	Vibrio sinaloensis DSM 21326	675
F2P4Z0	General secretion pathway protein D	gspD PMSV_4049	Photobacterium leiognathi subsp. mandapamensis svers.1.1.	677
F4AHI3	General secretion pathway protein D	Glaag_0143	Glaciecola sp. (strain 4H-3-7+YE-5)	690
F7NYC9	General secretion pathway protein D	Rhein_2812	Rheinheimera sp. A13L	696
F7RID7	General secretion pathway protein D	SOHN41_00101	Shewanella sp. HN-41	685
F7S076	General secretion pathway protein D	A28LD_1738	Idiomarina sp. A28L	696
F9S5E3	General secretion pathway protein D	VII00023_14453	Vibrio ichthyoenteri ATCC 700023	675
F9SJD9	General secretion pathway protein D	VISP3789_20253	Vibrio splendidus ATCC 33789	674
F9SQD3	General secretion pathway protein D	VIOR3934_06794	Vibrio orientalis CIP 102891 = ATCC 33934	675
G4QNE2	General secretion pathway protein D	gspD GNIT_3467	Glaciecola nitratireducens (strain JCM 12485 / KCTC 12276 / FR1064)	690
H1ADG7	General secretion pathway protein D	gspD	Shewanella livingstonensis	710
H3ZF42	General secretion pathway protein D	AJE_09884	Alishewanella jeotgali KCTC 22429	697
H5TAS9	General secretion pathway protein D	pulD GPUN_1282	Glaciecola punicea ACAM 611	684
I1E159	General secretion pathway protein D	epsD RNAN_3050	Rheinheimera nanhaiensis E407-8	706
I9DQN1	General secretion pathway protein D	AGRI_11787	Alishewanella agri BL06	698
K2KJQ9	General secretion pathway protein D	B3C1_02050	Gallaecimonas xiamenensis 3-C-1	696
K2L638	General secretion pathway protein D	A10D4_02710	Idiomarina xiamenensis 10-D-4	709
K6Y484	General secretion pathway protein D	pulD GPAL_0724	Glaciecola pallidula DSM 14239 = ACAM 615	692
K6YIC2	General secretion pathway protein D	pulD GARC_0936	Paraglaciecola arctica BSs20135	691
K6YJH8	General secretion pathway protein D	pulD GMES_1855	Paraglaciecola mesophila KMM 241	689
K6YYR1	General secretion pathway protein D	pulD GLIP_3729	Aliiglaciecola lipolytica E3	682
K7AHG1	General secretion pathway protein D	C427_5484	Paraglaciecola psychrophila 170	685
K7AJC8	General secretion pathway protein D	pulD GPLA_4431	Paraglaciecola polaris LMG 21857	690
L8DF70	General secretion pathway protein D	PALB_36930	Pseudoalteromonas luteoviolacea B = ATCC 29581	690
L8J363	General secretion pathway protein D	C942_03972	Photobacterium marinum	669
L8XMA0	General secretion pathway protein D	B878_02641	Vibrio campbellii CAIM 519 = NBRC 15631	676
N6VWF3	General secretion pathway protein D	J139_14025	Pseudoalteromonas agarivorans S816	690

Q0I0E8	General secretion pathway protein D	Shewmr7_0151	Shewanella sp. (strain MR-7)	700
Q12T08	Type II and III secretion system protein	Sden_0121	Shewanella denitrificans (strain OS217 / ATCC BAA-1090 / DSM 15013)	705
Q1YWZ3	Putative type II secretory pathway, component EpsD	P3TCK_23583	Photobacterium profundum 3TCK	670
Q1ZL65	Putative type II secretory pathway, component EpsD	VAS14_23049	Photobacterium angustum (strain S14 / CCUG 15956) (Vibrio sp. (strain S14 / CCUG 15956))	674
Q2C2L5	Putative type II secretory pathway, component EpsD	SKA34_00710	Photobacterium sp. SKA34	672
Q47VD4	General secretion pathway protein D	gspD CPS_4591	Colwellia psychrerythraea (strain 34H / ATCC BAA-681) (Vibrio psychroerythus)	697
Q5E1X7	General secretion pathway protein D	gspD VF_2474	Vibrio fischeri (strain ATCC 700601 / ES114)	673
Q5QYA4	Type II secretory pathway, component PulD	pulD IL2026	Idiomarina loihiensis (strain ATCC BAA-735 / DSM 15497 / L2-TR)	708
Q6LLS6	Putative type II secretory pathway, component EpsD	SO0166 PBPRA3481	Photobacterium profundum (strain SS9)	670
Q8RTI3	General secretion pathway protein D (WmpD)	PTD2_16766	Pseudoalteromonas tunicata D2	689
S3DZY3	General secretion pathway protein D	gspD O1U_0831	Candidatus Photodesmus katoptron Akat1	669
T8K999	Type II secretion system protein D	G893_02121	Escherichia coli KOEGE 71 (186a)	685
U1J8E4	General secretion pathway protein D	PCIT_15960	Pseudoalteromonas citrea DSM 8771	689
U1L8B2	General secretion pathway protein D	PSPO_03035	Pseudoalteromonas spongiae UST010723-006	688
U3AQV9	Type II secretion system protein D	gspD VAZ01S_028_00210	Vibrio azureus NBRC 104587	681
U3BA40	Type II secretion system protein D	gspD VPR01S_04_02930	Vibrio proteolyticus NBRC 13287	676
U3CQY9	Putative general secretory pathway protein D	VEZ01S_37_00780	Vibrio ezurae NBRC 102218	674
U4E0L7	General secretion pathway protein D	epsD VIBNIFTn2_1640060	Vibrio nigripulchritudo FTn2	677
V4HQ07	General secretion pathway protein D	PL2TA16_05201	Pseudoalteromonas luteoviolacea 2ta16	686
V5F1V4	General secretion pathway protein D	PLEI_0392	Photobacterium leiognathi lrivu.4.1	676
V5HED6	Type II secretion system protein D	gspD VHA01S_003_00110	Vibrio halioticoli NBRC 102217	675
W7R0U8	General secretion pathway protein D	DS2_05215	Catenovulum agarivorans DS-2	730

Burkholderiales and *Pseudomonas aeruginosa/fluorescens/syringae* HxcQ

Entry	Protein names	Gene names	Organism	Length
Q9I5P0	Probable type II secretion system protein	PA0685	Pseudomonas aeruginosa (strain ATCC 15692 / DSM 22644 / CIP 104116 / JCM 14847 / LMG 12228 / 1C / PRS 101 / PAO1)	803
A0A0M7MBB9	Pullulanase secretion envelope pulD	pulD_4 ERS370003_06267	Achromobacter sp.	489
A0A1R1JLJ6	Type II secretion system protein GspD (Fragment)	BIZ92_32945	Alcaligenes xylosoxydans xylosoxydans (Achromobacter xylosoxidans)	515
A0A0Q6N2T0	Type II secretion system protein GspD (Fragment)	ASC78_06040	Variovorax sp. Root318D1	681
A0A0S8DFZ5	Uncharacterized protein	AMJ66_02010	Betaproteobacteria bacterium SG8_40	675
A0A0S9L735	Uncharacterized protein	ASF45_33390	Pseudorhodoferax sp. Leaf265	639
A0A1P8JUK3	Uncharacterized protein	RD110_09800	Rhodoferax sp. DCY110	643
A0A0J6KAN3	General secretion pathway protein GspD	VI26_09895	Chromobacterium sp. LK1	715
A0A0J6NM59	General secretion pathway protein GspD	VK98_11730	Chromobacterium sp. LK11	717
A0A1D9LG72	Type II secretion system protein GspD	BKX93_09895	Chromobacterium vaccinii	715
A0A1R0MVB3	Type II secretion system protein GspD	BRL65_03115	Chromobacterium aquaticum	708
A0A1S1X005	Type II secretion system protein GspD	BI347_04600	Chromobacterium sp. 37-2	711
A0A1S1XB59	Type II secretion system protein GspD	BI343_13320	Chromobacterium amazonense	704
U0Z619	Uncharacterized protein	O166_15450	Pseudogulbenkiania ferrooxidans EGD-HP2	710
A0A0X8GLZ7	Type II secretion system protein GspD	VN23_01170	Janthinobacterium sp. B9-8	680
A0A142LN84	General secretion pathway protein GspD	AEM42_07065	Betaproteobacteria bacterium UKL13-2	656
A0A1B4V3U1	General secretion pathway protein D	SVA_1640	Sulfurifustis variabilis	735
A0A1B4XGF5	General secretion pathway protein D	SCL_1554	Sulfuricaulis limicola	767
A0A1D9B990	Type II secretion system protein GspD	BJP62_00085	Jeongeupia sp. USM3	702
A0A1E4HUE1	Type II secretion system protein GspD (Fragment)	ABS89_08995	Thiobacillus sp. SCN 63-1177	806
A0A1F6TQ20	Type II secretion system protein GspD	A2151_00775	Candidatus Muproteobacteria bacterium RBG_16_65_34	667
A0A1F6U4I9	Type II secretion system protein GspD (Fragment)	A3A87_02470	Candidatus Muproteobacteria bacterium RIFCSPLOWO2_01_FULL_60_18	614
A0A1J4Z1X6	Type II secretion system protein GspD	AUJ86_07855	Hydrogenophilaceae bacterium CG1_02_62_390	678
A0A1Q4GS46	Type II secretion system protein GspD	BGP21_03810	Thiobacillus sp. 65-29	652
N6YLK7	General secretion pathway protein D	B447_10323	Thauera sp. 27	675
A0A016XGX7	General secretion pathway protein GspD	AZ34_08210	Hylemonella gracilis str. Niagara R	738

A0A031GRN4	General secretion pathway protein D	gspD_2 BW37_02982	Janthinobacterium lividum	748
A0A038H622	General secretion pathway protein GspD	BG58_30265	Caballeronia jiangsuensis	800
A0A059KHY7	General secretion pathway protein D	X805_33680	Sphaerotilus natans subsp. natans DSM 6575	735
A0A060NFK6	Type II secretory pathway, component PulD	SRAA_0387	Comamonadaceae bacterium A1	742
A0A068YUZ5	General secretion pathway protein D		Polaromonas sp. CG9_12	732
A0A076PDH7	General secretion pathway protein D	O987_03425	Comamonas testosteroni TK102	793
A0A078BL83	Putative general secretion pathway protein	THICB2_430030	Thiomonas sp. CB2	888
A0A085FY45	General secretion pathway protein D	FG94_00207	Massilia sp. LC238	786
A0A096C4Z4	General secretion pathway protein D	P368_14040	Comamonas testosteroni (Pseudomonas testosteroni)	793
A0A096H317	General secretion pathway protein D (General secretion pathway protein GspD)	GL58_13945 P353_00905	Comamonas testosteroni (Pseudomonas testosteroni)	792
A0A098UCP7	General secretion pathway protein GspD	IA69_07510	Massilia sp. JS1662	759
A0A0A1FLJ5	General secretion pathway protein D	LT85_4604	Collimonas arenae	780
A0A0A1VC07	Type II secretory pathway, component PulD (Fragment)	AVS7_00425	Acidovorax sp. MR-S7	722
A0A0D0MGL2	General secretion pathway protein GspD	RT97_18365	Variovorax paradoxus	770
A0A0D7K9L6	General secretion pathway protein GspD	RP29_14290	Acidovorax temperans	834
A0A0E9MB58	General secretion pathway protein GspD	SCT_0020	Sulfuricella sp. T08	667
A0A0G3BPX8	General secretion pathway protein GspD	gspD AAW51_4793	[Polyangium] brachysporum	774
A0A0H2M6F2	Type II secretion system protein D	xcpQ VPARA_12240	Variovorax paradoxus	772
A0A0J0US83	General secretion pathway protein GspD	OX89_00295	Diaphorobacter sp. J5-51	781
A0A0K6HWP9	Type II secretion system protein D (GspD)	Ga0061069_103111	Thiomonas bhubaneswarensis	876
A0A0K8NVX8	General secretion pathway protein D	ISF6_4727	Ideonella sakaiensis (strain 201-F6)	792
A0A0L6TG09	General secretion pathway protein GspD	AD742_01690	Methylibium sp. NZG	735
A0A0L6VVR5	General secretion pathway protein GspD	AB595_26945	Massilia sp. WF1	779
A0A0L8B1W7	General secretion pathway protein GspD	H663_00115	Limnohabitans planktonicus II-D5	708
A0A0N0JE75	Type II secretion system protein GspD	IP80_04600	beta proteobacterium AAP65	715
A0A0N1ATF0	Type II secretion system protein GspD	D621_07800	beta proteobacterium AAP51	726
A0A0N1LDD5	Uncharacterized protein	IP84_04555	beta proteobacterium AAP99	698
A0A0N8P5R1	Type II secretion system protein GspD	APR48_43505 APR50_07035 APR52_30375	Variovorax paradoxus	776
A0A0P0M763	Type II secretion system protein D	xcpQ L63ED372_00255	Limnohabitans sp. 63ED37-2	734
A0A0P0MD68	Type II secretion system protein D	xcpQ L103DPR2_00652	Limnohabitans sp. 103DPR2	716
A0A0Q0Y4M8	General secretion pathway protein GspD	AE621_16820	Acidovorax sp. SD340	870

A0A0Q4RIG4	Type II secretion system protein GspD	ASF16_17465	Acidovorax sp. Leaf78	821
A0A0Q4YM57	Type II secretion system protein GspD	ASF43_05280	Pseudorhodoferax sp. Leaf267	800
A0A0Q5H5L9	Type II secretion system protein GspD	ASF77_15980	Massilia sp. Leaf139	743
A0A0Q5MKK4	Type II secretion system protein GspD	ASF94_01800	Acidovorax sp. Leaf160	805
A0A0Q5ZI59	Type II secretion system protein GspD	ASG30_00160	Ramlibacter sp. Leaf400	734
A0A0Q6LX62	Type II secretion system protein GspD	ASC88_08475	Rhizobacter sp. Root29	740
A0A0Q6T1M0	Type II secretion system protein GspD	ASC93_15875	Massilia sp. Root335	784
A0A0Q6U3T5	Type II secretion system protein GspD	ASC95_06565	Pelomonas sp. Root1217	746
A0A0Q6WIR7	Type II secretion system protein GspD	ASC91_20275	Pelomonas sp. Root1237	751
A0A0Q6XTD0	Type II secretion system protein GspD	ASC87_13000	Rhizobacter sp. Root1221	745
A0A0Q6ZFA8	Type II secretion system protein GspD	ASC83_02950	Acidovorax sp. Root402	758
A0A0Q7AU18	Type II secretion system protein GspD	ASC76_01015	Rhizobacter sp. Root404	751
A0A0Q7BVX5	Type II secretion system protein GspD	ASC81_03360	Pelomonas sp. Root405	744
A0A0Q7CHG9	Type II secretion system protein GspD	ASC92_00815	Variovorax sp. Root411	764
A0A0Q7DDE8	Type II secretion system protein GspD	ASC67_04185	Methylibium sp. Root1272	732
A0A0Q7ELA8	Type II secretion system protein GspD	ASC94_21340	Massilia sp. Root418	733
A0A0Q7LIA3	Type II secretion system protein GspD	ASD34_23370	Variovorax sp. Root473	778
A0A0Q7SZY5	Type II secretion system protein GspD	ASD35_16275	Pelomonas sp. Root1444	752
A0A0Q7W004	Type II secretion system protein GspD	ASD58_17480	Duganella sp. Root1480D1	735
A0A0Q7WYP0	Type II secretion system protein GspD	ASD92_03800	Massilia sp. Root1485	757
A0A0Q8L0L3	Type II secretion system protein GspD	ASD94_11595	Acidovorax sp. Root70	760
A0A0Q8RII7	Type II secretion system protein GspD	ASE26_18980	Duganella sp. Root198D2	735
A0A0Q8TBJ7	Type II secretion system protein GspD	ASE31_15235	Acidovorax sp. Root217	825
A0A0Q8TLD5	Type II secretion system protein GspD	ASE28_14555	Acidovorax sp. Root219	823
A0A0Q9CUE9	Type II secretion system protein GspD	ASE52_21350	Acidovorax sp. Root275	821
A0A0S4P2X8	General secretion pathway protein D	BN2497_8051 BN3177_8051	Janthinobacterium sp. CG23_2	750
A0A0S9EVS9	Type II secretion system protein GspD	ASF11_05255	Acidovorax sp. Leaf76	834
A0A0S9KSD7	Type II secretion system protein GspD	ASF45_16320	Pseudorhodoferax sp. Leaf265	721
A0A0S9NTH2	Type II secretion system protein GspD	ASF44_22100	Pseudorhodoferax sp. Leaf274	791
A0A0T2ZC47	Type II secretion system protein GspD	ASE11_22665	Hydrogenophaga sp. Root209	707
A0A0U3NA82	General secretion pathway protein D	RD2015_944	Roseateles depolymerans	755
A0A109D2B7	General secretion pathway protein D	APY03_1550	Variovorax sp. WDL1	760
A0A126ZA14	Type II secretion system protein GspD	AX767_05955	Variovorax sp. PAMC 28711	759
A0A127PIR1	Type II secretion system protein D	gspD CFter6_4940	Collimonas fungivorans	760

A0A127QBV6	Type II secretion system protein D	gspD CPter91_5037	Collimonas pratensis	765
A0A139BV22	General secretion pathway protein GspD	AWT59_1043	Candidatus Gallionella acididurans	814
A0A147GN38	General secretion pathway protein GspD	NS331_21075	Pseudacidovorax intermedius	772
A0A165KP54	Type II secretion system protein GspD	A1D30_09315	Acidovorax sp. GW101-3H11	748
A0A167HPQ8	Type II secretion system protein GspD	LPB072_03010	Hydrogenophaga sp. LPB0072	682
A0A1A6DU88	Type II secretion system protein GspD	A9O67_04095	Tepidimonas fonticaldi	712
A0A1A7BUS8	General secretion pathway protein D	ASR47_1002314	Janthinobacterium sp. S3-2	756
A0A1B3M4I6	Type II secretion system protein D	gspD BSY239_3156	Hydrogenophaga sp. RAC07	726
A0A1B3PGW3	Type II secretion system protein D	gspD BSY15_2167	Acidovorax sp. RAC01	882
A0A1C6PLG7	General secretion pathway protein D	VAR608DRAFT_4271	Variovorax sp. HW608	755
A0A1C9VAP8	Type II secretion system protein GspD	Q5W_01945	Hydrogenophaga sp. PBC	742
A0A1D2S1V8	Type II secretion system protein GspD	ABS38_01950	Acidovorax sp. SCN 68-22	755
A0A1D2U5S9	Type II secretion system protein GspD	ABT03_09555	Comamonas sp. SCN 67-35	773
A0A1D8J7I6	General secretion pathway protein D (Type II secretion system protein GspD)	BI380_26565 SAMN05444579_102194	Delftia tsuruhatensis	789
A0A1E3YXW8	Type II secretion system protein GspD	ABS37_04930	Acidovorax sp. SCN 65-108	830
A0A1E4IIM3	Type II secretion system protein GspD	ABS84_08465	Rubrivivax sp. SCN 71-131	740
A0A1E4IYR1	Type II secretion system protein GspD	ABS94_18675	Variovorax sp. SCN 67-85	763
A0A1E4LUG6	Type II secretion system protein GspD	ABT02_08810	Comamonadaceae bacterium SCN 68-20	784
A0A1E7U6F3	Type II secretion system protein GspD	AO062_05430	Variovorax boronicumulans	777
A0A1F3WXU9	Type II secretion system protein GspD	A2V79_02285	Betaproteobacteria bacterium RBG_16_56_24	727
A0A1F3ZY75	Type II secretion system protein GspD	A3H35_10480	Betaproteobacteria bacterium RIFCSPLOWO2_02_FULL_62_17	640
A0A1F4B424	Type II secretion system protein GspD	A3H32_01095	Betaproteobacteria bacterium RIFCSPLOWO2_02_FULL_63_19	711
A0A1F4CGZ0	Type II secretion system protein GspD	A3F75_06910	Betaproteobacteria bacterium RIFCSPLOWO2_12_FULL_64_23	673
A0A1F4CLZ1	Type II secretion system protein GspD	A3G26_04480	Betaproteobacteria bacterium RIFCSPLOWO2_12_FULL_65_110	714
A0A1F4CQD8	Type II secretion system protein GspD	A3G28_06220	Betaproteobacteria bacterium RIFCSPLOWO2_12_FULL_68_19	686
A0A1F4EC50	Type II secretion system protein GspD	A2710_27060	Burkholderiales bacterium RIFCSPHIGHO2_01_FULL_64_960	829
A0A1F4G7N6	Type II secretion system protein GspD	A2711_05015	Burkholderiales bacterium RIFCSPHIGHO2_01_FULL_63_240	756
A0A1F4GZQ5	Type II secretion system protein GspD	A2X72_13865	Burkholderiales bacterium GWF1_66_17	697

A0A1F4HNX9	Type II secretion system protein GspD	A3E25_04130	Burkholderiales bacterium RIFCSPHIGHO2_12_FULL_69_20	713
A0A1F4J737	Type II secretion system protein GspD	A3I64_04120	Burkholderiales bacterium RIFCSPLOWO2_02_FULL_67_64	727
A0A1F4JZD9	Type II secretion system protein GspD	A3I66_07360	Burkholderiales bacterium RIFCSPLOWO2_02_FULL_57_36	735
A0A1F4KE56	Type II secretion system protein GspD	A3F78_21805	Burkholderiales bacterium RIFCSPLOWO2_12_FULL_61_40	714
A0A1F4LLF4	Type II secretion system protein GspD	A3F76_10685	Burkholderiales bacterium RIFCSPLOWO2_12_FULL_65_40	687
A0A1F4M4D7	Type II secretion system protein GspD	A2503_08875	Burkholderiales bacterium RIFOXYD12_FULL_59_19	695
A0A1F4MQS2	Type II secretion system protein GspD	A3G29_11510	Burkholderiales bacterium RIFCSPLOWO2_12_FULL_64_99	758
A0A1F4NIW2	Type II secretion system protein GspD	A2486_10390	Burkholderiales bacterium RIFOXYC12_FULL_65_23	713
A0A1F8VIM4	Type II secretion system protein GspD	A2037_12920	Curvibacter sp. GWA2_63_95	701
A0A1G0D0C4	Type II secretion system protein GspD	A3H31_07465	Gallionellales bacterium RIFCSPLOWO2_02_FULL_57_47	717
A0A1G0DKQ5	Type II secretion system protein GspD	A2Z65_05090	Gallionellales bacterium RIFCSPLOWO2_02_58_13	712
A0A1G0ETT8	Type II secretion system protein GspD	A3J49_19275	Gallionellales bacterium RIFCSPHIGHO2_02_FULL_57_16	721
A0A1G0EUN3	Type II secretion system protein GspD	A3J49_11980	Gallionellales bacterium RIFCSPHIGHO2_02_FULL_57_16	746
A0A1H5SRF5	General secretion pathway protein D	SAMN03159365_00225	Variovorax sp. NFACC29	779
A0A1I7JC14	General secretion pathway protein D	SAMN04489707_102441	Acidovorax caeni	789
A0A1I9Y3K2	General secretion pathway protein GspD	YQ44_25440	Janthinobacterium sp. 1_2014MBL_MicDiv	747
A0A1J4RWC7	Type II secretion system protein GspD	AUJ20_11290	Comamonadaceae bacterium CG1_02_60_18	709
A0A1J5BIZ6	Type II secretion system protein GspD	AUK53_09215	Betaproteobacteria bacterium CG2_30_59_46	676
A0A1J5BZ98	Type II secretion system protein GspD	AUK51_09995	Comamonadaceae bacterium CG2_30_59_20	694
A0A1J5C870	Type II secretion system protein GspD	AUK50_04105	Comamonadaceae bacterium CG2_30_57_122	701
A0A1J5CNR8	Type II secretion system protein GspD	AUK52_10590	Comamonadaceae bacterium CG2_30_60_41	707
A0A1K2HBQ0	General secretion pathway protein D	SAMN02745887_01054	Chitinimonas taiwanensis DSM 18899	683
A0A1M3C261	Type II secretion system protein GspD	BGO13_05385	Burkholderiales bacterium 66-5	771
A0A1M3JM63	Type II secretion system protein GspD	BGO74_02855	Burkholderiales bacterium 68-12	736
A0A1M5E017	General secretion pathway protein D	SAMN02745117_02496	Lampropedia hyalina DSM 16112	817
A0A1M5SN47	Type II secretion system protein D (GspD)	SAMN05428948_3829	Massilia sp. CF038	734

A0A1M7FCS3	General secretion pathway protein D	SAMN02787076_00345	Rhizobacter sp. OV335	741
A0A1M7TBN5	Type II secretion system protein D (GspD)	SAMN05192549_112122	Duganella sacchari	738
A0A1N6P313	Type II secretion system protein D (GspD)	SAMN05880566_101351	Janthinobacterium sp. TND4EL3	743
A0A1N6YYT4	General secretion pathway protein D	SAMN05880557_108237	Pseudacidovorax sp. RU35E	778
A0A1P8JSS9	Type II secretion system protein GspD	RD110_06035	Rhodoferax sp. DCY110	761
A0A1Q3R1Z2	Type II secretion system protein GspD	BGO36_14775	Burkholderiales bacterium 68-10	777
A0A1Q3RMQ4	Type II secretion system protein GspD	BGO22_03215	Hydrogenophaga sp. 70-12	742
A0A1Q3X682	Type II secretion system protein GspD	BGO62_10155	Thiobacillus sp. 65-1402	687
A0A1Q3XNU1	Type II secretion system protein GspD	BGO66_00860	Alicycliphilus sp. 69-12	778
A0A1Q4GEU6	Type II secretion system protein GspD	BGP20_14045	Thiobacillus sp. 63-78	679
A0A1S1U3C7	General secretion pathway protein GspD	AKG95_26540	Janthinobacterium lividum	745
A0A1S8FZC7	Type II secretion system protein GspD	B0E49_01110	Polaromonas sp. C04	780
A0A1S9A9S8	Type II secretion system protein GspD	B0920_22605	Massilia sp. KIM	773
A1TKM7	General secretion pathway protein D	Aave_0917	Acidovorax citrulli (strain AAC00-1) (Acidovorax avenae subsp. citrulli)	810
A1VK97	General secretion pathway protein D	Pnap_0756	Polaromonas naphthalenivorans (strain CJ2)	738
A1WNP1	General secretion pathway protein D	Veis_3529	Verminephrobacter eiseniae (strain EF01-2)	740
A2SKK0	General secretion pathway protein D	gspD Mpe_A3136	Methylibium petroleiphilum (strain ATCC BAA-1232 / LMG 22953 / PM1)	736
A9BXP9	General secretion pathway protein D	Daci_5697	Delftia acidovorans (strain DSM 14801 / SPH-1)	787
B1Y329	General secretion pathway protein D	Lcho_3427	Leptothrix cholodnii (strain ATCC 51168 / LMG 8142 / SP-6) (Leptothrix discophora (strain SP-6))	753
C4K9Q5	General secretion pathway protein D	Tmz1t_2529	Thauera sp. (strain MZ1T)	728
C5CPK3	General secretion pathway protein D	Vapar_1073	Variovorax paradoxus (strain S110)	774
C9YDY9	Putative uncharacterized protein	Csp_D27950	Curvibacter putative symbiont of Hydra magnipapillata	711
E6UWS7	General secretion pathway protein D	Varpa_1156	Variovorax paradoxus (strain EPS)	770
E8TZL0	General secretion pathway protein D	Alide_0792	Alicycliphilus denitrificans (strain DSM 18852 / JCM 14587 / BC)	777
F0Q9I5	General secretion pathway protein D	Acav_0869	Acidovorax avenae (strain ATCC 19860 / DSM 7227 / JCM 20985 / NCPPB 1011)	804
F1VU14	General secretion pathway protein D / Type II secretion outermembrane pore forming protein (PulD)	IMCC9480_3845	Oxalobacteraceae bacterium IMCC9480	696
F3KWC1	Type II and III secretion system protein	HGR_13794	Hylemonella gracilis ATCC 19624	740

F5Y0R0	Candidate secretin, general secretion pathway protein D, component of type II secretion system	gspD Rta_35410	Ramlibacter tataouinensis (strain ATCC BAA-407 / DSM 14655 / LMG 21543 / TTB310)	735
G0AE23	General secretion pathway protein D	gspD CFU_4050	Collimonas fungivorans (strain Ter331)	764
H0BZT1	General secretion pathway protein D	KYG_14588	Acidovorax sp. NO-1	725
H5WHD3	General secretion pathway protein D	BurJ1DRAFT_1122	Burkholderiales bacterium JOSHI_001	738
I0HMA1	Putative general secretion pathway protein D GspD	gspD RGE_07950	Rubrivivax gelatinosus (strain NBRC 100245 / IL144)	731
J1EGV9	General secretion pathway protein D (Fragment)	PMI14_03804	Acidovorax sp. CF316	800
K9DGT6	General secretion pathway protein D	HMPREF9710_00577	Massilia timonae CCUG 45783	804
N6XFJ0	General secretory pathway protein D	C664_02025	Thauera sp. 63	711
N6YRW5	General secretion pathway protein D	C666_15955	Thauera linaloolentis 47Lol = DSM 12138	745
N6ZPF9	General secretion pathway protein D	C667_14172	Thauera phenylacetica B4P	732
Q12DW3	Type II and III secretion system protein	Bpro_1329	Polaromonas sp. (strain JS666 / ATCC BAA-500)	747
Q221L0	Type II and III secretion system protein	Rfer_0542	Rhodoferax ferrireducens (strain ATCC BAA-621 / DSM 15236 / T118) (Albidiferax ferrireducens)	702
S6AGJ8	Type II and III secretion system protein	SCD_n01314	Sulfuricella denitrificans skB26	667
U5NC55	General secretion pathway protein D	gspD-2 Cenrod_1684	Candidatus Symbiobacter mobilis CR	701
W0VA07	General secretion pathway protein D	gspD GJA_5055	Janthinobacterium agaricidamnosum NBRC 102515 = DSM 9628	742
A0A023CCS3	General secretion pathway protein GspD	PE143B_0110075	Pseudomonas extremaustralis 14-3 substr. 14-3b	771
A0A031G894	Type II and III secretion system protein	BW33_01174	Pseudomonas sp. RIT288	793
A0A031J400	Putative type II secretion pathway protein D	BW43_01373	Pseudomonas sp. RIT357	768
A0A031MJH1	General secretion pathway protein GspD	CF98_07360	Pseudomonas bauzanensis	868
A0A064BPJ6	General secretion pathway protein GspD	DF40_003795	Stenotrophomonas maltophilia M30	793
A0A077LCS6	General secretion pathway protein D	PSCI_1513	Pseudomonas sp. StFLB209	784
A0A095UUP1	General secretion pathway protein GspD	JL37_17520	Achromobacter sp. RTa	870
A0A0A1HSP3	General secretion pathway protein D	BN844_2055	Pseudomonas sp. SHC52	784
A0A0A2N441	General secretion pathway protein GspD	JT27_14115	Alcaligenes faecalis	770
A0A0A6D540	General secretion pathway protein GspD	NZ35_25905	Pseudomonas chlororaphis	791
A0A0A6D7X6	General secretion pathway protein GspD	NZ35_18305	Pseudomonas chlororaphis	800
A0A0A8RHX5	Putative type II secretion system protein	PAMH19_3901	Pseudomonas aeruginosa	803
A0A0B7DAW3	Type II secretion system protein D	xcpQ SRM1_03003	Pseudomonas fluorescens	784
A0A0C1ZYI6	General secretion pathway protein GspD	QS95_14380	Pseudomonas fluorescens	783
A0A0C2RK13	General secretion pathway protein GspD	OC71_00095	Pseudomonas sp. W15Feb9B	793

A0A0C5E8S6	Uncharacterized protein	TO66_14090	Pseudomonas sp. MRSN12121	804
A0A0D0JPA7	General secretion pathway protein GspD	RU10_01900	Pseudomonas fluorescens	789
A0A0D1M3D8	General secretion pathway protein GspD	QV12_04285	Pseudomonas putida (Arthrobacter siderocapsulatus)	778
A0A0D6BJ81	General secretion pathway protein D	POS17_3296	Pseudomonas sp. Os17	795
A0A0D6HCN3	Pullulanase secretion envelope pulD	pulD_2 ERS451415_02362	Alcaligenes xylosoxydans xylosoxydans (Achromobacter xylosoxidans)	870
A0A0D6SZ03	General secretion pathway protein D	SZ55_1280	Pseudomonas sp. FeS53a	779
A0A0D9AZM5	General secretion pathway protein GspD	UG46_13610	Pseudomonas fluorescens	785
A0A0E1E3K7	General secretion pathway protein GspD	EY04_13335	Pseudomonas chlororaphis	804
A0A0F4XS74	General secretion pathway protein GspD	VP02_06125	Pseudomonas kilonensis	786
A0A0F7Y9F6	General secretion pathway protein D	CCOS191_3101	Pseudomonas sp. CCOS 191	788
A0A0G3GFB5	General secretion pathway protein GspD	VM99_17655	Pseudomonas chlororaphis	788
A0A0G6CGZ2	General secretion pathway protein D	xcpQ_3 PAERUG_E15_London_28_01_14_09845	Pseudomonas aeruginosa	793
A0A0H4VYT7	Type II secretion system protein D	xcpQ ACR54_00398	Bordetella hinzii	863
A0A0H5A2J7	General secretion pathway protein GspD	AA957_02715	Pseudomonas trivialis	773
A0A0J8FUA2	General secretion pathway protein GspD	ACR52_28075	Pseudomonas sp. KG01	777
A0A0K2IKX0	General secretion pathway protein GspD	YH67_11380	Stenotrophomonas maltophilia (Pseudomonas maltophilia) (Xanthomonas maltophilia)	793
A0A0L0R078	General secretion pathway protein GspD	AFM18_25725	Achromobacter spanius	801
A0A0L6FQZ5	General secretion pathway protein GspD	DA83_01980	Pseudomonas sp. 250J	789
A0A0L6IAY7	General secretion pathway protein GspD	AKG08_12445	Achromobacter piechaudii	791
A0A0M1EYN9	General secretion pathway protein GspD	ABW44_02845	Stenotrophomonas maltophilia (Pseudomonas maltophilia) (Xanthomonas maltophilia)	795
A0A0M7JN61	Pullulanase secretion envelope pulD	pulD_1 ERS370013_04093	Achromobacter sp.	795
A0A0M7KEM6	Pullulanase secretion envelope pulD	pulD_2 ERS370000_04243	Achromobacter sp.	785
A0A0M7LN65	Pullulanase secretion envelope pulD	pulD_2 ERS370003_03896	Achromobacter sp.	851
A0A0M7LPW1	Pullulanase secretion envelope pulD	pulD_3 ERS369989_05095	Achromobacter sp.	870
A0A0M7MBM2	Pullulanase secretion envelope pulD	pulD_3 ERS369982_05011	Achromobacter sp.	873
A0A0M9GIU5	Type II secretion system protein D	PF66_01622	Pseudomonas fuscovaginae	794
A0A0N0GGN1	General secretion pathway protein D	ABJ99_0811	Pseudomonas syringae pv. cilantro	784
A0A0N1JBQ3	General secretion pathway protein D	AC507_4306	Pseudomonas syringae pv. maculicola	784
A0A0N9WN46	Type II secretion system protein GspD	AO356_24790	Pseudomonas fluorescens	786
A0A0P6S501	General secretion pathway protein GspD	AK821_00955	Pseudomonas sp. RIT-PI-r	789

A0A0P8Z9H9	Type II secretion system protein GspD	APR47_12375 APR48_12675 APR49_03360 APR50_04410 APR51_04535 APR52_11515	Variovorax paradoxus	807
A0A0P9PT68	Proteinral secretion pathway protein D	ALO82_04792	Pseudomonas syringae pv. broussonetiae	841
A0A0P9UCX9	Proteinral secretion pathway protein D	ALO35_05651	Pseudomonas amygdali pv. lachrymans (Pseudomonas syringae pv. lachrymans)	977
A0A0Q0DLS6	Proteinral secretion pathway protein D	ALO56_05058	Pseudomonas viridiflava	850
A0A0Q4NVZ4	Type II secretion system protein GspD	ASF04_16720	Duganella sp. Leaf61	781
A0A0Q5CYY4	Type II secretion system protein GspD	ASF61_13865	Duganella sp. Leaf126	801
A0A0Q6VKG2	Type II secretion system protein GspD	ASD15_20050	Massilia sp. Root351	733
A0A0Q6YHJ1	Type II secretion system protein GspD	ASC85_12180	Pseudomonas sp. Root401	787
A0A0Q8BLB5	Type II secretion system protein GspD	ASD71_22805	Achromobacter sp. Root565	788
A0A0Q8K672	Type II secretion system protein GspD	ASD87_01890	Achromobacter sp. Root170	793
A0A0Q8WQ22	Type II secretion system protein GspD	ASE30_15820	Achromobacter sp. Root83	790
A0A0R0BGK9	Type II secretion system protein GspD	ARC23_07450	Stenotrophomonas maltophilia (Pseudomonas maltophilia) (Xanthomonas maltophilia)	793
A0A0R0BW20	Type II secretion system protein GspD	ARC02_02985	Stenotrophomonas maltophilia (Pseudomonas maltophilia) (Xanthomonas maltophilia)	793
A0A0R0DY42	General secretion pathway protein GspD	ABB31_01270	Stenotrophomonas pavanii	793
A0A0R3B3M6	General secretion pathway protein GspD	TU80_09895	Pseudomonas veronii	767
A0A0S1XVX2	Type II secretion system protein GspD	ASB57_02250	Bordetella sp. N	884
A0A0U1QDT3	General secretion pathway protein GspD	YH64_12160	Achromobacter sp. LC458	804
A0A0U5DE65	General secretion pathway protein D	BN1263230003	Stenotrophomonas maltophilia (Pseudomonas maltophilia) (Xanthomonas maltophilia)	793
A0A0U5HQZ3	General secretion pathway protein D	BN1263210012	Stenotrophomonas maltophilia (Pseudomonas maltophilia) (Xanthomonas maltophilia)	793
A0A0V2NE33	Type II secretion system protein GspD	AO896_15075	Pseudomonas aeruginosa	803
A0A0W0IL03	Type II secretion system protein GspD	AO068_05570	Pseudomonas sp. ICMP 3272	778
A0A0W0LVI2	Type II secretion system protein GspD	AO391_04340	Pseudomonas marginalis ICMP 9505	787
A0A109XVN3	Type II secretion system protein GspD	AL504_06805	Alcaligenes xylosoxydans xylosoxydans (Achromobacter xylosoxidans)	873
A0A126S3D1	General secretion pathway protein D	AWT69_002019	Pseudomonas putida (Arthrobacter siderocapsulatus)	789
A0A143GFQ4	General secretion pathway protein D	AK972_2227	Pseudomonas fluorescens	769
A0A149QGD8	General secretion pathway protein GspD	AB839_11365	Stenotrophomonas sp. DDT-1	793
A0A151QH77	Type II secretion system protein GspD	A0129_06315	Limnobacter sp. CACIAM 66H1	795
A0A157Q1Y8	General secretion pathway protein D	gspD_1 SAMEA1982600_03098	Bordetella ansorpii	879

A0A157QKP7	General secretion pathway protein D	gspD_2 SAMEA1982600_03670	Bordetella ansorpii	968
A0A157QMY7	General secretion pathway protein D	gspD_3 SAMEA1982600_03781	Bordetella ansorpii	959
A0A157SF87	General secretion pathway protein D	gspD_1 SAMEA3906486_02305	Bordetella ansorpii	939
A0A157SLY4	General secretion pathway protein D	gspD_2 SAMEA3906486_03515	Bordetella ansorpii	880
A0A157SS67	General secretion pathway protein D	gspD_3 SAMEA3906486_04391	Bordetella ansorpii	938
A0A159ZS25	Type II secretion system protein GspD	TK06_03225	Pseudomonas fluorescens	787
A0A162ULS7	Type II secretion system protein GspD	A3839_19555	Achromobacter sp. AR476-2	790
A0A171KT29	General secretion pathway protein GspD	AAV32_08345	Kerstersia gyiorum	681
A0A176NI69	Type II secretion system protein GspD	APS14_07145	Pseudomonas thivervalensis	788
A0A176XW46	Type II secretion system protein GspD	A7J67_09675	Alcaligenes xylosoxydans xylosoxydans (Achromobacter xylosoxidans)	793
A0A177XBA5	Type II secretion system protein GspD	AO064_10630	Pseudomonas marginalis	788
A0A193G733	Type II secretion system protein GspD	BAU08_25875	Bordetella bronchialis	845
A0A1A6XYF5	Type II secretion system protein GspD	A9J40_07525	Stenotrophomonas maltophilia (Pseudomonas maltophilia) (Xanthomonas maltophilia)	793
A0A1A9JFM5	Type II secretion system protein GspD	A8L59_14235	Pseudomonas koreensis	792
A0A1B2RAX9	General secretion pathway protein GspD	AKI39_24140	Bordetella sp. H567	836
A0A1B3D7I4	Type II secretion system protein GspD	A7317_10690	Pseudomonas fluorescens	781
A0A1B5ENG7	Pectic enzymes secretion protein OutD	outD_3	Pseudomonas sp. 22 E 5	766
A0A1B5F4C9	General secretion pathway protein D	xcpQ	Pseudomonas sp. 22 E 5	755
A0A1C1W2W0	Type II secretion system protein GspD	BB029_15235	Pseudomonas sp. S3E12	774
A0A1C1W6D3	Type II secretion system protein GspD	BBG20_18990	Pseudomonas sp. S1E40	776
A0A1C3BLZ3	Type II secretion system protein GspD	A7P25_16740	Alcaligenes xylosoxydans xylosoxydans (Achromobacter xylosoxidans)	870
A0A1C3GSR2	General secretion pathway protein D	xcpQ BN1864_LIB5394:03422	Pseudomonas sp. 1 R 17	777
A0A1C3K587	General secretion pathway protein D	ODI_01971	Orrella dioscoreae	920
A0A1C3K7J9	General secretion pathway protein D	ODI_02413	Orrella dioscoreae	958
A0A1D2SK71	Type II secretion system protein GspD	ABS56_03715	Lautropia sp. SCN 69-89	841
A0A1D2TNN0	Type II secretion system protein GspD	ABS55_01245	Lautropia sp. SCN 70-15	825
A0A1E3ZI93	Type II secretion system protein GspD	ABS43_07135	Bordetella sp. SCN 67-23	813
A0A1E4IE78	Type II secretion system protein GspD	ABS91_03755	Thiobacillus sp. SCN 64-35	672
A0A1E4J5K5	Type II secretion system protein GspD	ABS94_07395	Variovorax sp. SCN 67-85	863
A0A1E4W8M1	Type II secretion system protein GspD	A9G05_22375	Pseudomonas sp. ENNP23	778
A0A1F3ZC24	Type II secretion system protein GspD	A2Z64_11360	Betaproteobacteria bacterium RIFCSPLOWO2_02_67_12	693

A0A1H1Q8K2	General secretion pathway protein D	SAMN05216198_1412	Pseudomonas litoralis	883
A0A1H1RY05	General secretion pathway protein D	SAMN04490182_1465	Pseudomonas cedrina	774
A0A1H1W5M4	General secretion pathway protein D	SAMN05216579_2000	Pseudomonas granadensis	795
A0A1H1XF97	General secretion pathway protein D	SAMN05216585_4503	Pseudomonas chlororaphis	805
A0A1H1Z7Z0	General secretion pathway protein D	SAMN05216598_4892	Pseudomonas asplenii	797
A0A1H2M925	General secretion pathway protein D	SAMN04490181_1161	Pseudomonas brenneri	766
A0A1H2MHL8	General secretion pathway protein D	SAMN05216202_1625	Pseudomonas mucidolens	775
A0A1H2NPF6	General secretion pathway protein D	SAMN04490209_2712	Pseudomonas rhodesiae	772
A0A1H3FSP3	General secretion pathway protein D	SAMN05216247_102245	Pseudomonas salomonii	782
A0A1I1A800	General secretion pathway protein D	SAMN05216248_104411	Pseudomonas simiae	764
A0A1I4SQT7	General secretion pathway protein D	SAMN05444064_12524	Pseudomonas syringae	781
A0A1I6JFZ8	Type II secretion system protein D (GspD)	SAMN04487782_0279	Stenotrophomonas maltophilia (Pseudomonas maltophilia) (Xanthomonas maltophilia)	791
A0A1K1LTE7	General secretion pathway protein D	SAMN03159439_00179	Pseudomonas sp. NFACC04-2	787
A0A1K1STL7	General secretion pathway protein D	SAMN05660640_04693	Pseudomonas sp. NFACC16-2	786
A0A1K1UMP6	General secretion pathway protein D	SAMN03159479_01015	Pseudomonas sp. NFPP09	794
A0A1K1Y0U0	General secretion pathway protein D	SAMN03159442_02055	Pseudomonas sp. NFACC47-1	786
A0A1M5NUK2	Type II secretion system protein D (GspD)	SAMN04488135_101622	Candidimonas bauzanensis	822
A0A1M6BEM3	General secretion pathway protein D	SAMN04488135_12512	Candidimonas bauzanensis	915
A0A1N7G1L0	General secretion pathway protein D	SAMN05216504_4016	Pseudomonas sp. A214	784
A0A1N7RCB4	Type II secretion system protein D (GspD)	SAMN05428937_5431	Achromobacter sp. MFA1 R4	817
A0A1Q7IQ90	Type II secretion system protein GspD	AUH79_06990	Betaproteobacteria bacterium 13_1_40CM_4_64_4	695
A0A1Q7VHG1	Type II secretion system protein GspD	AUG50_07380	Betaproteobacteria bacterium 13_1_20CM_3_63_8	700
A0A1Q8EQJ7	Type II secretion system protein GspD	BTN82_13355	Pseudomonas chlororaphis	804
A0A1Q9WSE8	Type II secretion system protein GspD	BVK87_27570	Achromobacter denitrificans (Alcaligenes denitrificans)	870
A0A1R0G9T9	Type II secretion system protein GspD	AU074_07535	Pseudomonas sp. ATCC PTA-122608	768
A0A1S2BR35	Type II secretion system protein GspD	BB780_09115	Stenotrophomonas maltophilia (Pseudomonas maltophilia) (Xanthomonas maltophilia)	793
A0A1S2V223	Type II secretion system protein GspD	BFL40_14315	Pseudomonas costantinii	773
A0A1T1HJC2	Type II secretion system protein GspD	MF4836_32330	Pseudomonas sp. MF4836	788
A6GLP8	Type II and III secretion system protein	LMED105_05252	Limnobacter sp. MED105	807
A9IRR4	General secretion pathway protein D	gspD Bpet2865	Bordetella petrii (strain ATCC BAA-461 / DSM 12804 / CCUG 43448)	862

B4SL34	General secretion pathway protein D	Smal_2211	Stenotrophomonas maltophilia (strain R551-3)	793
C3K8J8	Putative type II secretion pathway protein D	PFLU_2423	Pseudomonas fluorescens (strain SBW25)	770
E3HR19	General secretion pathway protein D	gspD AXYL_02659	Achromobacter xylosoxidans (strain A8)	790
F4GSU2	General secretion pathway protein D	PT7_1144	Pusillimonas sp. (strain T7-7)	792
G0K2F2	General secretion pathway protein D	BurJV3_2260	Stenotrophomonas maltophilia JV3	793
H0F1U5	General secretion pathway protein D	KYC_03657	Achromobacter arsenitoxydans SY8	794
J0UNT9	General secretion pathway protein D	QWA_10806	Alcaligenes faecalis subsp. faecalis NCIB 8687	770
J1IQM3	General secretion pathway protein D	A462_03524	Pseudomonas sp. Ag1	769
J2MR24	General secretion pathway protein D	gspD PflQ2_3149	Pseudomonas fluorescens Q2-87	788
J2R4M0	General secretion pathway protein D	PMI23_01086	Pseudomonas sp. GM24	788
J2STL5	General secretion pathway protein D	PMI30_03322	Pseudomonas sp. GM50	791
J2YH54	Type II secretion protein, secretin HxcQ	hxcQ Pchl3084_2689	Pseudomonas chlororaphis subsp. aureofaciens 30-84	803
J4QKI9	General secretion pathway protein D	QWC_27491	Achromobacter piechaudii HLE	792
K0WKN0	General secretion pathway protein D	I1A_003007	Pseudomonas fluorescens R124	792
L9PM96	General secretion pathway protein D	gspD Jab_1c05580	Janthinobacterium sp. HH01	750
Q1IB24	Putative type II secretion system protein XcpQ-2 putative signal peptide	PSEEN2332	Pseudomonas entomophila (strain L48)	787
Q2KZP0	General secretion pathway protein D	gspD BAV0333	Bordetella avium (strain 197N)	860
U1ZMJ4	General secretion pathway protein D	N879_08325	Alcaligenes sp. EGD-AK7	770
U3HJZ0	Uncharacterized protein	L682_31615	Pseudomonas alcaligenes OT 69	793
U6ZLY6	General secretion pathway protein D	P308_14265	Pseudomonas sp. CMAA1215	797
V8R6M2	General secretion pathway protein D	PMO01_14565	Pseudomonas moraviensis R28-S	795
W2DHG2	General secretion pathway protein D	H096_22773	Pseudomonas sp. FH1	767
W8X971	General secretion pathway protein D	BN940_09271	Castellaniella defragrans 65Phen	780
A0A063BJ98	General secretion pathway protein D	LIG30_1917	Burkholderia sp. lig30	752
A0A063BN64	General secretion pathway protein D	LIG30_0843	Burkholderia sp. lig30	774
A0A069IF75	General secretion pathway protein GspD	CF70_032030	Cupriavidus sp. SK-3	810
A0A069P5E9	General secretion pathway protein GspD	BG57_24715	Caballeronia grimmiae	814
A0A069PIT2	General secretion pathway protein D (General secretion pathway protein GspD)	BG61_30030 BGLT_05776	Caballeronia glathei	789
A0A071MDU6	General secretion pathway protein GspD	DT99_14655	Burkholderia cenocepacia	779
A0A081V8L2	General secretion pathway protein GspD	HR51_24110	Burkholderia cepacia (Pseudomonas cepacia)	775
A0A084DNI1	General secretion pathway protein GspD	GQ57_03620	Burkholderia sp. MSh2	772
A0A086W5K9	General secretion pathway protein GspD	JN27_21525	Massilia sp. BSC265	761

A0A087NMV8	General secretion pathway protein GspD	JM78_28255	Burkholderia pyrrocinia (Pseudomonas pyrrocinia)	774
A0A088U867	Type II secretion system protein D	gspD DM39_3336	Burkholderia cenocepacia	770
A0A088XJR2	Type II secretion system protein D	gspD DM82_3164	Burkholderia oklahomensis	750
A0A095GFW6	General secretion pathway protein GspD (Type II secretion system protein D)	gspD DM48_4199 XF14_21725	Burkholderia gladioli (Pseudomonas marginata) (Phytomonas marginata)	787
A0A096YK72	Type II secretion system protein D	gspD DR63_1786	Burkholderia thailandensis (strain ATCC 700388 / DSM 13276 / CIP 106301 / E264)	751
A0A0A1FCS5	General secretion pathway protein D	LT85_3113	Collimonas arenae	740
A0A0A1H8R9	General secretory pathway component, cryptic	E1O_25480	Burkholderiales bacterium GJ-E10	751
A0A0A6QG77	General secretion pathway protein GspD	NH14_04980	Paraburkholderia sacchari	789
A0A0B1Y375	General secretion pathway protein GspD	PI87_20240	Ralstonia sp. A12	802
A0A0B1Y5A4	General secretion pathway protein GspD	PI86_15020	Burkholderia sp. A9	770
A0A0B6RQW4	General secretion pathway protein D	gspD BGL_1c00350	Burkholderia glumae PG1	782
A0A0C1YAC8	General secretion pathway protein GspD	TSA66_22485	Herbaspirillum sp. TSA66	731
A0A0C1Z0Y4	General secretion pathway protein D	BurMR1_1005	Burkholderia sp. MR1	821
A0A0C4YKZ2	General secretion pathway protein D	RR42_m3966	Cupriavidus basilensis	809
A0A0D0HCB8	GspD protein	gspD KY49_5459	Burkholderia sp. MSHR3999	767
A0A0D5AKN2	General secretion pathway protein GspD	TK49_01520	Ralstonia mannitolilytica	805
A0A0D5DW12	General secretion pathway protein GspD	DM57_1547	Burkholderia mallei (Pseudomonas mallei)	757
A0A0D5LEX6	Type II secretion system protein D	gspD BW21_192	Burkholderia sp. 2002721687	756
A0A0D5VBQ1	Type II secretion system protein D	gspD OI25_1505	Paraburkholderia fungorum	790
A0A0D8E0F8	General secretion pathway protein GspD	PL79_12370	Burkholderia sp. USM B20	780
A0A0E4AHZ0	General secretion pathway protein GspD	XM57_04550	Burkholderia cepacia (Pseudomonas cepacia)	780
A0A0E9MAZ7	Type II secretion system protein D	xcpQ OYT1_02653	Ferriphaselus amnicola	704
A0A0F0D9T0	General secretion pathway protein GspD	UB44_25645	Burkholderiaceae bacterium 26	806
A0A0F0FPS0	General secretion pathway protein GspD	UB46_21665	Burkholderiaceae bacterium 16	804
A0A0F5K3C7	Uncharacterized protein	WM40_06110	Paraburkholderia andropogonis	847
A0A0G3WNG5	General secretion pathway protein GspD	ABD05_06350	Burkholderia pyrrocinia (Pseudomonas pyrrocinia)	769
A0A0G3YX92	General secretion pathway protein GspD	NL30_19190	Burkholderia contaminans	780
A0A0H2XM31	Type II secretion system gspD	Bcen_0008	Burkholderia cenocepacia (strain AU 1054)	774
A0A0J1CJ21	General secretion pathway protein GspD	EOS_40010	Caballeronia glathei PML1(12)	696
A0A0J1CRE9	General secretion pathway protein GspD	EOS_26885	Caballeronia glathei PML1(12)	772
A0A0J1CWB5	General secretion pathway protein GspD	EOS_17900	Caballeronia glathei PML1(12)	800

A0A0J5WTB1	General secretion pathway protein GspD	VL15_19650	Burkholderia cepacia (Pseudomonas cepacia)	772
A0A0J5YRJ7	General secretion pathway protein GspD	VL00_17175	Burkholderia lata (strain ATCC 17760 / LMG 22485 / NCIMB 9086 / R18194 / 383)	781
A0A0J7JRJ5	General secretion pathway protein D	BPMI_04050c	Candidatus Burkholderia pumila	799
A0A0J9DT65	General secretion pathway protein GspD	AC240_11490	Ralstonia sp. MD27	804
A0A0K1K0W9	General secretion pathway protein GspD	ACZ75_17505	Massilia sp. NR 4-1	747
A0A0K9JX73	General secretion pathway protein D	BHUM_05170c	Candidatus Burkholderia humilis	631
A0A0L0MFJ4	General secretion pathway protein D	BVER_04404c	Candidatus Burkholderia verschuerenii	634
A0A0L1L1D3	General secretion pathway protein D	BRCH_00612c	Candidatus Burkholderia brachyanthoides	798
A0A0M3TW93	General secretion pathway protein GspD	AC233_17835	Burkholderia sp. HB1	781
A0A0N0ICS6	General secretion pathway protein GspD	ADM96_36025	Burkholderia sp. ST111	776
A0A0N0XI54	Type II secretion system protein D	xcpQ WG78_18075	Amantichitinum ursilacus	759
A0A0P0R6A3	General secretion pathway protein D	K788_0003580	Paraburkholderia caribensis MBA4	787
A0A0Q9P8R9	Type II secretion system protein GspD	ASG75_01885	Rhodanobacter sp. Soil772	696
A0A0R0LGW8	Putative type II secretion system protein D	gspD FERRO_12660	Ferrovum sp. JA12	762
A0A0S4TXW8	General secretion pathway protein D (General secretion pathway protein GspD)	RSP799_01125 RUN39_v1_970014	Ralstonia solanacearum (Pseudomonas solanacearum)	806
A0A0S4VNS7	General secretion pathway protein D (Type II secretion system protein GspD)	AC251_01655 RD1301_v1_1220004 RUN1744_v1_40027 RUN1985_v1_190047 TD1301_v1_1960004 TF3108_v1_50040	Ralstonia solanacearum (Pseudomonas solanacearum)	806
A0A0U4DQR1	Type II secretion system protein GspD	P350_01830	Burkholderia cepacia JBK9	780
A0A102ESW1	Type II secretion system protein GspD	WS45_12450	Burkholderia sp. RF2-non_BP3	775
A0A102K0H7	Type II secretion system protein GspD	WI38_07810	Burkholderia ubonensis	774
A0A103E227	Type II secretion system protein GspD	WS67_13390	Burkholderia sp. TSV85	773
A0A103FK05	Type II secretion system protein GspD	WS71_04580	Burkholderia sp. BDU8	756
A0A103GHS0	Type II secretion system protein GspD	WJ03_15765	Burkholderia vietnamiensis	775
A0A103QW14	Type II secretion system protein GspD	WJ33_36065	Burkholderia ubonensis	774
A0A104GV72	Type II secretion system protein GspD	WL80_31175	Burkholderia ubonensis	770
A0A104MR15	Type II secretion system protein GspD	WJ63_19920	Burkholderia pyrrocinia (Pseudomonas pyrrocinia)	772
A0A105ZFC8	Type II secretion system protein GspD	WK95_00845	Burkholderia ubonensis	778
A0A108D5P9	Type II secretion system protein GspD	WM16_28050	Burkholderia ubonensis	783
A0A109E7G0	Type II secretion system protein GspD	AS149_11280	Burkholderia cenocepacia	781
A0A118CPQ7	Type II secretion system protein GspD	WS61_19300	Burkholderia sp. ABCPW 11	780
A0A118DU30	Type II secretion system protein GspD	WS68_07880	Burkholderia sp. TSV86	766

A0A118G1A7	Type II secretion system protein GspD	WS75_27175	Burkholderia sp. FL-7-2-10-S1-D7	774
A0A119AM87	Type II secretion system protein GspD	WT27_17140	Burkholderia territorii	775
A0A119DI96	Type II secretion system protein GspD	WT32_26090	Burkholderia anthina	780
A0A124PE77	Type II secretion system protein GspD	WS70_09535	Burkholderia sp. BDU6	758
A0A124SQE9	Type II secretion system protein GspD	WS90_04770	Burkholderia cepacia (Pseudomonas cepacia)	776
A0A127QPV0	Type II secretion system protein D	gspD CAter282_4155	Collimonas arenae	764
A0A132CUV2	Type II secretion system protein GspD	WS66_09080	Burkholderia sp. LA-2-3-30-S1-D2	774
A0A132HA60	Type II secretion system protein D	xcpQ AU374_04685	Cupriavidus metallidurans	783
A0A142JDW9	Type II secretion system protein GspD	A2G96_00140	Cupriavidus nantongensis	832
A0A149PXE0	Type II secretion system protein GspD	CI15_05990	Paraburkholderia monticola	794
A0A149QEE2	Type II secretion system protein GspD	CR51_24505	Caballeronia megalochromosomata	808
A0A149VTF1	Type II secretion system protein D	xcpQ FV185_04500	Ferrovum sp. PN-J185	770
A0A157Z2E6	General secretory pathway protein D	AWB75_00085	Burkholderia catudaia	795
A0A157Z3F5	Type II and III secretion system protein	AWB77_00173	Burkholderia fortuita	796
A0A157Z9T6	General secretory pathway protein D	AWB78_00299	Burkholderia calidae	791
A0A157Z9Z9	Type II and III secretion system protein	AWB79_00569	Burkholderia hypogeia	805
A0A157ZAM6	Type II and III secretion system protein	AWB81_00126	Burkholderia arationis	797
A0A157ZFH6	Type II and III secretion system protein	AWB76_00596	Burkholderia temeraria	796
A0A158A042	General secretion pathway protein D	AWB80_01570	Burkholderia pedi	789
A0A158A1I2	General secretion pathway protein D	AWB82_01559	Burkholderia glebae	796
A0A158DCA2	Type II and III secretion system protein	AWB83_05313	Burkholderia ptereochthonis	793
A0A158FDR4	General secretion pathway protein D	AWB64_01119	Caballeronia sordidicola	817
A0A158FFP5	Type II and III secretion system protein	AWB73_01048	Burkholderia turbans	801
A0A158H7Q6	General secretion pathway protein D	AWB69_04029	Caballeronia udeis	807
A0A158HB52	General secretory pathway protein D	AWB74_01639	Burkholderia arvi	794
A0A158HWP2	General secretion pathway protein D	AWB71_02685	Burkholderia peredens	789
A0A161HPD4	Type II secretion system protein GspD	AYM40_20250	Burkholderia sp. OLGA172	781
A0A192A0V5	Type II secretion system protein GspD	A9Y76_16580	Ralstonia insidiosa	804
A0A1A5X6X5	Type II secretion system protein GspD	A6456_00365	Paraburkholderia tropica	778
A0A1A9NDG3	Type II secretion system protein GspD	A6V37_01330	Paraburkholderia ginsengiterrae	794
A0A1B4HPG3	Type II secretion system protein GspD	WJ16_00320	Burkholderia metallica	781
A0A1B4I7S8	Type II secretion system protein GspD	WJ23_00445	Burkholderia lata (strain ATCC 17760 / LMG 22485 / NCIMB 9086 / R18194 / 383)	776
A0A1B4LEH2	Type II secretion system protein GspD	WJ35_11045	Burkholderia ubonensis	777

A0A1B4MPX7	Type II secretion system protein GspD	WK22_00280	Burkholderia multivorans	769
A0A1B4NP57	Type II secretion system protein GspD	WK25_02370	Burkholderia latens	773
A0A1B4PMP4	Type II secretion system protein GspD	WT26_03675	Burkholderia cepacia (Pseudomonas cepacia)	777
A0A1C0XHF7	Type II secretion system protein GspD	BEK67_16125	Ralstonia pickettii (Burkholderia pickettii)	805
A0A1C3VZJ8	Type II secretion system protein D (GspD)	GA0116996_11428	Cupriavidus alkaliphilus	829
A0A1D2SAK9	Type II secretion system protein GspD	ABS45_03620	Comamonas sp. SCN 65-56	773
A0A1D7ZDE3	Type II secretion system protein GspD	BBJ41_09900	Burkholderia stabilis	776
A0A1D9H300	Type II secretion system protein GspD	BKK79_12070	Cupriavidus sp. USMAA2-4	790
A0A1E4G0H5	Type II secretion system protein GspD	ABS69_08980	Nitrosomonadales bacterium SCN 54-20	789
A0A1E4G548	Type II secretion system protein GspD	ABS69_07855	Nitrosomonadales bacterium SCN 54-20	784
A0A1E4NAZ5	Type II secretion system protein GspD	ABT21_11680	Thiobacillus sp. SCN 65-179	671
A0A1E7UPM2	Putative type II secretion system protein D	gspD JAB1_23610	Janthinobacterium sp. MP5059B	749
A0A1E7W3V9	Putative type II secretion system protein D	gspD JAB8_21620	Janthinobacterium sp. HH106	743
A0A1E7X632	Putative type II secretion system protein D	gspD DUPY_08400	Duganella phyllosphaerae	783
A0A1E8PRU2	Type II secretion system protein GspD	BA896_007280	Janthinobacterium lividum	750
A0A1F4B2T0	Type II secretion system protein GspD	A3I63_10795	Betaproteobacteria bacterium RIFCSPLOWO2_02_FULL_66_14	652
A0A1H0YRT4	General secretion pathway protein D	SAMN05443245_0244	Paraburkholderia fungorum	784
A0A1H1EGQ8	Type II secretion system protein D (GspD)	SAMN05216402_2660	Nitrosospira multiformis	793
A0A1H1JHU5	General secretion pathway protein D	SAMN05443245_6444	Paraburkholderia fungorum	700
A0A1H3XB62	Type II secretion system protein D (GspD)	SAMN05216411_103131	Nitrosospira multiformis	789
A0A1H7EFY3	General secretion pathway protein D	SAMN05216550_12252	Paraburkholderia tropica	705
A0A1H8HGX1	Type II secretion system protein D (GspD)	SAMN05216404_105134	Nitrosospira multiformis	789
A0A1H9Z4E9	Type II secretion system protein D (GspD)	SAMN05216412_101495	Nitrosospira multiformis	789
A0A1I7FRR8	Type II secretion system protein D (GspD)	SAMN05216417_102135	Nitrosospira multiformis	783
A0A1I9YIP7	Type II secretion system protein GspD	BJG93_12800	Paraburkholderia sprentiae WSM5005	798
A0A1J9NJQ0	Type II secretion system protein GspD	BFF94_15655	Burkholderia sp. DNA89	767
A0A1K1S5R5	Type II secretion system protein D (GspD)	SAMN03159384_05144	Burkholderia sp. NFACC33-1	771
A0A1L6HS93	Type II secretion system protein GspD	BTO02_00690	Paraburkholderia sp. SOS3	815
A0A1L9GMW0	Type II secretion system protein GspD	AOC28_06765	Polynucleobacter sp. MWH-Adler-W8	756
A0A1M6LLH1	General secretion pathway protein D	SAMN05192548_1005194	Paraburkholderia terricola	788
A0A1N6EHW9	General secretion pathway protein D	SAMN05444168_0662	Paraburkholderia phenazinium	795
A0A1N6GLF9	General secretion pathway protein D	SAMN05444172_0078	Burkholderia sp. GAS332	797
A0A1N6GQD3	Type II secretion system protein D (GspD)	SAMN05444165_0903	Paraburkholderia phenazinium	791

A0A1N6L735	General secretion pathway protein D	SAMN05444165_6366	Paraburkholderia phenazinium	784
A0A1N7RSR5	General secretion pathway protein D	BN2475_140037	Paraburkholderia ribeironis	790
A0A1N7S437	General secretion pathway protein D	BN2475_340092	Paraburkholderia ribeironis	782
A0A1N7SQW3	General secretion pathway protein D	BN2476_700085	Paraburkholderia piptadeniae	812
A0A1P8FK66	Type II secretion system protein GspD	BWI17_12455	Betaproteobacteria bacterium GR16-43	698
A0A1Q3VJX3	Type II secretion system protein GspD	BGO50_07150	Rhodanobacter sp. 67-28	699
A0A1Q3YKD1	Type II secretion system protein GspD	BGO72_21110	Burkholderiales bacterium 70-64	781
A0A1Q4LKZ6	Type II secretion system protein GspD	BH759_18915	Ralstonia solanacearum (Pseudomonas solanacearum)	810
A0A1Q8IRV7	Type II secretion system protein GspD	BTH42_23220	Burkholderia sp. SRS-W-2-2016	768
A0A1R1J469	Type II secretion system protein GspD	BW685_30385	Burkholderia ubonensis	774
A0A1R3W0C4	General secretion pathway protein D	SAMN04487768_2127	Burkholderia sp. b13	803
B1T6A2	General secretion pathway protein D	BamMEX5DRAFT_3318	Burkholderia ambifaria MEX-5	775
B2JJP0	General secretion pathway protein D	Bphy_3070	Paraburkholderia phymatum (strain DSM 17167 / CIP 108236 / LMG 21445 / STM815) (Burkholderia phymatum)	787
B2JXL4	General secretion pathway protein D	Bphy_7395	Paraburkholderia phymatum (strain DSM 17167 / CIP 108236 / LMG 21445 / STM815) (Burkholderia phymatum)	717
B2T7P2	General secretion pathway protein D	Bphyt_3936	Paraburkholderia phytofirmans (strain DSM 17436 / LMG 22146 / PsJN) (Burkholderia phytofirmans)	791
B2UFF1	General secretion pathway protein D	Rpic_3399	Ralstonia pickettii (strain 12J)	807
B3R7X2	General Secretory Pathway protein D, type II secretion system	gspD RALTA_A2996	Cupriavidus taiwanensis (strain DSM 17343 / BCRC 17206 / CIP 107171 / LMG 19424 / R1) (Ralstonia taiwanensis (strain LMG 19424))	831
B5WD07	General secretion pathway protein D	BH160DRAFT_0957	Burkholderia sp. H160	792
C5A834	General secretory pathway protein D	bglu_1g00380	Burkholderia glumae (strain BGR1)	765
D5CMQ3	General secretion pathway protein D	Slit_0499	Sideroxydans lithotrophicus (strain ES-1)	690
D5W742	General secretion pathway protein D	BC1002_3143	Burkholderia sp. (strain CCGE1002)	811
E1T3W0	General secretion pathway protein D	BC1003_3488	Burkholderia sp. (strain CCGE1003)	782
E5AKF9	General secretion pathway protein D	RBRH_00782	Paraburkholderia rhizoxinica (strain DSM 19002 / CIP 109453 / HKI 454) (Burkholderia rhizoxinica)	847
E7RWU0	General secretion pathway protein D	gspD HMPREF0551_1152	Lautropia mirabilis ATCC 51599	837
G0EZE4	General secretion pathway protein D	gspD CNE_1c34900	Cupriavidus necator (strain ATCC 43291 / DSM 13513 / N-1) (Ralstonia eutropha)	812
G2JAB6	General secretion pathway protein D	gspD CAGGBEG34_290009	Candidatus Glomeribacter gigasporarum BEG34	750

G3A240	General Secretory Pathway protein D, type II secretion system	gspD RALSY_20068	Ralstonia syzygii R24	807
G4MJT4	General secretion pathway protein D	BKIR_c95_1647	Candidatus Paraburkholderia kirkii UZHbot1	798
G8MB45	Type II and III secretion system protein:NolW-like protein	nolW BYI23_A026910	Burkholderia sp. YI23	803
I4VMQ3	Type II and III secretion system protein	UU9_12123	Rhodanobacter fulvus Jip2	704
I9W516	General secretory pathway component, cryptic	MW7_2219	Ralstonia sp. PBA	772
K0DUB3	General secretion pathway protein D	BUPH_03878	Paraburkholderia phenoliruptrix BR3459a	789
K8R523	General secretion pathway protein D	BURK_029230	Burkholderia sp. SJ98	795
K8RK33	General secretion pathway protein D	BURK_011241	Burkholderia sp. SJ98	685
Q0BJR0	General secretion pathway protein D	Bamb_0052	Burkholderia ambifaria (strain ATCC BAA-244 / AMMD) (Burkholderia cepacia (strain AMMD))	777
Q0K5W9	General secretion pathway protein D	gspD H16_A3543	Cupriavidus necator (strain ATCC 17699 / H16 / DSM 428 / Stanier 337) (Ralstonia eutropha)	812
Q13SK8	Putative general secretory pathway protein D	Bxe_A4501	Paraburkholderia xenovorans (strain LB400)	814
Q39L20	Type II and III secretion system protein	Bcep18194_A3244	Burkholderia lata (strain ATCC 17760 / LMG 22485 / NCIMB 9086 / R18194 / 383)	781
Q46W94	Type II and III secretion system protein:NolW-like protein	Reut_A3229	Cupriavidus necator (strain JMP 134 / LMG 1197) (Ralstonia eutropha (strain JMP 134))	804
Q5P193	General secretory pathway protein D	gspD2 ebA4915	Aromatoleum aromaticum (strain EbN1) (Azoarcus sp. (strain EbN1))	719
Q9F1Q1	GspD	gspD	Burkholderia cepacia (Pseudomonas cepacia)	783
R4WYW7	General secretion pathway protein D	gspD BRPE64_ACDS28070	Burkholderia sp. RPE64	786
U3QW29	General secretion pathway protein D	N234_19810	Ralstonia pickettii DTP0602	816

Other Gammaproteobacteria

Entry	Protein names	Gene names	Organism	Length
A0A0Q4T9N5	Uncharacterized protein	ASF15_05435	Pseudomonas sp. Leaf83	639
A0A191ZHS1	Type II secretion system protein GspD	A9404_08570	Halothiobacillus sp. LS2	730
A0A084IQP0	General secretion pathway protein D	C41B8_02802	Salinisphaera hydrothermalis C41B8	716
F7Q2Y0	Type II secretion system protein D	xcpQ SSPSH_003278	Salinisphaera shabanensis E1L3A	694
A0A0X3TFJ5	Type II secretion system protein GspD	AVO42_03890	Thiomicrospira sp. XS5	710
F6DD37	General secretion pathway protein D	Thicy_1006	Thioalkalimicrobium cyclicum (strain DSM 14477 / JCM 11371 / ALM1)	694
Q31J27	Type II secretion pathway protein D	Tcr_0250	Thiomicrospira crunogena (strain XCL-2)	703
A0A1C9W9Q5	Type II secretion system protein D	pulD AUP74_02483	Microbulbifer sp. CCB-MM1	656
A0A1I1EJQ3	General secretion pathway protein D	SAMN05660479_00686	Microbulbifer thermotolerans	659
A0A1M4UF42	General secretion pathway protein D	SAMN04487965_0138	Microbulbifer donghaiensis	659
A0A1Q2M9Q1	Type II secretion system protein GspD	Mag101_08220	Microbulbifer agarilyticus	626
A0YBE1	Type II and III secretion system protein	GP2143_05455	marine gamma proteobacterium HTCC2143	630
A0A0X3UAB3	Type II secretion system protein GspD	AVO43_04015	Microbulbifer sp. ZGT114	633
A0A101LL87	Uncharacterized protein	AR540_07205	Pseudomonas sp. EpS/L25	589
A0A1A9K5R6	Type II secretion system protein GspD	A9C11_01655	Pseudomonas citronellolis	574
A0A1I0UTP9	General secretion pathway protein D	SAMN05216263_1261	Pseudomonas otitidis	644
A0A1N6N4K2	Type II secretion system protein D (GspD)	SAMN05878282_10110	Pseudomonas alcaligenes	616
U3HHE1	Uncharacterized protein	L682_06885	Pseudomonas alcaligenes OT 69	636
A0A0B0H680	Type II protein secretion system GspDSCFGHIJKLMEO, subunit D	gspD JV46_18860	Solemya velum gill symbiont	650
A0A095X3A9	General secretion pathway protein D / Type II secretion outermembrane pore forming protein (PulD)	HRUBRA_00059	Pseudohaliea rubra DSM 19751	674
A0A0K0XXC2	General secretion pathway protein GspD	WM2015_1895	Wenzhouxiangella marina	638
A0A127F9X3	Uncharacterized protein	ACG33_08940	Steroidobacter denitrificans	700
A0A165UCP7	Type II secretion system protein GspD	A3709_14325	Halioglobus sp. HI00S01	671
A0A193LFF4	Type II secretion system protein GspD	BA177_08185	Woeseia oceani	653
A0A1B1YRZ8	Uncharacterized protein	PG2T_04540	Immundisolibacter cernigliae	644
A0A1E2UVV6	Type II secretion system protein GspD	A3196_16225	Candidatus Thiodiazotropha endoloripes	528
A0A1E2UXH9	Type II secretion system protein GspD	A3197_14590	Candidatus Thiodiazotropha endoloripes	528
A0A1E4KF15	Type II secretion system protein GspD	ABS96_33695	Xanthomonadaceae bacterium SCN 69-123	697

A0A1M5KEQ8	General secretion pathway protein D	SAMN04488068_0529	Hydrocarboniphaga daqingensis	683
A0A1Q6WCM3	Type II secretion system protein GspD	AUH10_11265	Gammaproteobacteria bacterium 13_2_20CM_66_19	688
D6Z6F6	General secretion pathway protein D	DaAHT2_2256	Desulfurivibrio alkaliphilus (strain DSM 19089 / UNIQEM U267 / AHT2)	690
I8I1J9	Uncharacterized protein	WQQ_31680	Hydrocarboniphaga effusa AP103	707
A0A0S2KFE7	General secretion pathway protein GspD	PS2015_2398	Pseudohongiella spirulinae	652
A0A1D2QSK8	Type II secretion system protein GspD	AB835_03055	Candidatus Endobugula sertula (Bugula neritina bacterial symbiont)	652
A0A1E8CG37	Type II secretion system protein GspD	PHACT_15425	Pseudohongiella acticola	668
A0Z790	Helix-turn-helix, AraC type	MGP2080_15389	marine gamma proteobacterium HTCC2080	696
A4A5J7	General secretion pathway protein D	KT71_10582	Congregibacter litoralis KT71	696
B3PF18	General secretion pathway protein D	gspD CJA_3332	Cellvibrio japonicus (strain Ueda107) (Pseudomonas fluorescens subsp. cellulosa)	681
B8KIZ6	General secretion pathway protein D	gspD_1 NOR53_1392	gamma proteobacterium NOR5-3	682
B8KQM8	General secretion pathway protein D	gspD_1 NOR51B_2071	Luminiphilus syltensis NOR5-1B	682
C5BLN9	General secretion pathway protein D	gspD TERTU_0265	Teredinibacter turnerae (strain ATCC 39867 / T7901)	661
E0XQJ4	Type II secretory pathway, component puld		uncultured gamma proteobacterium HF0010_05D02	666
F3L1F7	General secretion pathway protein D	IMCC3088_1297	gamma proteobacterium IMCC3088	672
F3LEQ6	Helix-turn-helix, AraC type	IMCC1989_2557	gamma proteobacterium IMCC1989	659
H3NY31	General secretion pathway protein D	OMB55_00004720	gamma proteobacterium HIMB55	667
I3IFG7	General secretion pathway protein D	gspD O59_000823	Cellvibrio sp. BR	685
Q21EP4	Type II and III secretion system protein	Sde_3580	Saccharophagus degradans (strain 2-40 / ATCC 43961 / DSM 17024)	666
R9S595	General secretion pathway protein D	M5M_12147	Simiduia agarivorans (strain DSM 21679 / JCM 13881 / BCRC 17597 / SA1)	667
A0A078M9X3	General secretion pathway protein D	xcpQ BN1049_00666	Pseudomonas saudimassiliensis	684
A0A090BUC9	General secretion pathway protein D	THII_0589	Thioploca ingrica	635
A0A0G3G3V5	General secretion pathway protein GspD	TVD_01695	Thioalkalivibrio versutus	639
A0A0N7FNQ3	General secretion pathway protein GspD	AL038_10365	Beggiatoa leptomitiformis	690
A0A0S8DUU2	Type II secretion system protein GspD	AMJ69_04370	Gammaproteobacteria bacterium SG8_47	652
A0A0T5YZ53	Type II secretion system protein D	Ga0074115_12422	endosymbiont of Ridgeia piscesae	659
A0A0X8X6M5	General secretion pathway protein D	HH1059_269	Halorhodospira halochloris (Ectothiorhodospira halochloris)	618
A0A110B1Z4	General secretion pathway protein D	HH1059_1615	Halorhodospira halochloris (Ectothiorhodospira halochloris)	670
A0A126T8G0	Type II secretion system protein GspD	JT25_017850	Methylomonas denitrificans	635

A0A167A8X7	General secretion pathway protein GspD	ECTOBSL9_0810	Ectothiorhodospira sp. BSL-9	657
A0A167GJA5	General secretion pathway protein GspD	I596_586	Dokdonella koreensis DS-123	685
A0A177M6Y8	Type II secretion system protein GspD	A1332_03610	Methylomonas methanica	635
A0A1E2ZHX7	Type II secretion system protein D	xcpQ CODIS_09750	Candidatus Thiodiazotropha endolucinida	668
A0A1E4K9H3	Type II secretion system protein GspD	ABS97_03415	Xanthomonadaceae bacterium SCN 69-320	691
A0A1E4ZX66	Type II secretion system protein GspD	AB833_15375	Chromatiales bacterium (ex Bugula neritina AB1)	980
A0A1F9M555	Type II secretion system protein GspD	A2505_00330	Deltaproteobacteria bacterium RIFOXYD12_FULL_55_16	671
A0A1F9MC20	Type II secretion system protein GspD	A2511_12640	Deltaproteobacteria bacterium RIFOXYD12_FULL_50_9	658
A0A1H1MKV7	General secretion pathway protein D	SAMN05216198_0651	Pseudomonas litoralis	683
A0A1H1UC70	General secretion pathway protein D	SAMN05216421_2017	Pseudomonas xinjiangensis	692
A0A1H1XP65	General secretion pathway protein D	SAMN05216271_3610	Pseudomonas sabulinigri	679
A0A1H2GBD0	General secretion pathway protein D	SAMN05216210_2188	Pseudomonas salegens	679
A0A1H6BGC6	General secretion pathway protein D	SAMN05216586_11120	Pseudomonas aestusnigri	681
A0A1H9Q0G9	General secretion pathway protein D (Type II secretion system protein D (GspD))	SAMN04487855_0771 SAMN05216589_0854	Pseudomonas bauzanensis	682
A0A1Q4FA19	Type II secretion system protein GspD	BGP25_04365	Xanthomonadales bacterium 63-13	685
A0A1S8DL22	Type II secretion system protein GspD	BXT89_03475	Pseudomonas pachastrellae	682
A0A1T2L909	Type II secretion system protein GspD	BOW53_03500	Solemya velesiana gill symbiont	668
A1WZM1	General secretion pathway protein D	Hhal_2370	Halorhodospira halophila (strain DSM 244 / SL1) (Ectothiorhodospira halophila (strain DSM 244 / SL1))	670
B5JVD5	General secretion pathway protein D	gspD GP5015_375	gamma proteobacterium HTCC5015	694
B8GU41	General secretion pathway protein D	Tgr7_0225	Thioalkalivibrio sulfidiphilus (strain HL-EbGR7)	655
G0A6N9	General secretion pathway protein D	Metme_0902	Methylomonas methanica (strain MC09)	621
G4E4N3	General secretion pathway protein D	ThisiDRAFT_1262	Thiorhodospira sibirica ATCC 700588	668
I3CIS6	General secretion pathway protein D	BegalDRAFT_2678	Beggiatoa alba B18LD	695
L0E2F0	General secretion pathway protein D	xcpQ [H] TVNIR_3844	Thioalkalivibrio nitratireducens (strain DSM 14787 / UNIQEM 213 / ALEN2)	654
Q0A5Z6	General secretion pathway protein D	Mlg_2401	Alkalilimnicola ehrlichii (strain ATCC BAA-1101 / DSM 17681 / MLHE-1)	673
W0DIW0	General secretion pathway protein D	THITH_00140	Thioalkalivibrio paradoxus ARh 1	654
W6LT80	Putative general secretion pathway protein	BN874_1620022	Candidatus Contendobacter odensis Run_B_J11	699
W6ME49	Putative General secretion pathway protein D	BN873_770026	Candidatus Competibacter denitrificans Run_A_D11	768
A0A0F6RB45	General secretion pathway protein D	TQ33_0264	Kangiella geojedonensis	726
A0A1B3B888	General secretion pathway protein D	KS2013_292	Kangiella sediminilitoris	723
C7R8G0	General secretion pathway protein D	Kkor_2316	Kangiella koreensis (strain DSM 16069 / KCTC 12182 / SW-125)	708

A0A0C4WQL0	General secretion pathway protein D	gspD Achr_36010	Azotobacter chroococcum NCIMB 8003	640
A0A0F7M5J4	Type II secretion system protein D	IMCC21906_01807	Spongiibacter sp. IMCC21906	696
A0A127M4E2	Type II secretion system protein GspD	AZF00_07000	Zhongshania aliphaticivorans	721
A0A1G3CVS1	Type II secretion system protein GspD	A2002_01850	Pseudomonadales bacterium GWC1_66_9	640
A0A1N6XJ91	General secretion pathway protein D	SAMN05421647_11457	Marinobacterium stanieri	661
A0A1R1LZ24	Type II secretion system protein GspD	BGP75_14740	Motiliproteus sp. MSK22-1	653
A0A1R1M4X8	Type II secretion system protein GspD	BGP75_10840	Motiliproteus sp. MSK22-1	654
S6GPI0	General secretion pathway protein D	OFPI_02970	Osedax symbiont Rs2	690
A0YG33	General secretion pathway protein D	GP2143_02030	marine gamma proteobacterium HTCC2143	689
B7RSS9	General secretion pathway protein D	gspD_1 GPB2148_2888	marine gamma proteobacterium HTCC2148	677

Pseudomonas putida/fluorescens/syringae

Entry	Protein names	Gene names	Organism	Length
A0A0D7F839	General secretion pathway protein GspD	UM91_18010	Pseudomonas oryzihabitans	668
A0A1M6MRL4	Type II secretion system protein D (GspD)	SAMN05192556_101139	Halomonas sinaiensis	695
E1V5E8	General secretion pathway protein GspD	gspD2 HELO_3219	Halomonas elongata (strain ATCC 33173 / DSM 2581 / NBRC 15536 / NCIMB 2198 / 1H9)	700
A0A059UT06	General secretion pathway protein D	DW66_1038	Pseudomonas putida (Arthrobacter siderocapsulatus)	587
A0A061JU99	General secretion pathway protein D	B597_001430	Pseudomonas stutzeri KOS6	646
A0A067A6W0	Type II secretion system protein D	gspD BV82_0433	Pseudomonas sp. P482	650
A0A075P6X1	General secretion pathway protein GspD	HZ99_04795	Pseudomonas fluorescens	647
A0A077LH04	Type II secretion system protein D	PSCI_2484	Pseudomonas sp. StFLB209	648
A0A085VFX0	General secretion pathway protein GspD	IV01_16380	Pseudomonas syringae	648
A0A088NNN4	General secretion pathway protein GspD	O165_017680	Pseudomonas mosselii SJ10	649
A0A0A7JNJ7	General secretion pathway protein GspD	NJ69_13450	Pseudomonas parafulva	651
A0A0C5RLD8	General secretion pathway protein GspD	N805_17110	Pseudomonas putida S13.1.2	582
A0A0D0L1I8	General secretion pathway protein GspD	RU08_01260	Pseudomonas fulva	650
A0A0D1M236	General secretion pathway protein GspD	QV12_05830	Pseudomonas putida (Arthrobacter siderocapsulatus)	651
A0A0F0FE79	General secretion pathway protein GspD	UB48_10240	Pseudomonas sp. 2(2015)	650
A0A0M2UBF7	General secretion pathway protein GspD	V520_24840	Pseudomonas putida KG-4	581
A0A0P7CFV6	Type II secretion system protein GspD	HB4184_22685	Pseudomonas putida (Arthrobacter siderocapsulatus)	578
A0A0Q5F937	Type II secretion system protein GspD	ASF84_25820	Pseudomonas sp. Leaf127	656
A0A0U4VRS9	Type II secretion system protein GspD	APT59_16805	Pseudomonas oryzihabitans	670
A0A101LN13	Type II secretion system protein GspD	AR540_08080	Pseudomonas sp. EpS/L25	647
A0A126S8Z7	General secretion pathway protein D	AWT69_003970	Pseudomonas putida (Arthrobacter siderocapsulatus)	650
A0A160GLE3	General secretion pathway protein GspD	AB688_07905	Pseudomonas putida (Arthrobacter siderocapsulatus)	651
A0A176VA36	Type II secretion system protein GspD	A2T76_04975	Pseudomonas brenneri	647
A0A178L352	Type II secretion system protein GspD	A4V15_09720	Pseudomonas oryzihabitans	669
A0A1E2VEY3	Type II secretion system protein GspD	BFW38_16490	Terasakiispira papahanaumokuakeensis	662
A0A1E4UVV0	Type II secretion system protein GspD	A7D25_17035	Pseudomonas sp. 21C1	635
A0A1H2MPS8	General secretion pathway protein D	SAMN04490181_2040	Pseudomonas brenneri	649
A0A1H2MU36	General secretion pathway protein D	SAMN05216202_2392	Pseudomonas mucidolens	649
A0A1H6DFV3	General secretion pathway protein D	SAMN05444390_10677	Marinobacterium lutimaris	584
A0A1I3K0Q6	General secretion pathway protein D	SAMN05216602_2204	Pseudomonas argentinensis	650

A0A1L7N8F3	General secretion pathway protein D	KF715C_ch11720	Pseudomonas putida (Arthrobacter siderocapsulatus)	588
A0A1M7M962	General secretion pathway protein D	SAMN05660971_04104	Halomonas cupida	697
A0A1Q9XUL8	Type II secretion system protein GspD	BVH03_12020	Pseudomonas sp. PA15(2017)	627
B0KRF9	General secretion pathway protein D	PputGB1_1043	Pseudomonas putida (strain GB-1)	584
B1JDL3	General secretion pathway protein D	PputW619_4180	Pseudomonas putida (strain W619)	650
Q1I5W7	Putative type II secretion system protein D	PSEEN4281	Pseudomonas entomophila (strain L48)	651
Q52291	XcpQ protein	xcpQ	Pseudomonas putida (Arthrobacter siderocapsulatus)	591
T2HCF8	Type II secretion system protein XcpQ	xcpQ PP4_42700	Pseudomonas putida NBRC 14164	588
U5VM15	General secretion pathway protein D	PVLB_19945	Pseudomonas sp. VLB120	640
W0HEV3	Type II secretion system protein D	PCH70_42170	Pseudomonas cichorii JBC1	648
