# Supplementary material for: Structural Basis of Type 2 Secretion System Engagement between the Inner and Outer Bacterial Membranes
Source: mBio. 2017 Oct 17;8(5):e01344-17. doi: 10.1128/mBio.01344-17 (PMC5646249; doi:10.1128/mBio.01344-17)
Supplement: FIG S1 [file mbo005173525sf1.pdf]

Supplementary Figure S1

A

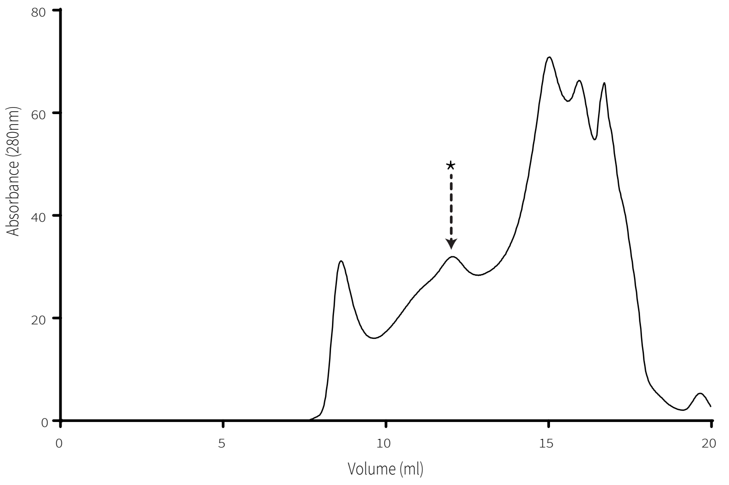

B

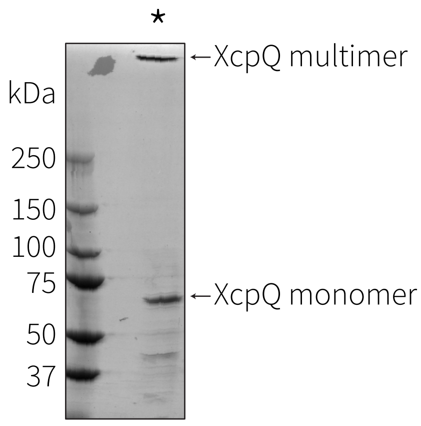

Supplementary Figure S1. Expression and purification of the XcpQ multimer. **(A)** Size-exclusion chromatograph of the Amphopol stabilised XcpQ multimer after extraction from the total cellular envelope fraction with detergent (SB3-14) and purified with nickel affinity chromatography. **(B)** The fractions marked * were assessed by SDS-PAGE and prepared for assessment by electron microscopy as described in the supplemental methods
