# Supplementary material for: Structural Basis of Type 2 Secretion System Engagement between the Inner and Outer Bacterial Membranes
Source: mBio. 2017 Oct 17;8(5):e01344-17. doi: 10.1128/mBio.01344-17 (PMC5646249; doi:10.1128/mBio.01344-17)
Supplement: FIG S2 [file mbo005173525sf2.pdf]

Supplementary Figure S2

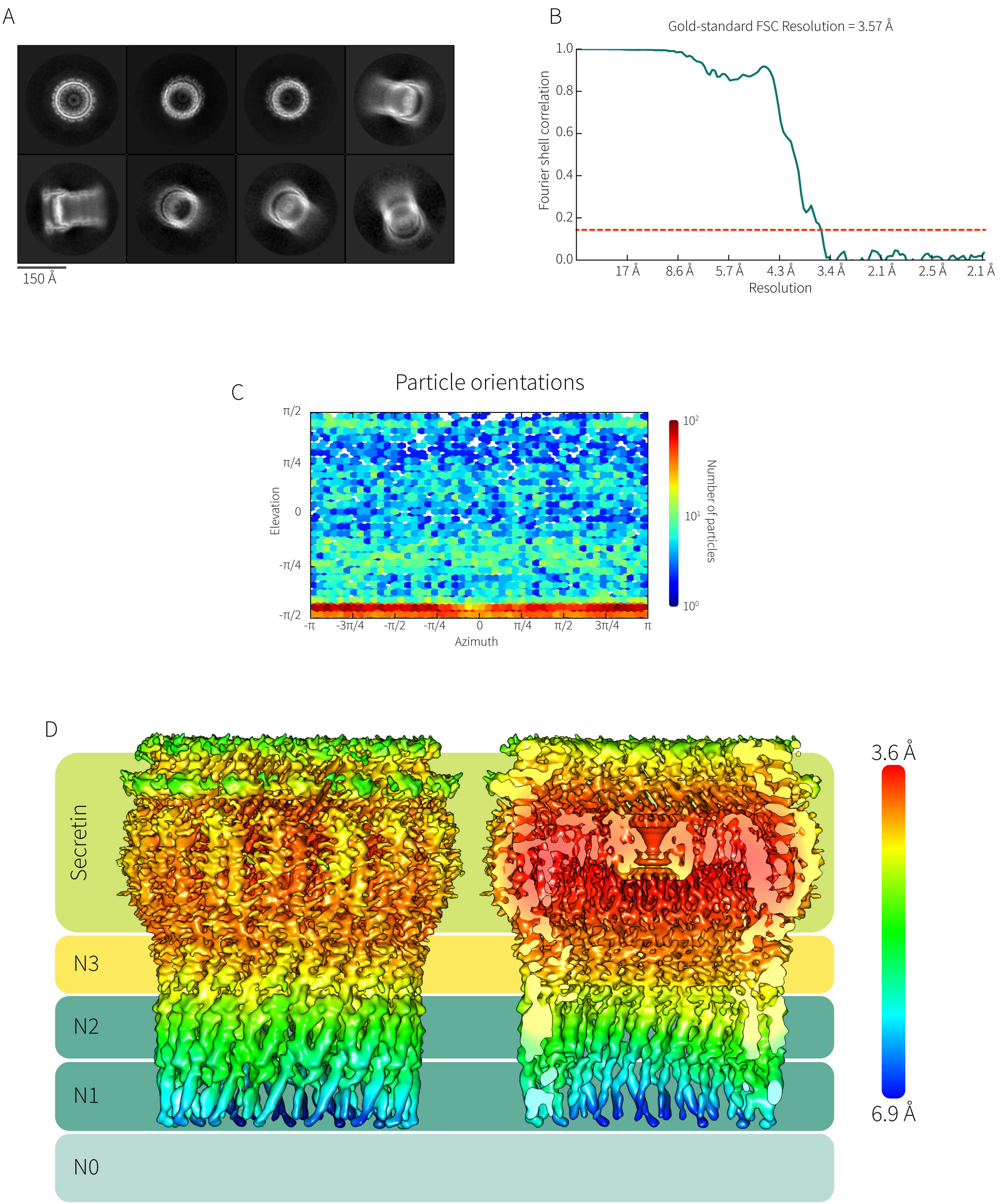

Supplementary Figure S2. Cryo EM map parameters. (A) Representative 2D class averages of XcpQ particles. **(B)** Fourier shell correlation plot of the 3D map showing a gold standard resolution of 3.57 Å (FSC=0.143 criteria). **(C)** Plot showing the distribution of particle orientations over the azimuth and elevation angles of the final map **(D)** Local resolution of the XcpQ model showing deterioration of the resolution in the N terminal domains (calculated with Relion 2.1 Local resolution).
