# Supplementary material for: Structural Basis of Type 2 Secretion System Engagement between the Inner and Outer Bacterial Membranes
Source: mBio. 2017 Oct 17;8(5):e01344-17. doi: 10.1128/mBio.01344-17 (PMC5646249; doi:10.1128/mBio.01344-17)
Supplement: FIG S3 [file mbo005173525sf3.pdf]

Supplementary Figure S3

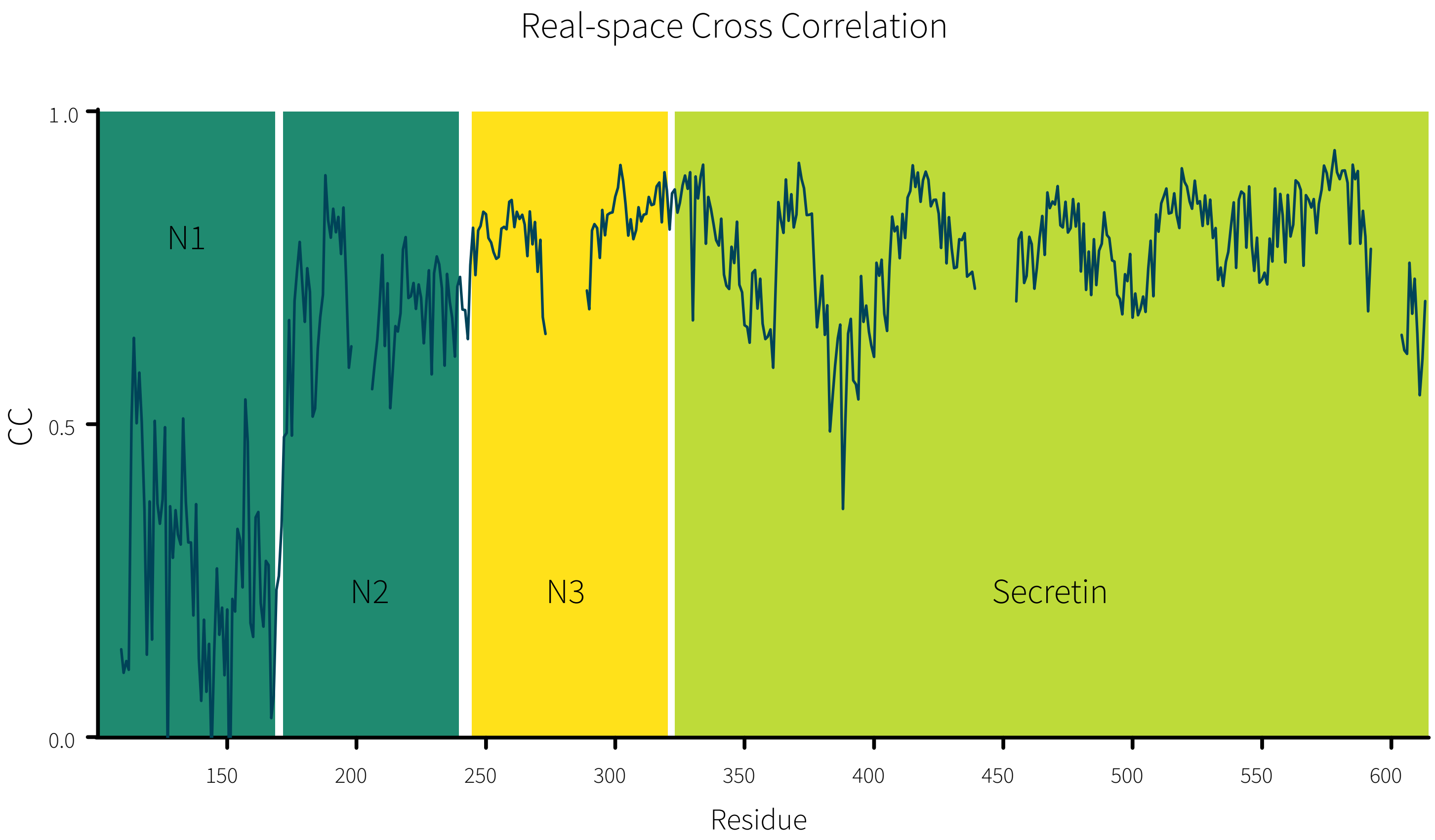

Supplementary Figure S3. Real-space Cross Correlation of the fit of the atomic model in to the electron density map
