# Supplementary material for: Structural Basis of Type 2 Secretion System Engagement between the Inner and Outer Bacterial Membranes
Source: mBio. 2017 Oct 17;8(5):e01344-17. doi: 10.1128/mBio.01344-17 (PMC5646249; doi:10.1128/mBio.01344-17)
Supplement: TABLE S2 [file mbo005173525st2.pdf]

Supplemental Table S2 Model Parameters

**Data Collection**	
Particles	18,005
Pixel size	0.53
Defocus range	0.6-2.5
Voltage (kV)	300
Electron Dose (e/Å^2)	40
**Refinement**	
CC	0.723
**Model Quality**	
RMSD	
Bond length(Å)/Bond angles	0.009/1.379
Ramachandran	
Favoured (%)	89.12
Outliers (%)	0.0
Rotamer outliers	0.31
C-beta deviations	0
Clashscore	7.2
